# A C_66_ Polycyclic Aromatic Hydrocarbon with Six Azulene Units and NIR-II Absorption: Toward Azulene-Based Carbon Allotropes

**DOI:** 10.1021/jacs.6c04336

**Published:** 2026-04-15

**Authors:** Maksymilian Borkowski, Ada Drwęcka, Szymon J. Zelewski, Artur Kasprzak, Sławomir Szafert, Bartłomiej Pigulski

**Affiliations:** † Faculty of Chemistry, University of Wrocław, Joliot Curie 14, 50-383 Wrocław, Poland; ‡ Department of Experimental Physics, Faculty of Fundamental Problems of Technology, Wrocław University of Science and Technology, 50-370 Wrocław, Poland; § Faculty of Chemistry, Warsaw University of Technology, Noakowskiego 3, 00-664 Warsaw, Poland

## Abstract

The development of small-molecule dyes with strong and tunable absorption in the near-infrared II (NIR-II, 1000–1700 nm) window remains a central challenge for optoelectronic and biomedical applications, motivating the exploration of unconventional π-conjugated frameworks. In this context, nonalternant polycyclic aromatic hydrocarbons (PAHs) have emerged as promising candidates, with azulene-embedded architectures offering access to small electronic gaps. However, the lack of efficient synthetic strategies has largely restricted the use of such systems, reducing systematic structure–property relationships and limiting their use as functional NIR-active materials. Here, we report the synthesis of **2-diMes**, a C_66_ PAH that represents the first nonalternant PAH incorporating six azulene subunits within a fully conjugated framework. This purely hydrocarbon, closed-shell analogue of hexabenzoperylene (HBP) exhibits an exceptionally small HOMO–LUMO gap, among the smallest reported for neutral closed-shell π-conjugated hydrocarbons. The nonalternant topology of **2-diMes** gives rise to intense near-infrared absorption with an onset extending to 1450 nm (0.85 eV) and a small electrochemical gap of 1.13 V. Notably, **2-diMes** functions as a highly photostable NIR-II photoacoustic dye, combining a strong photoacoustic response with excellent solubility in common organic solvents. Spectroelectrochemical and titration studies revealed further red-shifted absorption upon oxidation, with the radical cation **[2-diMes]^•+^** absorbing beyond 3200 nm (0.39 eV) and the dication **[2-diMes]** ^**2+**^ absorbing up to 2600 nm (0.48 eV). These results highlight nonalternant azulene-rich PAHs as a powerful and underexplored platform for the rational design of next-generation NIR-II dyes and functional organic materials.

## Graphical abstract

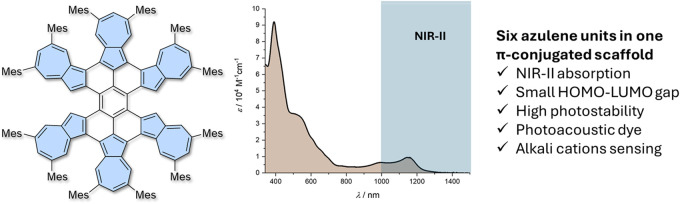

## Introduction

Larger polycyclic aromatic hydrocarbons (PAHs), commonly termed nanographenes, have shown great potential in the field of organic electronics because of their optical and electronic properties.^1,2^ However, the chemistry of such sp^2^-conjugated systems is not restricted only to benzenoid structures, and embedding odd-membered rings in π-conjugated scaffolds results in a nonalternant topology.^3^ Importantly, nonalternant PAHs possess drastically different properties as compared to their benzenoid analogues. Azulene is a seminal example of the drastic change of properties in nonbenzenoid PAHs when compared with isomeric naphthalene. Contrary to naphthalene, azulene possesses a large dipole moment (1.08 D)^4^ and optical absorption in the visible range^5^ and exhibits *anti*-Kasha emission from the S_2_ excited state.^6^ Consequently, embedding of such a nonalternant structural motif in larger nanographenes leads to new, exciting properties such as NIR absorption,^7,8^ a small HOMO–LUMO gap,^8^ *anti*-Kasha fluorescence,^9,10^ and biradical character.^11,12^ The synthesis of various azulene-embedded π-scaffolds^13,14^ is a rapidly growing research area in organic chemistry due to their application in material chemistry^15,16^ and fundamental exploration of new carbon allotropes. Recently, many 2D graphenoid azulene-based allotropic forms of carbon were theoretically predicted like a family based on azulenoid kekulene,^17^ phagraphene,^18^ TPH-graphene,^19^ PHH-graphene,^20^ and ψ-graphene.^21^

Importantly, these nonalternant graphenoid materials are predicted to exhibit smaller band gaps than their benzenoid counterparts, underscoring the potential of azulene-based topologies for low-band-gap electronic materials.^19^ In line with this conceptual framework, a diverse range of azulene-embedded nanographenes has been reported in recent years.^13^ Representative examples include azulene-containing isomers of helicenes,^22^ linear acenes,^10,23–25^ extended pentalenes,^26,27^ and rylenes,^7,8,28^ many of which display unusual electronic or optical behavior. In addition, larger warped azulene-containing PAHs have been accessed through late-stage Scholl-type oxidative cyclization,^29–31^ providing further opportunities to tailor molecular curvature and electronic structure. More recently, heteroatom-doped azulene motifs embedded within extended PAHs have also been explored as an additional handle to fine-tune electronic properties.^32–34^

Despite the rapid progress in the synthesis of various azulene-embedded PAHs, the fusion of multiple azulene subunits within a single framework remains a significant synthetic challenge and well-defined conjugated structures containing more than two azulene units are exceedingly rare.^35^ To the best of our knowledge, only PAHs incorporating up to four fused azulene subunits are known to date. A larger number of azulene subunits in one π-conjugated skeleton was reported only for polydisperse carbon nanoribbons^36^ and single molecules constructed using on-surface chemistry.^19,37^ Tani and co-workers reported the only known PAH featuring four fused azulene units via Scholl-type oxidative cyclodehydrogenation (Figure 1).^38^ The utility of the Scholl reaction as a key synthetic tool has also been demonstrated by Uno in the construction of an azulene-embedded analogue of hexapyrrolohexaazacoronene.^39^ Alternatively, intramolecular palladium-catalyzed C–H arylation offers a viable approach to fusing multiple azulene units within nanographene frameworks. Liu and co-workers in 2023 employed this method to synthesize N-doped azulene-embedded nanographene, although the reactions proceeded in a very low yield (Figure 1).^40^ In 2025, the same group applied this methodology in the synthesis of purely hydrocarbon PAHs containing three azulene subunits.^41^ Synthesis of PAHs with three embedded azulene subunits using Suzuki coupling followed by Knoevenagel condensation was also reported independently by Liu and Mastalerz.^42,43^

**Figure 1:**
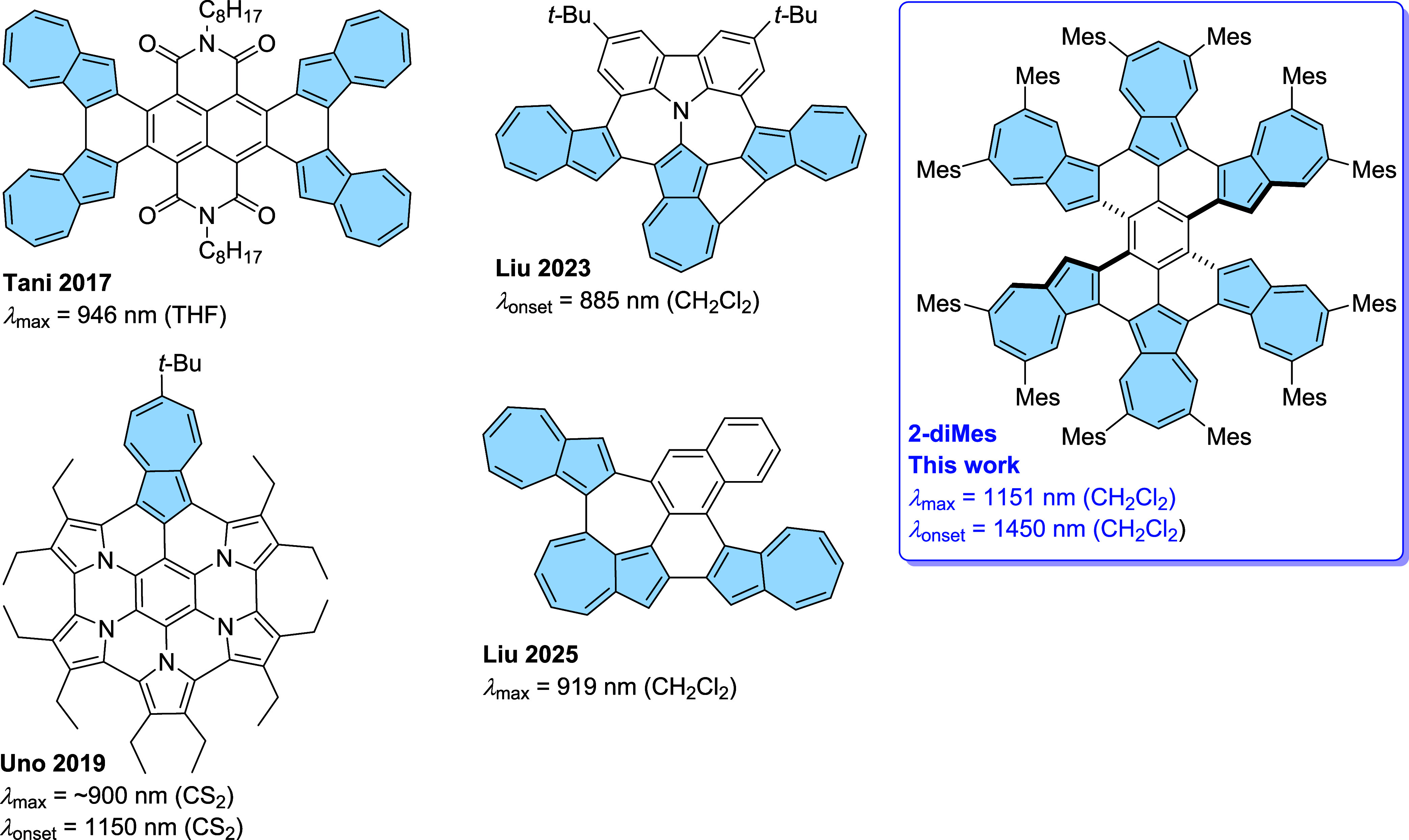
Examples of monodispersed azulene-embedded PAHs, **2-diMes**, and their optical absorption.

Organic dyes absorbing in the second near-infrared window (NIR-II, 1000–1700 nm) are of growing interest due to their desired physicochemical properties and broad applicability in biotechnology, including bioimaging, photodynamic therapy (PDT), photothermal therapy (PTT), and photoacoustic imaging.^44,45^ Despite advances in NIR dye development, stable organic chromophores with strong NIR-II absorption remain rare. Current strategies primarily rely on heteroatom-doped *push*–*pull* systems,^45–48^ large rylenes,^49–51^ or π-extended porphyrinoids,^52–54^ and yet achieving both low-energy absorption and structural stability in purely hydrocarbon systems is challenging. Nonbenzenoid π-conjugated systems incorporating azulene subunits are distinguished by their intrinsically small HOMO–LUMO gaps, which can induce red-shifted NIR absorption and unusually small electrochemical gaps, features that are difficult to realize in closed-shell neutral hydrocarbons of comparable size. Accordingly, absorption onsets beyond 1000 nm have been reported for compounds by Tani^38^ and Uno^39^ (Figure 1) and a limited number of systems containing one^7,19^ or two^8^ azulene subunits. However, the resulting NIR absorption is typically weak due to the partially forbidden nature of the HOMO–LUMO transition.

Here, we extend this conceptual approach by adopting the design strategies previously applied to fully and partially fused hexabenzocoronenes (HBCs) and hexapyrrolohexaazacoronenes (HPHACs), which are key molecules in the field of π-conjugated materials. In pursuit of NIR-absorbing systems, a lower-symmetry, not fully fused analogue of HBC represents a more suitable target, as highly symmetric HBCs exhibit nearly forbidden lowest-energy transitions, in contrast to partially fused hexabenzoperylene (HBP).^55^ A similar red shift of the lowest-energy absorption might be observed in HBC lacking one C–C bond.^56^ Additionally, the HBC-like synthetic approach gives access to structures with multiple azulene subunits with a relatively small number of steps. We realized this approach by embedding six azulene subunits into a purely hydrocarbon nanographene, giving rise to **2-diMes** (Figure 1), a nonalternant analogue of hexabenzoperylene. This molecule represents the first nanographene with such a high number of azulene subunits, resulting in a uniquely small HOMO–LUMO gap, strong NIR-II absorption (λ_max_ = 1151 nm, ε = 9600 M^–1^ cm^–1^, λ_onset_ = 1450 nm), and a narrow electrochemical gap (1.13 V), which is far more red-shifted than typical azulene-embedded PAHs. The oxidized species of **2-diMes** display extremely low-energy optical transitions, extending beyond 3200 nm for the radical cation **[2-diMes]^•+^** and up to 2600 nm for the dication **[2-diMes]** ^**2+**^. These pronounced NIR properties enable the first exploration of azulene-embedded PAHs as photoacoustic dyes, highlighting the potential of **2-diMes** as a platform for stable NIR-II-active organic materials for technological applications.

## Results and Discussion

### Synthesis

Our synthetic approach to polycyclic aromatic hydrocarbons (PAHs) containing multiple azulene subunits was inspired by the classical synthesis of hexabenzocoronenes (HBCs)^57^ and hexapyrrolohexaazacoronenes (HPHACs).^58^ The central idea of our strategy was to exploit the ease of oxidation at the 1- and 3-positions of the azulene subunits to construct fused, azulene-embedded scaffolds in an analogous fashion. Very recently, we improved the synthetic route to substituted hexakis­(azulen-2-yl)­benzenes^59^ which represent logical precursors of multiazulene π-conjugated scaffolds upon Scholl-type oxidative cyclodehydrogenation in a similar manner like HBC and HPHAC. Our initial efforts focused on the oxidation of hexakis­(azulen-2-yl)­benzenes derived from 6-*tert*-butylazulene and 6-mesitylazulene. However, despite extensive attempts, these substrates underwent only decomposition under oxidative conditions with no desired product observed. It gave us a hint why no oxidation of hexakis­(azulen-2-yl)­benzenes was reported since the first synthesis by Ito over 20 years ago.^60^ To address this limitation, we designed a more robust 5,7-dimesitylazulene building block bearing sterically hindered substituents to enhance the stability of the final product and to gain more control over the oxidation reaction (Scheme 1).

**Scheme 1:**
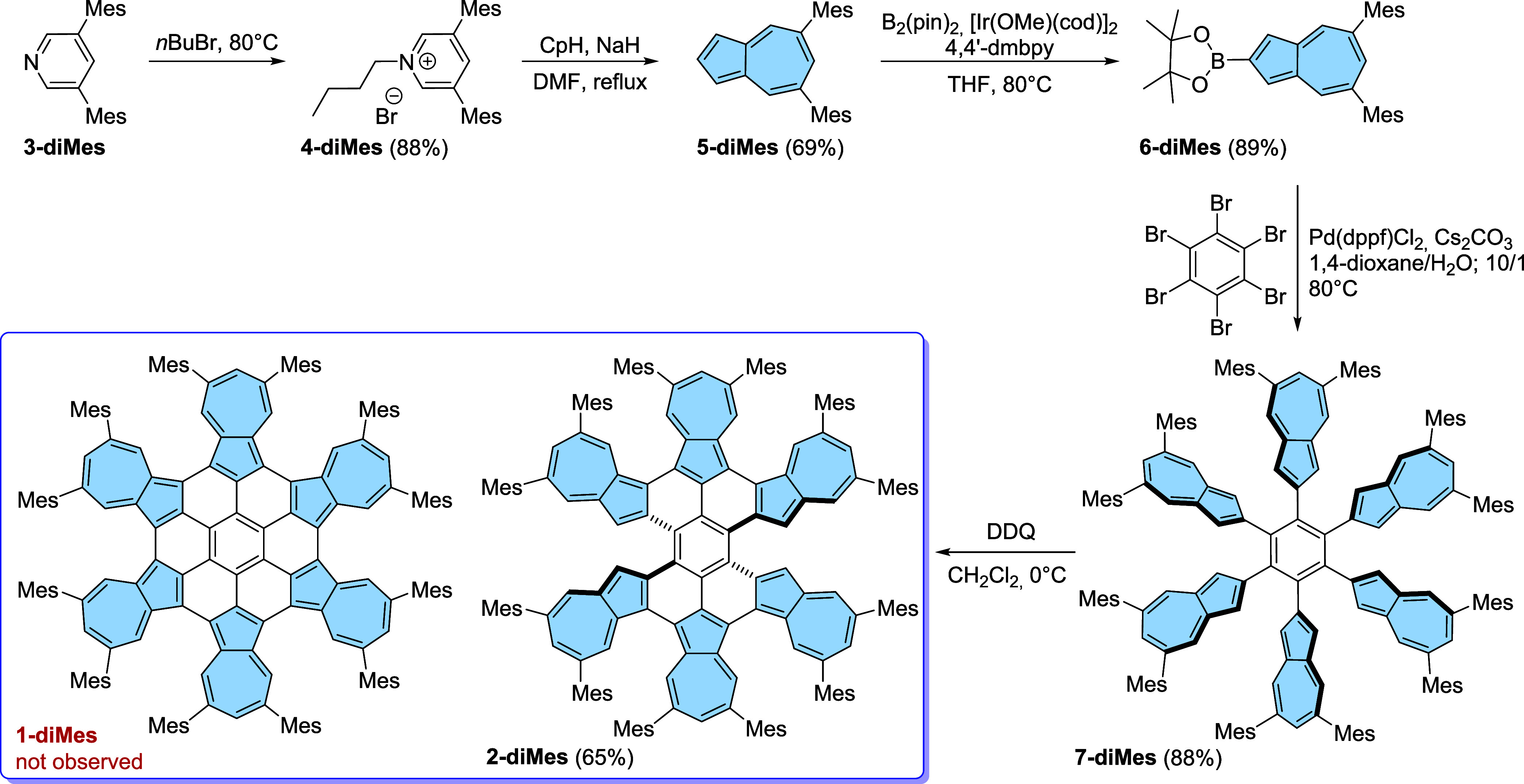
Synthesis of PAH **2-diMes** Containing Six Azulene Subunits^a^

a Mes = mesityl, CpH = cyclopentadiene, DMF = dimethylformamide, pin = pinacolato, cod = 1,5-cyclooctadiene, 4,4′-dmbpy = 4,4′-dimethyl-2,2′-bipyridyl, THF = tetrahydrofuran, dppf = 1,1′-bis­(diphenylphosphino)­ferrocene, DDQ = 2,3-dichloro-5,6-dicyano-1,4-benzoquinone.

The synthesis started from the known 3,5-dimesitylpyridine (**3-diMes**),^61^ which was reacted with *n*-bromobutane to afford the corresponding pyridinium salt **4-diMes** in an 88% yield (Scheme 1). The pyridinium salt **4-diMes** was then treated with *in situ*-generated sodium cyclopentadienide in a Ziegler–Hafner azulene synthesis,^62^ yielding the target 5,7-dimesitylazulene **5-diMes** in a 69% yield. Subsequently, **5-diMes** was subjected to Ir­(I)-catalyzed C–H borylation under modified literature conditions,^63–65^ leading to the regioselective formation of **6-diMes**, borylated at the 2-position in an 89% yield. Finally, a 6-fold Suzuki cross-coupling reaction between **6-diMes** and hexabromobenzene furnished the hexakis­(azulen-2-yl)­benzene **7-diMes** in an 88% yield. With the direct precursor in hand, a vast spectrum of known procedures for Scholl-type oxidative dehydrogenation^66,67^ was tested (SI, Table S1). Despite numerous attempts, the fully oxidized compound **1-diMes** was not observed in any case. However, when DDQ was employed as an oxidant, we observed the selective formation of a product of a 4-fold reaction, **2-diMes**. After optimization of the reaction conditions, the nanographene **2-diMes** was isolated finally in a 65% yield. Any attempt to further oxidize **2-diMes** to **1-diMes** failed, probably due to the steric hindrance of mesityl substituents and the strain of the π-conjugated core (SI, Table S2). In addition, we analyzed the DFT-calculated spin density distribution of the intermediate radical cation following the literature procedure.^68^ The results revealed no positive spin density at the carbon atoms required for the formation of the two additional C–C bonds (SI, Figure S52), thereby further rationalizing the selective formation of **2-diMes**. The reversibility of the first two oxidations of **2-diMes** observed by CV (cyclic voltammetry) further supports the theoretical results. The selective isolation of not fully oxidized products was reported also during the synthesis of derivatives of HPHACs^69^ and monoazulene-embedded HBC.^70^ It is worth noting that **1** was for the first time proposed by Ito over two decades ago^60^ but no synthetic attempts toward **1** have been reported to date. We also tested additional synthetic approaches utilizing chlorinated **7-diMes** followed by Yamamoto-type homocoupling (SI, Figure S1). However, in this case, we could not synthesize the direct chlorinated precursor of **1-diMes**.

The ^1^H NMR spectrum of **2-diMes** exhibited a set of sharp signals and indicated high effective NMR symmetry of the conjugated core in solution, despite its warped geometry and the presence of six formally defined helicene subunits, which could potentially give rise to multiple pairs of enantiomers (Figure 2). However, the racemization barriers are low enough that **2-diMes** exhibits *pseudo D*_2_ NMR symmetry at room temperature in a similar manner to some other large warped nanographenes.^71,72^ Additionally, a set of sharp signals clearly indicates the closed-shell character of the PAH. We carried out additional variable-temperature ^1^H NMR measurements in CH_2_Cl_2_ in the temperature range from 290 to 180 K (SI, Figure S38) due to the high freezing point of C_6_D_6_. Even at 180 K, we observed only slight sharpening of the peaks and no significant change in the effective NMR symmetry of the indicative signals of the π-conjugated core. It indicates that the conformation of **2-diMes** is not frozen even at 180 K and the energy barrier between various conformers is very low. The formation of **2-diMes** was additionally confirmed through APCI-HRMS, with a M + H^+^ ion peak of *m*/*z* = 2245.2103 (SI, Figure S32) and isotope patterns in accordance with a molecular formula of C_174_H_155_ (calculated *m*/*z* = 2245.2157). PAH **2-diMes** is soluble in most of the common organic solvents including hexane and CH_2_Cl_2_.

**Figure 2:**
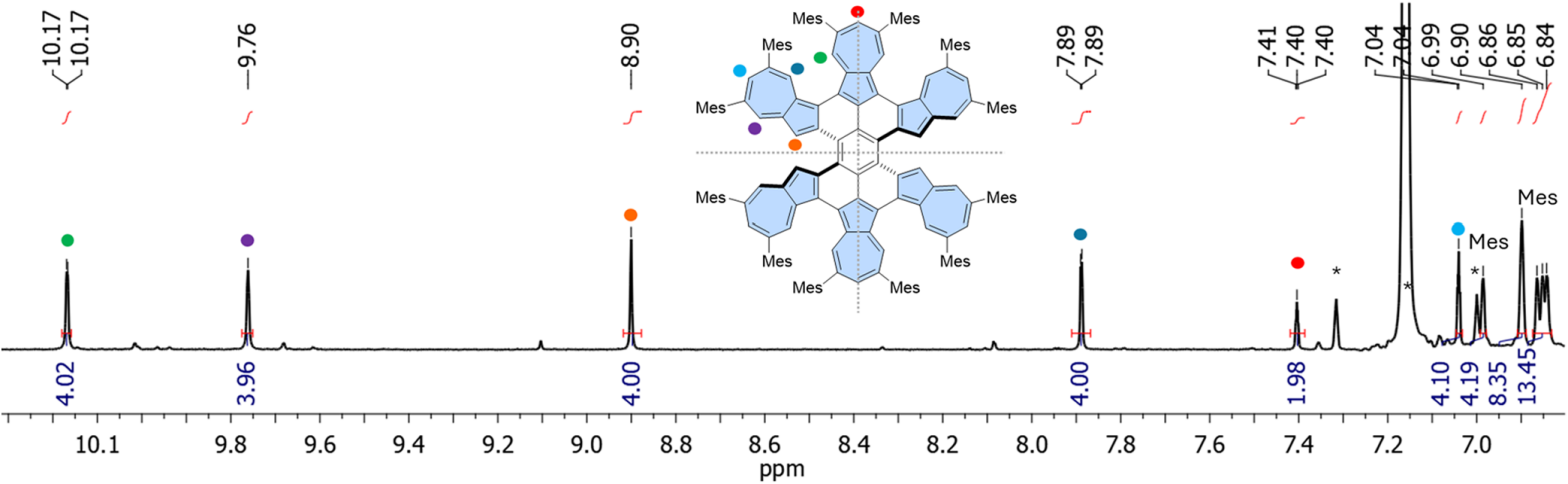
Aromatic region of the ^1^H NMR spectrum of **2-diMes** (C_6_D_6_, 500 MHz, 300 K).

### X-ray Single-Crystal Diffraction

Both **7-diMes** and **2-diMes** were characterized by single-crystal X-ray diffraction. Suitable crystals were obtained by the vapor diffusion of MeOH into their solutions in toluene. The compound **7-diMes** crystallized in the *C*2/*c* space group, a monoclinic system, with half a molecule in the asymmetric unit. The molecular structure of **7-diMes** adopts a propeller-like shape; however, due to steric hindrance from the mesityl substituents, no columnar stacking is observed in the solid state, unlike for less-substituted hexakis­(azulen-2-yl)­benzenes^59^ (Figure 3a). PAH **2-diMes** crystallized in the *P*-1 space group, a triclinic system, with a significant amount of toluene in the crystal lattice. The crystal structure provides an unambiguous structural assignment of **2-diMes** and solid proof for the selective formation of four new C–C bonds (Figure 3b). The intramolecular distances between carbons needed to form fully oxidized **1-diMes** are above 3 Å (Figure 3b), which provides an additional explanation for why the reaction stops at **2-diMes**. The conjugated scaffold of **2-diMes** is strongly warped and adopts two enantiomeric conformations in the solid state from the multiple possible configurations of its six [5]­helicene subunits. In the crystal, the two observed enantiomers are (*M*,*M*,*P*,*P*,*M*,*P*) and (*P*,*P*,*M*,*M*,*P*,*M*), consistent with crystallization in the centrosymmetric *P*-1 space group. Notably, the symmetry observed in the solid state is lower than that inferred from ^1^H NMR, reflecting the conformational flexibility of **2-diMes**. Though frozen in a single conformation by crystal packing constraints in the solid state, this 17-ring carbon framework is highly flexible in solution and changes its shape constantly, giving relatively high NMR symmetry. The dihedral angles between the planes defined by the terminal rings of the two central [5]­helicene moieties with M helicity are 47.0 and 60.6°, which are more distorted than the four outer [5]­helicene units, ranging from 29.4 to 40.7° (Figure 3c). The central benzene ring is significantly twisted, with torsion angles from 9.98 to 22.72° and a HOMA (harmonic oscillator model of aromaticity) value of 0.667 (HOMA = 1 for an undistorted benzene ring).^73^ Adjacent benzene rings are similarly distorted, with HOMA values ranging from 0.499 to 0.610. Azulene units are also notably distorted with several experimental bond lengths differing from those in pristine azulene. Overall, the conjugated skeleton of **2-diMes** is highly warped (Figure 3d). DFT calculations of various conformers of **2** without mesityl groups (SI, Figure S51) suggest that the (*P*,*M*,*P*,*P*,*M*,*P*)/(*M*,*P*,*M*,*M*,*P*,*M*) pair of the enantiomeric pair of conformers possesses the lowest energy and was used for all theoretical analyses presented in this work despite the fact that the (*M*,*M*,*P*,*P*,*M*,*P*)/(*P*,*P*,*M*,*M*,*P*,*M*) pair of conformers has slightly higher energy.

**Figure 3:**
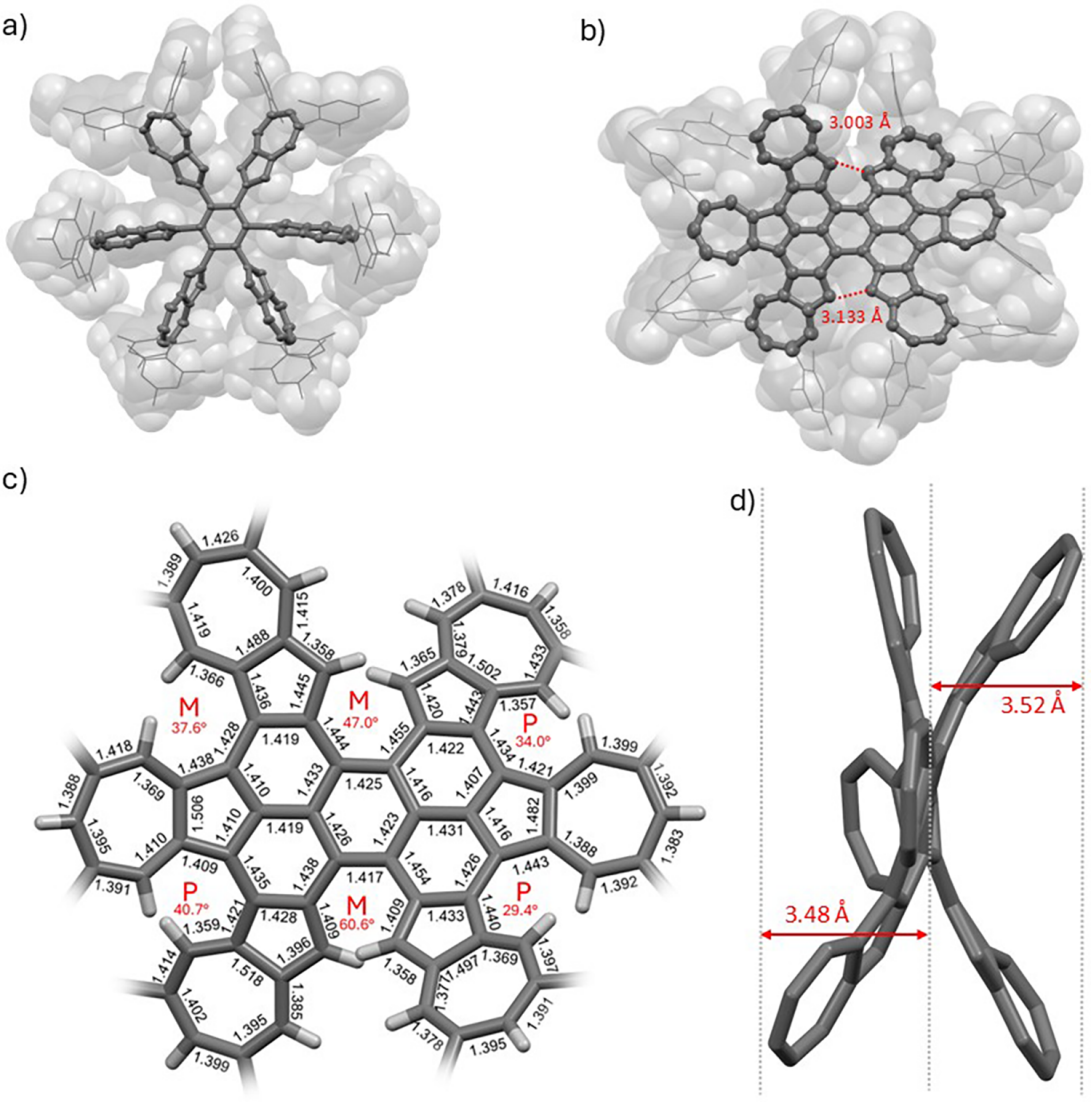
Molecular structures of (a) **7-diMes** and (b) **2-diMes**; ellipsoids set at 50% probability, mesityl substituents presented as a wireframe and hydrogens omitted for clarity, disorder omitted for clarity. (c) Selected bond lengths (Å) for **2-diMes** (black); dihedral angles between planes of terminal rings of [5]­helicenes (red), mesityl groups omitted for clarity. (d) Side view of **2-diMes**; mesityl groups and hydrogens omitted for clarity.

### Optical and Electronic Properties

The optical properties of the nanographene **2-diMes** were investigated by UV/vis/NIR absorption spectroscopy in dichloromethane at room temperature (Figure 4a). **2-diMes** displays an intense lowest-energy absorption band at λ_max_ = 1151 nm (1.08 eV, ε = 9600 M^–1^ cm^–1^), representing a pronounced bathochromic shift relative to benzenoid nanographenes of comparable size, such as C_60_ (λ_max_ = 597 nm),^74^ and even the largest known benzenoid systems, C_258_ (λ_max_ = 813 nm)^74^ and C_258_ (λ_max_ = 659 nm).^75^ The absorption intensity is notably stronger than that of the nearly forbidden S_0_ → S_1_ electronic transition characteristic of azulene. The change of absorption properties compared to the substrate is large since **7-diMes** exhibits typical azulene absorption around 600 nm. This remarkable red shift and enhanced intensity indicate substantial π-extension and a markedly reduced HOMO–LUMO gap arising from the integration of nonbenzenoid azulene units into the conjugated scaffold. These findings unambiguously demonstrate that the integration of multiple azulene units within a single π-conjugated framework exerts a profound effect on the electronic structure, leading to substantial modulation of the optical properties. PAH **2-diMes** is not fluorescent like many of the extended azulenes.^35^

**Figure 4:**
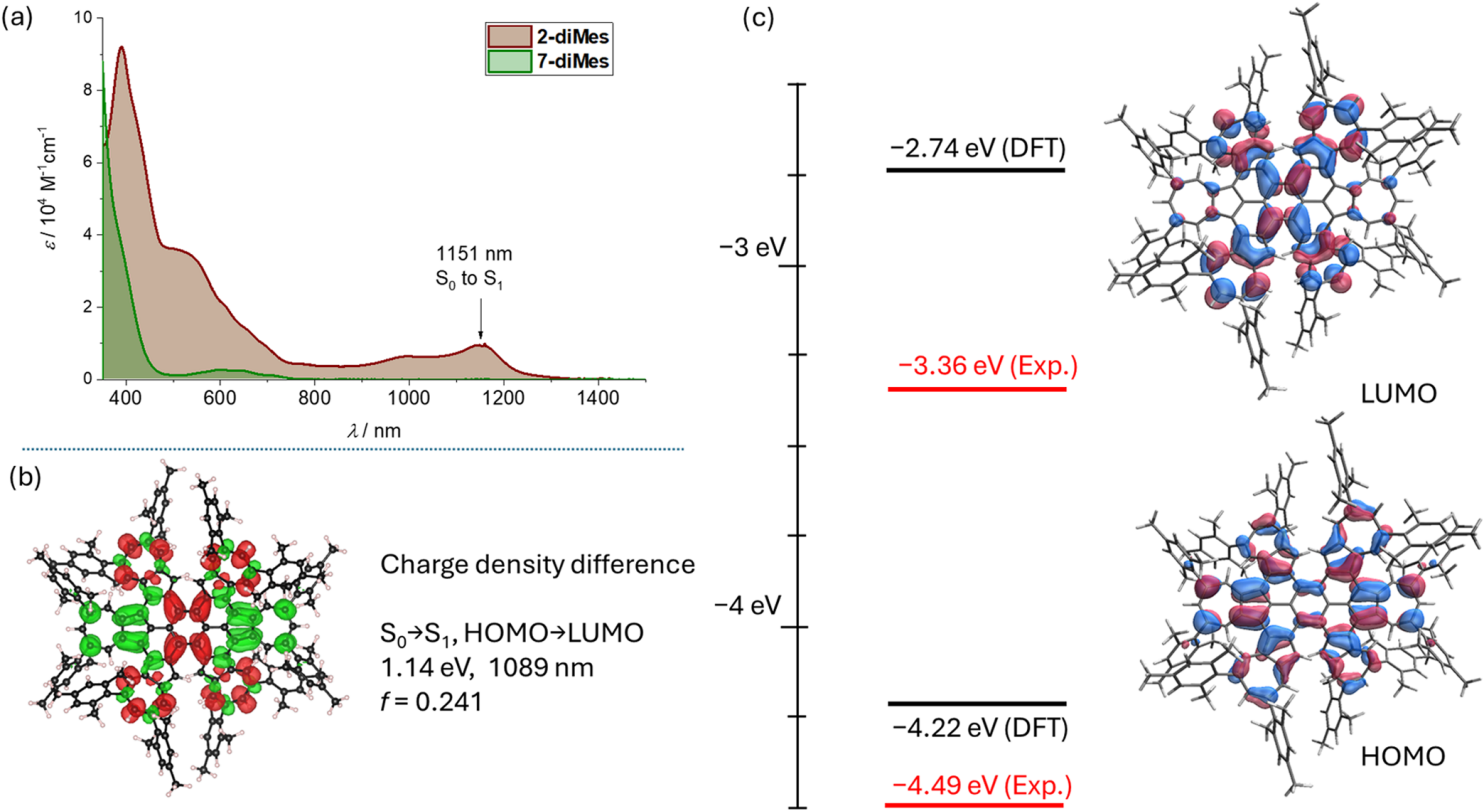
(a) UV/vis/NIR absorption spectra of **7-diMes** and **2-diMes** (CH_2_Cl_2_, 20 °C, *c* ∼ 10^–6^ M^–1^). (b) Charge density difference (CDD) plot for the S_0_ → S_1_ transition; B3LYP/6–31G­(d,p), isovalue 0.0004 au for CDD. Green (positive) and red (negative) regions, respectively, represent decreases and increases of the electron density after electronic excitation. (c) DFT-calculated (black, B3LYP/6–31G­(d,p), isovalue 0.05 A^–3^) and experimental energies of HOMO and LUMO orbitals of **2-diMes** calculated using the experimentally determined redox potentials (*E*_LUMO_ = −(*E*_red_ + 4.8 eV) and *E*_HOMO_ = −(*E*_ox_ + 4.8 eV)) and the energy level of Fc/Fc^+^ with respect to the vacuum level (−4.8 eV).^76^

DFT calculations provided further insight into the optical properties of **2-diMes**. The HOMO and LUMO distributions are shown in Figure 4c. The HOMO is primarily localized on the azulene moieties and closely resembles the frontier molecular orbital of pristine azulene. In contrast, the LUMO is mainly localized on four azulene subunits and the central benzene ring. TD-DFT calculations confirmed that the S_0_ → S_1_ transition corresponds to a HOMO–LUMO excitation, which accounts for the lowest-energy absorption band (SI, Table S7). To further investigate the nature of this transition, we computed the charge density difference (CDD), which illustrates regions of the electron density gain and loss upon excitation (Figure 4b). The CDD plots for the top and bottom azulene subunits resemble the S_0_ → S_1_ transition of pristine azulene.^7^ However, the transition involving the two central azulene units displays a charge transfer character with the electron density shifting toward the central benzene ring upon excitation. The DFT-calculated maximum of the S_0_ → S_1_ transition (1089 nm, 1.14 eV, *f* = 0.241) is in good agreement with the experimental value (1.13 eV). Thus, an exceptionally low energy of the HOMO to LUMO transition of **2-diMes** might be explained by both retaining the azulene electronic structure of four azulene subunits and, at the same time, intramolecular charge transfer upon electronic excitation, with two azulene subunits acting like donors. Finally, the spatial separation between the HOMO and the LUMO results in a low energy of the electronic transition. To provide a broader context for the optical absorption of **2-diMes**, we compared its properties with DFT-calculated values for fully oxidized **1-diMes** (SI, Table S6). The HOMO and LUMO energies of both **1-diMes** and **2-diMes** remain very similar (SI, Figures S47 and S48); however, differences in symmetry significantly affect their optical absorption. The DFT-calculated S_0_ → S_1_ transition of **1-diMes** (HOMO–LUMO, 1057 nm, 1.17 eV) has only slightly higher energy than the corresponding transition in **2-diMes**, but it is completely symmetry-forbidden (*f* = 0.000) and would likely be almost invisible. Similar, almost forbidden, S_0_ → S_1_ transitions are observed for other highly symmetric PAHs like HBCs.^77^ It is known that for HBC derivatives also, lowering the symmetry by partial fusion or distortion of geometry leads to more pronounced red-shifted absorption.^55,78^ It clearly indicates that high-symmetric molecules like **1-diMes** are less optimal molecular designs for NIR-II dyes compared to partially fused **2-diMes**.

Cyclic voltammetry (CV) measurements revealed that **2-diMes** exhibits a reversible first two oxidation waves at −0.31 and −0.03 V and a pseudoreversible reduction wave with half-wave potential at −1.44 V (Figure 5a). Peak widths at half height of the two first oxidations from DPV (differential pulse voltammetry) are 98 and 90 mV, which are in alignment with the typical one-electron process. In addition, the reversibility of the first two oxidations also agrees with the observation that **2-diMes** cannot be further oxidized using the Scholl reaction. Such easy oxidation indicates a very high experimental HOMO level, which equals −4.49 eV (calculated using Fc/Fc^+^ = −4.8 eV with respect to the vacuum level). The HOMO–LUMO gap estimated from electrochemistry (1.13 eV) is among the smallest reported to date among neutral, closed-shell hydrocarbons. The DFT-calculated HOMO level (−4.22 eV) is in good agreement with the experiment (Figure 4c), while the calculated LUMO level (−2.74 eV) is significantly different since DFT often is not very precise in predicting energies.^79^ Despite the high HOMO level and the small HOMO–LUMO gap, the compound **2-diMes** is stable in solution for days and for months as a solid at room temperature. Placing the properties of **2-diMes** in context, C_258_, which is the largest conjugated benzenoid polycyclic aromatic hydrocarbon ever prepared using solution chemistry, has an electrochemical gap equal to 1.96 eV (*E*_ox_ = 0.52 V and *E*_red_ = −1.44 V, vs Fc/Fc^+^).^75^ Only very few of the nonbenzenoid nanographenes possess comparable electronic properties, like recently reported dicyclohepta­[*cd*,*fg*]-*as*-indacene with an electrochemical gap equal to 1.31 eV (*E*_ox_ = −0.10 V, *E*_red_ = −1.41 V, vs Fc/Fc^+^).^8^

**Figure 5:**
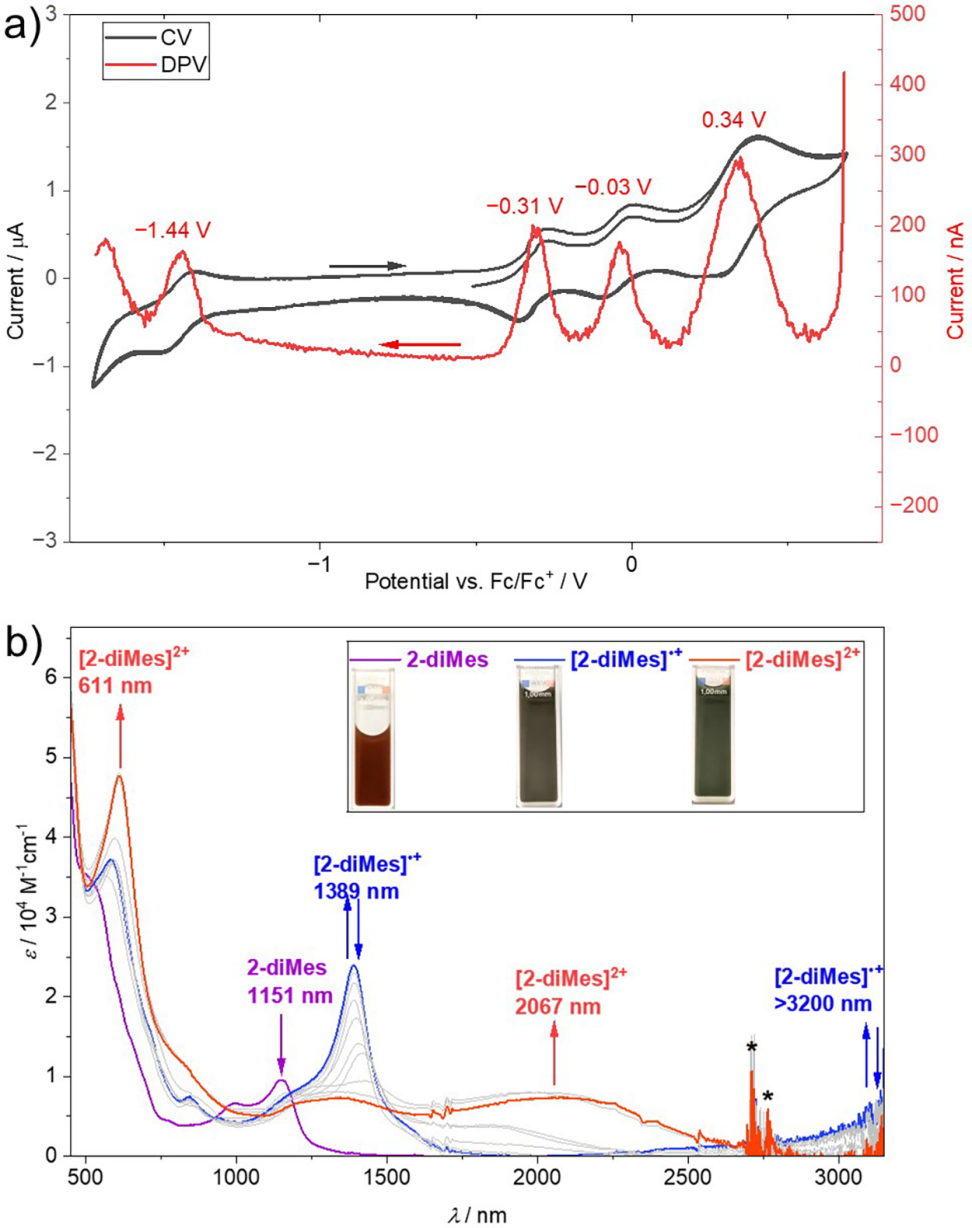
(a) Cyclic voltammograms (CVs, black) and differential pulse voltammograms (DPVs, red) of **2-diMes** (0.1 M [NBu_4_]­[PF_6_] in CH_2_Cl_2_). (b) UV/vis/NIR spectra of **2-diMes** upon titration using “magic blue” (tris­(4-bromophenyl)­ammoniumyl hexachloroantimonate), CH_2_Cl_2_, 20 °C.

The reversibility of the first two oxidation events prompted us to investigate the properties of the oxidized forms of **2-diMes**. Oxidative titrations were performed using “magic blue” (tris­(4-bromophenyl)­ammoniumyl hexachloroantimonate) as the oxidant (Figure 5b), and the experimental data were supported by TD-DFT calculations of the optical absorption spectra. Upon oxidation, the characteristic absorption band of neutral **2-diMes** at 1151 nm disappears, accompanied by the emergence of a sharp band at 1389 nm and a broad absorption extending beyond 3200 nm. Based on TD-DFT calculations (3184 nm, *f* = 0.101), these features are assigned to the radical cation **[2-diMes]** ^**•**^ **^+^**. As a result, **[2-diMes]** ^**•**^ **^+^** exhibits absorption across nearly the entire spectral range from UV to 3200 nm. Further oxidation leads to the disappearance of these bands and the appearance of a new broad absorption centered at 2067 nm, which is attributed to the dication **[2-diMes]** ^**2+**^ in agreement with TD-DFT predictions (1917 nm, *f* = 0.338). Consistent trends were also observed in spectroelectrochemical measurements (SI, Figure S46).

It is known that in many cases, retaining the aromaticity of azulene subunits is crucial for the presence of properties like NIR absorption in extended azulenes.^35^ Thus, we calculated NICS(1)_*zz*_ (nucleus-independent chemical shift)^80^ values for **2-diMes** to assess the aromaticity in the π-conjugated skeleton (Figure 6). The central benzene ring is still aromatic despite significant distortion, whereas the newly formed hexagons are nonaromatic with slightly negative NICS values. All azulene subunits remain aromatic; however, the aromaticity of all pentagons is at the same level, but heptagons of two middle subunits are more aromatic (NICS(1)_*zz*_ = −18.63) than two top and down subunits (NICS(1)_*zz*_ = −12.19 and −14.82). Such a significant difference between azulene subunits is not present in fully oxidized **1-diMes** (SI, Figure S53). Upon oxidation to the radical cation **[2-diMes]** ^**•+**^, all central hexagons become slightly aromatic, and all of the azulene subunits become aromatic. Further oxidation to the dication **[2-diMes]** ^**2+**^ leads to a loss of aromaticity of two central azulene subunits and the core benzene ring.

**Figure 6:**
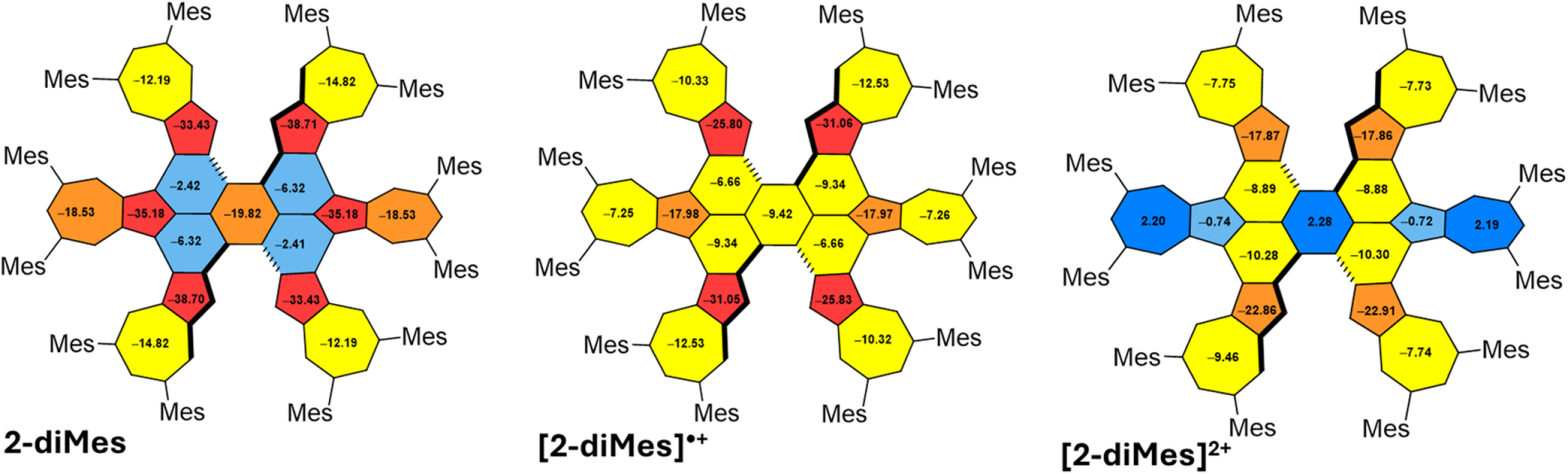
NICS­(1)_*zz*_ values of **2-diMes**, **[2-diMes]** ^**•+**^, and **[2-diMes]** ^**2+**^; (U)­B3LYP/6–31g­(d,p).

### Photoacoustic Spectroscopy (PAS) and Photothermal Deflection Spectroscopy (PDS)

Photoacoustic spectroscopy (PAS) was employed to investigate the photothermal conversion properties of **2-diMes**. In this technique, an intensity-modulated monochromatic beam illuminates the sample inside an airtight cell, where absorbed light is converted into heat via nonradiative relaxation. The obtained spectra, as shown in Figure 7a, confirm a relatively strong absorption extending into the NIR-II region. For **2-diMes**, the measurement demonstrates efficient light-to-heat energy conversion, reflected by the strong and well-defined photoacoustic response across the studied spectral range. To complement these results, photothermal deflection spectroscopy (PDS) was performed, allowing the investigation of both the thin-film sample and the DCM (dichloromethane) solution, where the primary solvent acted as the thermooptic medium. The measured spectra, as presented in Figure S22, indicate an absorption onset around 0.85 eV (1450 nm) with a distinct exponential tail, also known as the Urbach edge.^81,82^ The extracted Urbach energies (*E*_U_), corresponding to the energetic disorder of the material, amount to 49 meV for the thin film and 45 meV for the dichloromethane solution. The higher disorder parameter in the thin film may be attributed to aggregation phenomena or rigidification of molecules occurring during the thin-film deposition. Furthermore, the time-dependent measurement of the photoacoustic response under 980 nm excitation at an illumination power density of 222 W/cm^2^ was used to evaluate the photostability of the material (Figure 7b). The PA signal remained stable, preserving its initial intensity during 60 min of continuous irradiation, which confirms the photostability of **2-diMes**. Altogether, these results underline that **2-diMes** combines strong NIR-II absorption and low *E*_U_ with efficient and stable photothermal conversion, which makes them highly promising for organic electronic applications and theranostics.^83^

**Figure 7:**
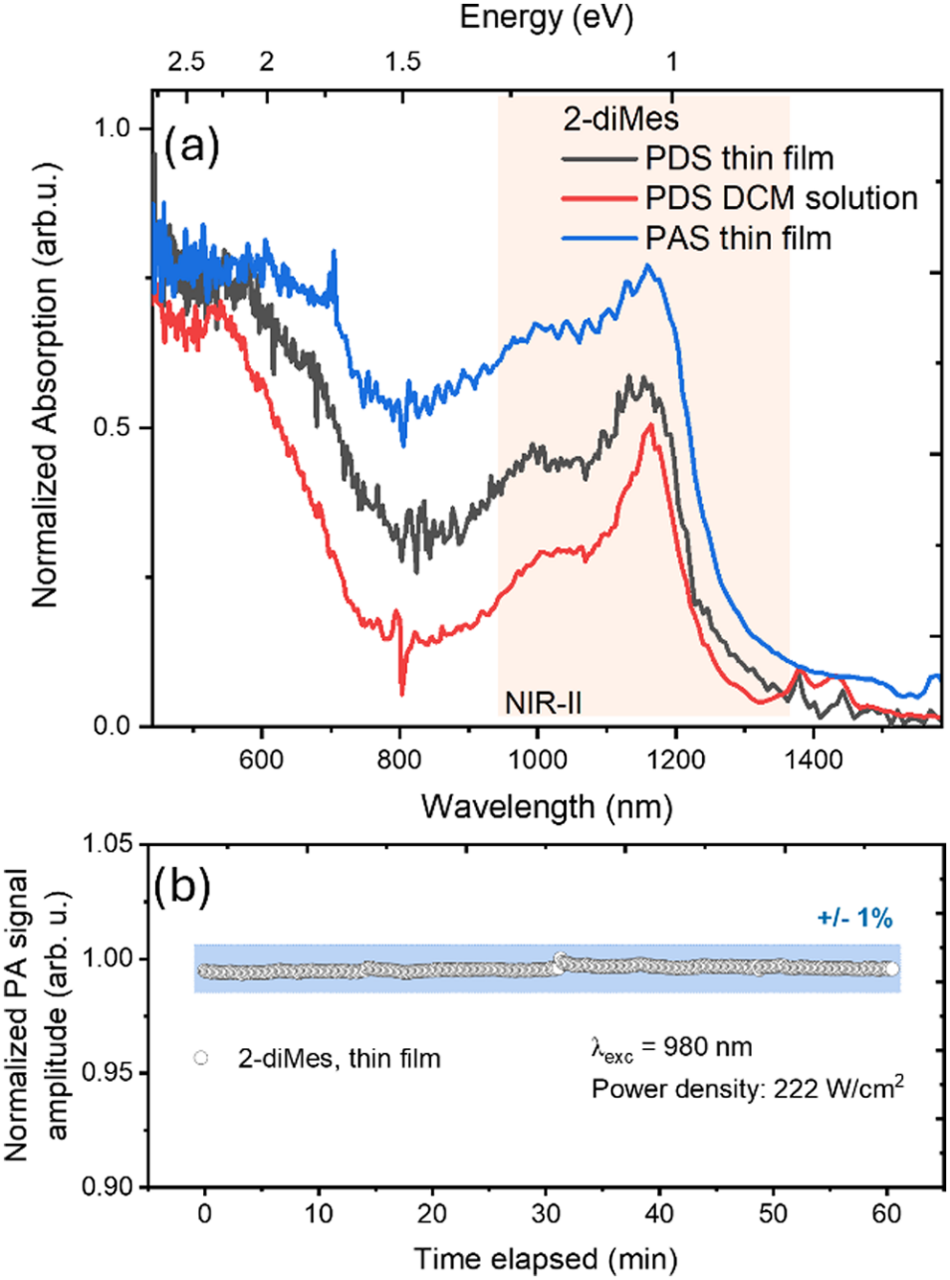
(a) Photothermal response spectra of the **2-diMes** thin film and DCM solution measured using photoacoustic spectroscopy (PAS) and photothermal deflection spectroscopy (PDS). (b) Photostability test performed on a thin film of **2-diMes** upon intense illumination in a highly absorbing spectral region.

### Cation Sensing

Finally, the receptor properties of **2-diMes** were investigated by using UV/vis/NIR spectroscopy. Owing to its highly π-conjugated structure, **2-diMes** is expected to engage in cation−π interactions with metal cations, especially focusing on alkali cations. Notably, the application of warped PAH molecules as receptors remains relatively rare and has largely focused on bowl-shaped sumanene derivatives for the detection of alkali metals, particularly with an emphasis on Cs^+^ sensing.^84^ In contrast, the use of bulk graphene for metal cation sensing has been extensively studied and well documented in the literature.^85–87^ Taking this into account, receptor properties of **2-diMes** were tested against s-block alkali metal cations, namely, lithium (Li^+^), sodium (Na^+^), potassium (K^+^), rubidium (Rb^+^), and cesium (Cs^+^). Due to the limited solubility of **2-diMes** in water, titrations were performed in THF/H_2_O = 1/1; *v*/*v*, solvent system. Full experimental details and data are presented in the Supporting Information.

For all cations, lowering of the absorbance of **2-diMes** was observed upon the addition of a cation (see the representative spectra for Cs^+^ titration in Figure 8a). It suggested that **2-diMes**, being a purely hydrocarbon molecule, indeed interacted with the tested cations by means of noncovalent, cation−π forces. The changes in the spectra were not the same, which was the result of the different affinity of the receptor for the given analyte. For most systems, a 2:1 stoichiometry of the formed complexes could be concluded (SI, Table S5). However, in the case of Li^+^ and Cs^+^, a 1:1 stoichiometry was also found to provide a reasonable fitting quality. All systems featured satisfactory binding parameters, with association constant (*K*_a_) values at the level of 1 × 10^4^ M^–1^. Notably, these values achieved for **2-diMes** are comparable to the reported receptors featuring the bowl-shaped motif, with sumanene-based Cs^+^ receptors as the leading example (*K*_a_ ranging from 3·10^4^ to 90·10^4^ M^–1^).^53^ It could be observed that the highest *K*_a_ value was found for the **2-diMes**-Na^+^ system, which is in good agreement with the trends of titration curves (SI, Figure S15). However, from the thermodynamic viewpoint, **2-diMes** tends to interact more strongly with bigger cations. This behavior may be attributed to the structure of **2-diMes**, where the warped π-scaffold, in combination with the mesityl substituents, forms a negatively charged pocket capable of binding positively charged cations (Figure 8b). The lowest association constant (*K*_a_) observed for Li^+^ can be explained by its strong solvation in water, which arises from its small ionic radius, the smallest among the tested series of alkali metal cations.^88^

**Figure 8:**
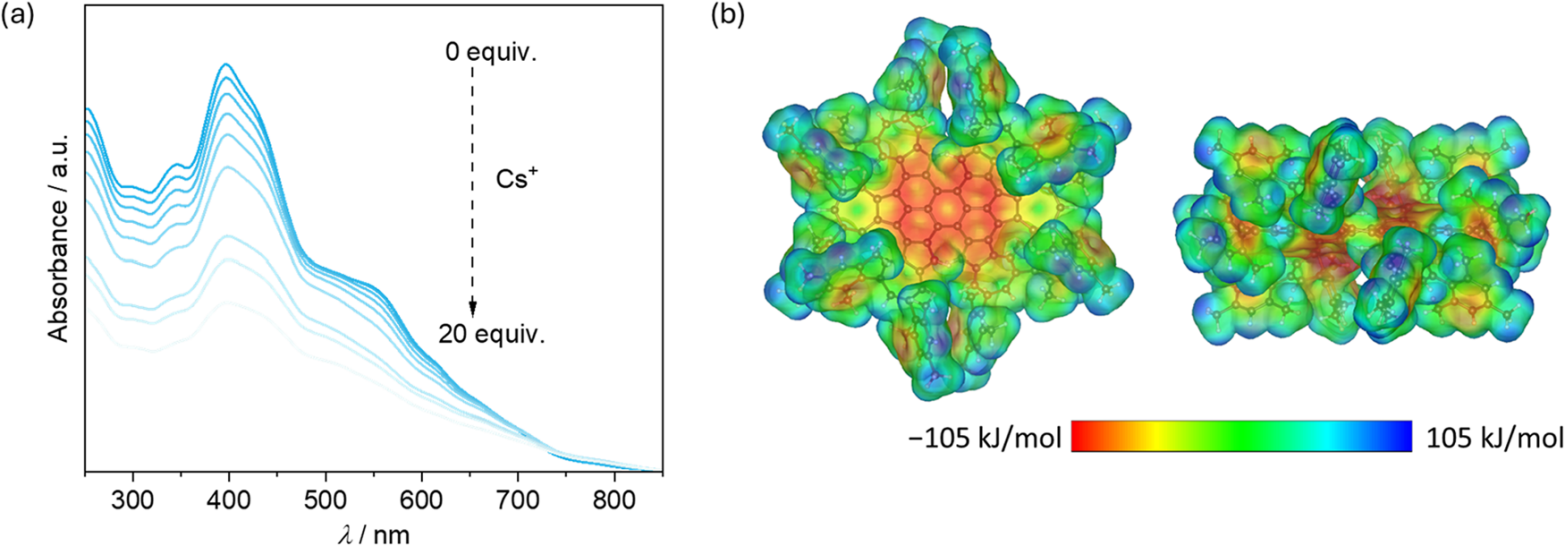
(a) UV/vis titration spectra of **2-diMes** with Cs^+^ as the representative example of an alkali cation (THF:H_2_O = 1/1 *v*/*v*, *c* = 2·10^–5^ M). (b) ESP plots of **2-diMes** (B3LYP/6–31G­(d,p), isovalue 0.004 au, top and side views).

## Conclusion

This work establishes Scholl oxidation as a powerful strategy for the construction of nonbenzenoid PAHs incorporating multiple azulene units. The resulting C_66_ PAH, **2-diMes**, represents the first fully π-conjugated hydrocarbon framework containing six azulene subunits. Its nonalternant topology engenders exceptional near-infrared optical properties, including one of the smallest HOMO–LUMO gaps reported for neutral closed-shell purely hydrocarbon nanographenes and pronounced absorption extending deep into the NIR-II region. Beyond fundamental electronic structure considerations, **2-diMes** functions as a receptor for s-block metal cations, introducing a new optical sensing paradigm within azulene-rich PAHs. Importantly, controlled oxidation of **2-diMes** affords stable radical cation and dication species that exhibit further red-shifted optical absorption extending into the mid-infrared region, underscoring the exceptional gap tunability accessible in this nonalternant π-conjugated scaffold. The small electronic gap and redox-accessible electronic states enable efficient and photostable photothermal conversion, as demonstrated by photoacoustic and photothermal deflection spectroscopies, positioning **2-diMes** as a rare example of a purely hydrocarbon NIR-II photoacoustic dye. Together, these results highlight nonalternant, azulene-embedded nanographenes as a promising and underexplored platform for the rational design of next-generation NIR-II dyes and redox-tunable functional organic materials. We anticipate that an extension of this synthetic strategy to larger and more complex architectures will further expand the scope of nonalternant hydrocarbons in optoelectronics and theranostic applications.

## Supplementary Material



## Notes

Experimental methods, synthetic protocols, electronic spectra, electrochemical data, details of the X-ray crystallographic analysis, details of PAS and PDS measurements, descriptions of quantum chemical calculations, titration experiments, and ^1^H and ^13^C NMR spectra (PDF)

## References

[1] Narita A., Wang X. Y., Feng X., Müllen K. (2015). New Advances
in Nanographene Chemistry. Chem. Soc. Rev..

[2] Liu Z., Fu S., Liu X., Narita A., Samorì P., Bonn M., Wang H. I. (2022). Small Size,
Big Impact: Recent Progress
in Bottom-Up Synthesized Nanographenes for Optoelectronic and Energy
Applications. Adv. Sci..

[3] Zahradník R. (1965). Electronic
Structure and Properties of Non-Alternant Hydrocarbons. Angew. Chem., Int. Ed..

[4] Anderson A. G., Steckler B. M. (1959). Azulene. VIII. A
Study of the Visible Absorption Spectra
and Dipole Moments of Some 1- and 1,3-Substituted Azulenes. J. Am. Chem. Soc..

[5] Michl J., Thulstrup E. W. (1976). Why Is Azulene Blue and Anthracene White? A Simple
MO Picture. Tetrahedron.

[6] Dunlop D., Ludvíková L., Banerjee A., Ottosson H., Slanina T. (2023). Excited-State (Anti)­Aromaticity
Explains Why Azulene
Disobeys Kasha’s Rule. J. Am. Chem. Soc..

[7] Pigulski B., Shoyama K., Würthner F. (2020). NIR-Absorbing π-Extended Azulene:
Non-Alternant Isomer of Terrylene Bisimide. Angew. Chem., Int. Ed..

[8] Hatakenaka R., Urabe K., Ueno S., Yamauchi M., Mizuhata Y., Yamada H., Mikata Y., Kamijo S., Tani F., Murafuji T. (2025). Doubly Linked Azulene
Dimer: A Novel Non-benzenoid
Isomer of Perylene. Chem. - Eur. J..

[9] Diaz-Andres A., Marín-Beloqui J., Wang J., Liu J., Casado J., Casanova D. (2023). Rational Design of Anti-Kasha Photoemission
from a
Biazulene Core Embedded in an Antiaromatic/Aromatic Hybrid. Chem. Sci..

[10] Huang F., Díaz-Fernández M., Marín-Beloqui J. M., Sun L., Chen Y., Liu S., Wang Y., Zheng H., Li S., Zhang C., You J., Casado J. (2025). Optimal Synergy between
Azulenes and Acenes in Azuacenes with 6–7-5 Ring Topology. J. Am. Chem. Soc..

[11] Yamamoto K., Ie Y., Tohnai N., Kakiuchi F., Aso Y. (2018). Antiaromatic Character
of Cycloheptatriene-Bis-Annelated Indenofluorene Framework Mainly
Originated from Heptafulvene Segment. Sci. Rep..

[12] Liu J., Mishra S., Pignedoli C. A., Passerone D., Urgel J. I., Fabrizio A., Lohr T. G., Ma J., Komber H., Baumgarten M., Corminboeuf C., Berger R., Ruffieux P., Müllen K., Fasel R., Feng X. (2019). Open-Shell Nonbenzenoid Nanographenes
Containing Two Pairs of Pentagonal and Heptagonal Rings. J. Am. Chem. Soc..

[13] Fei Y., Liu J. (2022). Synthesis of Defective
Nanographenes Containing Joined Pentagons
and Heptagons. Adv. Sci..

[14] Konishi A., Yasuda M. (2021). Breathing New Life
into Nonalternant Hydrocarbon Chemistry:
Syntheses and Properties of Polycyclic Hydrocarbons Containing Azulene,
Pentalene, and Heptalene Frameworks. Chem. Lett..

[15] Xin H., Hou B., Gao X. (2021). Azulene-Based
π-Functional Materials: Design,
Synthesis, and Applications. Acc. Chem. Res..

[16] Xin H., Gao X. (2017). Application
of Azulene in Constructing Organic Optoelectronic Materials:
New Tricks for an Old Dog. ChemPlusChem.

[17] Zhang Z., Pham H. D. M., Perepichka D. F., Khaliullin R. Z. (2024). Prediction
of Highly Stable 2D Carbon Allotropes Based on Azulenoid Kekulene. Nat. Commun..

[18] Wang Z., Zhou X. F., Zhang X., Zhu Q., Dong H., Zhao M., Oganov A. R. (2015). Phagraphene: A Low-Energy
Graphene
Allotrope Composed of 5–6-7 Carbon Rings with Distorted Dirac
Cones. Nano Lett..

[19] Fan Q., Martin-Jimenez D., Ebeling D., Krug C. K., Brechmann L., Kohlmeyer C., Hilt G., Hieringer W., Schirmeisen A., Gottfried J. M. (2019). Nanoribbons with Nonalternant Topology
from Fusion of Polyazulene: Carbon Allotropes beyond Graphene. J. Am. Chem. Soc..

[20] Li J., Li S., Ouyang T., Zhang C., Tang C., He C., Zhong J. (2021). Two-Dimensional
Carbon Allotropes and Nanoribbons Based on 2,6-Polyazulene
Chains: Stacking Stabilities and Electronic Properties. J. Phys. Chem. Lett..

[21] Li X., Wang Q., Jena P. (2017). ψ-Graphene:
A New Metallic
Allotrope of Planar Carbon with Potential Applications as Anode Materials
for Lithium-Ion Batteries. J. Phys. Chem. Lett..

[22] Duan C., Zhang J., Xiang J., Yang X., Gao X. (2022). Azulene-Embedded
[*n*]­Helicenes (*n* = 5, 6 and 7). Angew. Chem., Int. Ed..

[23] Ong A., Tao T., Jiang Q., Han Y., Ou Y., Huang K. W., Chi C. (2022). Azulene-Fused Acenes. Angew. Chem., Int. Ed..

[24] Ren P., Chen L., Sun C., Hua X., Luo N., Fan B., Chen P., Shao X., Zhang H. L., Liu Z. (2024). Linear Non-Benzenoid
Isomer of Acene Fusing Chrysene with Azulene Units. J. Chem. Phys. Lett..

[25] Wang S., Tang M., Wu L., Bian L., Jiang L., Liu J., Tang Z. B., Liang Y., Liu Z. (2022). Linear Nonalternant
Isomers of Acenes Fusing Multiple Azulene Units. Angew. Chem., Int. Ed..

[26] Lin Z., Wang C., Shiri F., Lin Z., Liu J. (2026). Diazulenopentalene:
Facile Synthesis of Linear Non-Alternant Molecular Carbons through
Pt-Mediated Rearrangement. J. Am. Chem. Soc..

[27] Jiang Q., Wang T., Han Y., Wei H., Deng Y., Geng Y., Chi C. (2025). Bis­(azuleno)­pentalenes with Six Consecutive
Odd-Membered Rings. Angew. Chem., Int. Ed..

[28] Liu S., Díaz-Fernández M., Zhang M., Huang F., Chen Y., Yang Y., Beloqui J. M. M., Lan J., You J., Casado J., Zhang C. (2025). Azuperylene: The Non-Alternant Isomer
of Perylene. Angew. Chem., Int. Ed..

[29] Ma J., Fu Y., Dmitrieva E., Liu F., Komber H., Hennersdorf F., Popov A. A., Weigand J. J., Liu J., Feng X. (2020). Helical Nanographenes
Containing an Azulene Unit: Synthesis, Crystal Structures, and Properties. Angew. Chem., Int. Ed..

[30] Han Y., Xue Z., Li G., Gu Y., Ni Y., Dong S., Chi C. (2020). Formation of Azulene-Embedded
Nanographene: Naphthalene to Azulene
Rearrangement During the Scholl Reaction. Angew.
Chem., Int. Ed..

[31] Liu W., Zhang H., Zhang T., Lu Z., Wagenhäuser Y., Würthner F., Zhu C. (2026). A Monkey-Saddle-Shaped Nanographene
Embedding a Dicyclohepta­[*cd*, *fg*]-*as*-Indacene Core and Two Additional Heptagons, and Its Paddle-Wheel
Co-Assembly With Fullerenes. Angew. Chem., Int.
Ed..

[32] Luo H., Liu J. (2024). Non-Alternant Nanographenes Bearing N-Doped Non-Hexagonal Pairs:
Synthesis, Structural Analysis and Photophysical Properties. Angew. Chem., Int. Ed..

[33] Qiu S., Valdivia A. C., Zhuang W., Hung F. F., Che C. M., Casado J., Liu J. (2024). Nonalternant
Nanographenes Containing
N-Centered Cyclopenta­[*ef*]­heptalene and Aza[7]­Helicene
Units. J. Am. Chem. Soc..

[34] Qiu S., Chen K., Deng Z., Jin X., Li Z., Liu G., Zhang L., Jiang W., Chen T. T., Liu J., Wang Z. (2025). N-Doped Nonalternant
Molecular Bowl/Saddle Hybrids. Angew. Chem.,
Int. Ed..

[35] Pigulski B. (2025). Recent Advances
and Future Challenges in the Bottom-up Synthesis of Azulene-Embedded
Nanographenes. Beilstein J. Org. Chem..

[36] Mathey P., Sobczak Q., Darvish A., Morin J.-F. (2024). Synthesis of an
Azulene-Containing Graphene Nanoribbon. Chem.
Commun..

[37] Hou I. C. Y., Sun Q., Eimre K., Di Giovannantonio M., Urgel J. I., Ruffieux P., Narita A., Fasel R., Müllen K. (2020). On-Surface Synthesis of Unsaturated Carbon Nanostructures
with Regularly Fused Pentagon-Heptagon Pairs. J. Am. Chem. Soc..

[38] Koide T., Takesue M., Murafuji T., Satomi K., Suzuki Y., Kawamata J., Terai K., Suzuki M., Yamada H., Shiota Y., Yoshizawa K., Tani F. (2017). An Azulene-Fused Tetracene
Diimide with a Small HOMO–LUMO Gap. ChemPlusChem.

[39] Sasaki Y., Takase M., Okujima T., Mori S., Uno H. (2019). Synthesis
and Redox Properties of Pyrrole- and Azulene-Fused Azacoronene. Org. Lett..

[40] Wang C., Deng Z., Phillips D. L., Liu J. (2023). Extension of Non-Alternant
Nanographenes Containing Nitrogen-Doped Stone-Thrower-Wales Defects. Angew. Chem., Int. Ed..

[41] Wang C., Hu C., Wang W., Yang J., Liu J. (2025). Toward the Synthesis
of Pentaheptite Substructure: The Cyclopenta­[*ef*]­heptalene
to Phenanthrene Rearrangement. CCS Chem..

[42] Kirschbaum T., Rominger F., Mastalerz M. (2023). Synthesis
of a Benzotrisazulene via
Trioxobenzotrisazulene. Chem. - Eur. J..

[43] Liang Y., Wang S., Tang M., Wu L., Bian L., Jiang L., Tang Z. B., Liu J., Guan A., Liu Z. (2023). Cascade Synthesis of Benzotriazulene
with Three Embedded Azulene
Units and Large Stokes Shifts. Angew. Chem.,
Int. Ed..

[44] Fabian J., Nakazumi H., Matsuoka M. (1992). Near-Infrared Absorbing Dyes. Chem. Rev..

[45] Kejík Z., Hajduch J., Abramenko N., Vellieux F., Veselá K., Fialová J. L., Petrláková K., Kučnirová K., Kaplánek R., Tatar A., Skaličková M., Masařík M., Babula P., Dytrych P., Hoskovec D., Martásek P., Jakubek M. (2024). Cyanine Dyes in the
Mitochondria-Targeting Photodynamic and Photothermal Therapy. Commun. Chem..

[46] Tian M., Tatsuura S., Furuki M., Sato Y., Iwasa I., Pu L. S. (2003). Discovery of Novel Dyes with Absorption Maxima at 1.1 μm. J. Am. Chem. Soc..

[47] Simard T. P., Yu J. H., Zebrowski-Young J. M., Haley N. F., Detty M. R. (2000). Soluble,
Infrared-Absorbing Croconate Dyes from 2,6-Di-*tert*-butyl-4-methylchalcogenopyrylium Salts. J.
Org. Chem..

[48] Gong Q., Shao J., Li W., Guo X., Ling S., Wu Y., Wei Y., Xu X., Jiang X., Jiao L., Hao E. (2025). Fully Conjugated Thiophene-Fused Oligo-BODIPYs: A Class of Intensely
Near-Infrared Absorbing, Arc-Shaped Materials with up to 31 Linearly-Fused
Rings. J. Am. Chem. Soc..

[49] Jiao L., Zou Y., Fan W., Han Y., Zhou Q., Shao J., Wu J. (2025). Aggregation-Free, Highly
Soluble CN-Terminated Dicyclopentadiene-Fused
Rylenes. J. Am. Chem. Soc..

[50] Zeng W., Phan H., Herng T. S., Gopalakrishna T. Y., Aratani N., Zeng Z., Yamada H., Ding J., Wu J. (2017). Rylene Ribbons with Unusual Diradical
Character. Chem.

[51] Yuan Z., Lee S. L., Chen L., Li C., Mali K. S., De Feyter S., Müllen K. (2013). Processable
Rylene Diimide Dyes up
to 4 nm in Length: Synthesis and STM Visualization. Chem. - Eur. J..

[52] Chen Q., Lodi A., Zhang H., Gee A., Wang H. I., Kong F., Clarke M., Edmondson M., Hart J., O’Shea J. N., Stawski W., Baugh J., Narita A., Saywell A., Bonn M., Müllen K., Bogani L., Anderson H. L. (2024). Porphyrin-Fused Graphene Nanoribbons. Nat. Chem..

[53] Muranaka A., Yonehara M., Uchiyama M. (2010). Azulenocyanine:
A New Family of Phthalocyanines
with Intense near-IR Absorption. J. Am. Chem.
Soc..

[54] Wang K., Liu P., Zhang F., Xu L., Zhou M., Nakai A., Kato K., Furukawa K., Tanaka T., Osuka A., Song J. (2021). A Robust Porphyrin-Stabilized Triplet Carbon Diradical. Angew. Chem., Int. Ed..

[55] Luo J., Xu X., Mao R., Miao Q. (2012). Curved Polycyclic Aromatic Molecules
That Are π-Isoelectronic to Hexabenzocoronene. J. Am. Chem. Soc..

[56] Steiner A. K., Sharapa D. I., Troyanov S. I., Nuss J., Amsharov K. (2021). Carbon Origami
via an Alumina-Assisted Cyclodehydrofluorination Strategy. Chem. - Eur. J..

[57] Herwig P., Kayser C. W., Müllen K., Spiess H. W. (1996). Columnar Mesophases
of Alkylated Hexa-Peri-Hexabenzocoronenes with Remarkably Large Phase
Widths. Adv. Mater..

[58] Takase M., Enkelmann V., Sebastiani D., Baumgarten M., Müllen K. (2007). Annularly
Fused Hexapyrrolohexaazacoronenes: An Extended
π System with Multiple Interior Nitrogen Atoms Displays Stable
Oxidation States. Angew. Chem., Int. Ed..

[59] Borkowski M., Różyło L., Szafert S., Pigulski B. (2026). Revisiting
Hexakis­(azulen-2-yl)­benzenes: A New Synthetic Approach Using Suzuki
Cross-Coupling. Can. J. Chem..

[60] Ito S., Nomura A., Morita N., Kabuto C., Kobayashi H., Maejima S., Fujimori K., Yasunami M. (2002). Synthesis and Two-Electron
Redox Behavior of Diazuleno­[2,1-*a*:1,2-*c*]­Naphthalenes. J. Org. Chem..

[61] Huang Y., Brown M. K. (2019). Synthesis of Bisheteroarylalkanes
by Heteroarylboration:
Development and Application of a Pyridylidene–Copper Complex. Angew. Chem., Int. Ed..

[62] Ziegler K., Hafner K. (1955). Eine Rationelle Synthese Des Azulens. Angew. Chem..

[63] Fujinaga M., Murafuji T., Kurotobi K., Sugihara Y. (2009). Polyborylation
of Azulenes. Tetrahedron.

[64] Kurotobi K., Miyauchi M., Takakura K., Murafuji T., Sugihara Y. (2003). Direct Introduction
of a Boryl Substituent into the 2-Position of Azulene: Application
of the Miyaura and Smith Methods to Azulene. Eur. J. Org. Chem..

[65] Biesaga J., Szafert S., Pigulski B. (2024). 1,2,3-Triarylazulenes as Precursors
of Azulene-Embedded Polycyclic Aromatic Hydrocarbons. Org. Chem. Front..

[66] Zhang Y., Pun S. H., Miao Q. (2022). The Scholl Reaction as a Powerful
Tool for Synthesis of Curved Polycyclic Aromatics. Chem. Rev..

[67] Grzybowski M., Sadowski B., Butenschön H., Gryko D. T. (2020). Synthetic Applications
of Oxidative Aromatic Coupling—From Biphenols to Nanographenes. Angew. Chem., Int. Ed..

[68] Pun S. H., Wen E. C. H., Xia Z., Chen H., Fischer F. R., Miao Q. (2023). Reactivity, Regioselectivity,
and Synthetic Application of 2-Pyrenyl
Units in Scholl Reactions. CCS Chem..

[69] Moshniaha L., Żyła-Karwowska M., Chmielewski P. J., Lis T., Cybińska J., Gońka E., Oschwald J., Drewello T., Rivero S. M., Casado J., Stȩpień M. (2020). Aromatic Nanosandwich Obtained by
σ-Dimerization of a Nanographenoid π-Radical. J. Am. Chem. Soc..

[70] Li Z., Zhu K., Liang J., Gong H. (2025). Covalent Bond Regulation in Nanographene:
En Route to Precise Aggregation State Modification. Aggregate.

[71] Kawasumi K., Zhang Q., Segawa Y., Scott L. T., Itami K. (2013). A Grossly
Warped Nanographene and the Consequences of Multiple Odd-Membered-Ring
Defects. Nat. Chem..

[72] Kato K., Segawa Y., Scott L. T., Itami K. (2018). A Quintuple [6]­Helicene
with a Corannulene Core as a *C*
_5_-Symmetric
Propeller-Shaped π-System. Angew. Chem.,
Int. Ed..

[73] Kruszewski J., Krygowski T. M. (1972). Definition of Aromaticity Basing on the Harmonic Oscillator
Model. Tetrahedron Lett..

[74] Tan Y. Z., Yang B., Parvez K., Narita A., Osella S., Beljonne D., Feng X., Müllen K. (2013). Atomically
Precise Edge Chlorination of Nanographenes and Its Application in
Graphene Nanoribbons. Nat. Commun..

[75] Zhu Y., Guo X., Li Y., Wang J. (2019). Fusing of Seven HBCs toward a Green
Nanographene Propeller. J. Am. Chem. Soc..

[76] Seguy I., Jolinat P., Destruel P., Mamy R., Allouchi H., Courseille C., Cotrait M., Bock H. (2001). Crystal and Electronic
Structure of a Fluorescent Columnar Liquid Crystalline Electron Transport
Material. ChemPhysChem.

[77] Nagase M., Kato K., Yagi A., Segawa Y., Itami K. (2020). Six-Fold C-H
Borylation of Hexa-Peri-Hexabenzocoronene. Beilstein
J. Org. Chem..

[78] Martin M.
M., Hampel F., Jux N. (2020). A Hexabenzocoronene-Based Helical
Nanographene. Chem. - Eur. J..

[79] Sohlberg K., Foster M. E. (2020). What’s the
Gap? A Possible Strategy for Advancing
Theory, and an Appeal for Experimental Structure Data to Drive That
Advance. RSC Adv..

[80] Chen Z., Wannere C. S., Corminboeuf C., Puchta R., Von P., Schleyer R. (2005). Nucleus-Independent
Chemical Shifts (NICS) as an Aromaticity
Criterion. Chem. Rev..

[81] Ugur E., Ledinský M., Allen T. G., Holovský J., Vlk A., De Wolf S. (2022). Life on the Urbach Edge. J. Phys.
Chem. Lett..

[82] Urbach F. (1953). The Long-Wavelength
Edge of Photographic and of the Electronic of Solids. Phys. Rev..

[83] Zhu J. H., Luo L., Gu M., Chen Y., Wang L., Li M., Chen X., Peng X. (2025). Second Near-Infrared Region Chemiluminescent
Theranostics: Opportunities and Challenges. CCS Chem..

[84] Kasprzak A. (2024). Supramolecular
Chemistry of Sumanene. Angew. Chem., Int. Ed..

[85] Olsson E., Chai G., Dove M., Cai Q. (2019). Adsorption and Migration
of Alkali Metals (Li, Na, and K) on Pristine and Defective Graphene
Surfaces. Nanoscale.

[86] Lin Y. C., Matsumoto R., Liu Q., Solís-Fernández P., Siao M. D., Chiu P. W., Ago H., Suenaga K. (2024). Alkali Metal
Bilayer Intercalation in Graphene. Nat. Commun..

[87] Malhotra M., Puglia M., Kalluri A., Chowdhury D., Kumar C. V. (2022). Adsorption of Metal Ions on Graphene Sheet for Applications
in Environmental Sensing and Wastewater Treatment. Sens. Actuators Rep..

[88] Mähler J., Persson I. (2012). A Study of the Hydration of the Alkali
Metal Ions in
Aqueous Solution. Inorg. Chem..

