# Supplementary material for: A C66 Polycyclic Aromatic Hydrocarbon with Six Azulene Units and NIR-II Absorption: Toward Azulene-Based Carbon Allotropes
Source: J Am Chem Soc. 2026 Apr 15;148(16):17469–80. doi: 10.1021/jacs.6c04336 (PMC13298906; doi:10.1021/jacs.6c04336)
Supplement: Supplementary file 1 [file ja6c04336_si_001.pdf]

A C_{66} Polycyclic Aromatic Hydrocarbon with Six Azulene Units and NIR-II Absorption: Toward Azulene-Based Carbon Allotropes

Maksymilian Borkowski,[a] Ada Drwęcka,[b] Szymon J. Zelewski,[b] Artur Kasprzak,[c] Sławomir Szafert,[a] Bartłomiej Pigulski[a]*

[a]Faculty of Chemistry, University of Wrocław, Joliot Curie 14, 50-383 Wrocław, Poland

[b]Department of Experimental Physics, Faculty of Fundamental Problems of Technology, Wrocław University of Science and Technology, 50-370 Wrocław, Poland

[c]Faculty of Chemistry, Warsaw University of Technology, Noakowskiego 3, 00-664 Warsaw, Poland

E-mail: bartlomiej.pigulski@uwr.edu.pl

Contents

Experimental Section ... 3

Optimization of oxidation conditions ... 5

Alternative synthetic approaches to 1-diMes ... 6

Synthesis ... 7

4-diMes, 1-butyl-3,5-dimesitylpyridin-1-ium bromide ... 7

5-diMes, 5,7-dimesitylazulene ... 7

6-diMes, 2-(5,7-dimesitylazulen-2-yl)-4,4,5,5-tetramethyl-1,3,2-dioxaborolane ... 8

7-diMes, 1,2,3,4,5,6-hexakis(5,7-dimesitylazulen-2-yl)benzene ... 9

2-diMes, 2,4,9,11,14,16,19,21,26,28,31,33-dodecamesitylhexaazuleno[2,1-*a*:1',2',3'-*cd*:1'',2''-*f*:2''',1'''-*j*:1'''',2'''',3''''-*lm*:1''''',2'''''-*o*]perylene ... 10

X-ray single crystal diffraction ... 11

Cations sensing experiments ... 13

Photoacoustic (PA) and photothermal deflection spectroscopy (PDS) ... 21

NMR Spectra ... 23

4-diMes, 1-butyl-3,5-dimesitylpyridin-1-ium bromide ... 23

5-diMes, 5,7-dimesitylazulene ... 24

6-diMes, 2-(5,7-dimesitylazulen-2-yl)-4,4,5,5-tetramethyl-1,3,2-dioxaborolane ... 25

7-diMes, 1,2,3,4,5,6-hexakis(5,7-dimesitylazulen-2-yl)benzene ... 26

2-diMes, 2,4,9,11,14,16,19,21,26,28,31,33-dodecamesitylhexaazuleno[2,1-*a*:1',2',3'-*cd*:1'',2''-*f*:2''',1'''-*j*:1'''',2'''',3''''-*lm*:1''''',2'''''-*o*]perylene ... 27

Electrochemistry ... 32

HRMS spectra ... 33

UV/Vis/NIR and fluorescence spectra ... 34

Spectroelectrochemistry ... 36

DFT calculations ... 37

DFT-optimized cartesian coordinates ... 44

References ... 64

Experimental Section

Synthesis: Sensitive reactions were conducted under N_2 using standard Schlenk techniques. Glassware was dried at 120 °C. Solvents for synthesis were treated as follows: 1,4-dioxane (POCH): mBraun solvent purification system, CH_2Cl_2 (Aldrich): mBraun solvent purification system, $MeNO_2$ (POCH) dried over molecular sieves, THF (POCH) purified using mBraun solvent purification system, and CH_2Cl_2 (ChemPur) were used without further purification.

1-Bromobutane (POCH, 98%), $B_2(pin)_2$ (Alfa Aesar, 98%), Cs_2CO_3 (Sigma Aldrich, 99%) 4,4′-dimethyl-2,2′-dipyridyl (AmBeed, 99.86%), hexabromobenzene (Aldrich, 98%), were used as received. Catalysts were purified by recrystallization under N_2: Pd(dppf)Cl_2·CH_2Cl_2 (Aldrich) from CH_2Cl_2/*n*-hexane (1/1) mixture, [Ir(cod)(OMe)]$_2$ (Aldrich, 98%) from CH_2Cl_2/MeOH (1/1, *v*/*v*) mixture.

Following compound was prepared according to the literature methods: 3,5-dimesitylpyridine (**3-diMes**).[1]

Chromatography: *Column chromatography*: standard glass columns and silica gel 60 (0.040-0.063 mm or 0.063-0.200 mm, Merck Millipore). Solvents for column chromatography were used without additional purification.

SEC (*Size exclusion chromatography*): Glass columns, BioBeads S-X3 stationary phase and CH_2Cl_2 or toluene as eluents.

GPC (*Gel permeation chromatography*): JAI LaboACE LC-7080 recycling preparative system with JAIGEL-2HR (20ϕx600) and JAIGEL-2.5HR (20ϕx600) column using $CHCl_3$ stabilized with EtOH as an eluent.

NMR Spectroscopy: ^{1}H and ^{13}C NMR spectra were recorded with a Bruker Avance 500 MHz and Avance III 600 MHz spectrometers. For all the ^{1}H NMR spectra, the chemical shifts are given in ppm relative to the solvent residual peaks ($CDCl_3$, ^{1}H: 7.24 ppm, ^{13}C: 77.16 ppm; CD_2Cl_2, ^{1}H: 5.32 ppm, C_6D_6, ^{1}H: 7.16 ppm, ^{13}C: 126.16 ppm). Coupling constants are given in Hz.

High Resolution Mass Spectrometry: HRMS spectra were recorded as follows: *ESI-HRMS*: Bruker qTOF compact, *APCI-HRMS*: Bruker qTOF compact.

UV/Vis/NIR Spectroscopy: Measurements of CH_2Cl_2 solutions (c~10^{-5} M) were carried out in 10 mm or 1 mm quartz cuvettes using two-beam JASCO V-770 spectrophotometer.

Spectroelectrochemistry: Measurements were carried out using 0.1 M tetrabutylammonium hexafluorophosphate CH_2Cl_2 solutions (c~10^{-4} M of **2-diMes**) in 2 mm quartz cuvettes using two-beam JASCO V-770 spectrophotometer and Metrohm Autolab/PGSTAT302N potentiostat/galvanostat. Platinum grid working electrode, platinum counter, and Ag/AgCl chloride reference electrode were used in the experimental setup.

Fluorescence: The photoluminescence measurement was performed using a 405 nm laser as an excitation source, mechanically modulated at 30 Hz, with a power density of 380 W/cm^2 for CH_2Cl_2 solution of **2-diMes**. The emitted light was analyzed with a grating monochromator, detected in single-channel configuration with a germanium-based photodetector (Thorlabs DET50B), and the signal was measured by a lock-in amplifier

Electrochemistry: Cyclic voltammetry (CV) and differential pulse voltammetry (DPV) measurements were carried out in CH_2Cl_2 (dried and degassed from mBraun solvent purification system) using a Metrohm Autolab/PGSTAT302N potentiostat/galvanostat in a glass cell under a N_2 atmosphere at room temperature. A platinum disk working electrode, a platinum wire auxiliary electrode and an Ag/AgCl reference electrode were used for all measurements. Tetrabutylammonium hexafluorophosphate (AmBeed) was used as a supporting electrolyte (0.1 M solution). CV: scan rate was 0.050 V s^{-1}. DPV: step size of 0.005 V, a modulation amplitude of 0.025 V, a modulation time of 0.5 s and an interval time of 1 s. All potentials were calibrated to the ferrocenium/ferrocene (Fc^+/Fc) redox couple.

Theoretical methods: Gaussian 16 software was used for density functional theory (DFT) and time-dependent density functional theory (TD-DFT) calculations.[2] B3LYP functional[3,4] and 6-31g(d,p) basis set were applied for neutral structure optimization, solvent model SCRF=(Solvent=Dichloromethane) was applied. Optimized ground-state geometries were examined by frequency analysis to possess no negative frequency. (U)B3LYP functionals and 6-31g(d,p) basis sets were applied for TD-DFT calculations of UV/Vis/NIR spectra and NICS(1)$_{zz}$ values. Multiwfn software was used for analysis of the electronic transitions[5] and py.Aroma 4 for NICS calculations.[6] IQmol[7] and VESTA[8] were use for visualization of theoretical calculations.

Optimization of oxidation conditions

Table S1. Attempts to oxidation of **7-diMes** (analysed using ^{1}H NMR and ESI-HRMS or APCI-HRMS).

Entry	Oxidant/Reagents	Eq.	Solvent	Temp.	Time	Outcome
**1**	$[(4\text{-}BrC_6H_4)_3N]SbCl_6$	12	CH_2Cl_2	−50°C	10 min	partial oxidation (mixture)
**2**	$AlCl_3$, $SnCl_4$	12, 12	CH_2Cl_2	0°C	10 min	partial decomposition
**3**	$AlCl_3$, $CuCl_2$	12, 12	CH_2Cl_2	0°C	5 min	partial decomposition
**4**	$AlCl_3$	12	CS_2	−80°°C	5 min	no changes
**5**	Cu	12	no solvent, KCl + strong stirring	RT	14 days	no changes
**6**	$MoCl_5$	12	CH_2Cl_2	−80°C	5 min	mostly unreacted substrate, partial oxidation (mixture)
**7**	$MoCl_5$	12	CH_2Cl_2	−80°C	60 min	mostly unreacted substrate, partial oxidation (mixture)
**8**	$MoCl_5$	12	CH_2Cl_2	0°C	60 min	unreacted substrate, partial oxidation (mixture)
**9**	$MoCl_5$	12	CH_2Cl_2	−80°C to RT	60 min	substrate and partial oxidation (mixture)
**10**	$MoCl_5$	12	CH_2Cl_2	0°C to RT	24h	decomposition
**11**	$Cu(OTf)_2$	12	CH_2Cl_2	0°C	90 min	mostly unreacted substrate and partial oxidation (mixture)
**12**	$Cu(OTf)_2$	12	CH_2Cl_2	0°C	3h	partial oxidation (mixture)
**13**	$Cu(OTf)_2$	12	CH_2Cl_2	0°C to RT	24h	partial oxidation (mixture)
**14**	$Cu(OTf)_2$, $AlCl_3$	12	CH_2Cl_2	0°C	3h	partial oxidation (mixture)
**15**	$Cu(OTf)_2$, $AlCl_3$	12	CH_2Cl_2	0°C to RT	24h	partial oxidation (mixture)
**16**	$Cu(OTf)_2$	12	CH_2Cl_2	RT	24h	decomposition
**17**	$Cu(OTf)_2$	12	CH_2Cl_2	reflux	70 min	decomposition
**18**	$Cu(OTf)_2$	12	CH_2Cl_2	5°C	5 days	decomposition
**19**	$FeCl_3$, K_2CO_3	30, 30	CH_2Cl_2:CH_3NO_2 10:1	−80°C	60 min	partial oxidation (mixture)
**20**	$FeCl_3$, K_2CO_3	30, 30	CH_2Cl_2:CH_3NO_2 10:1	−80°C	19h	decomposition
**21**	$FeCl_3$, K_2CO_3	30, 30	CH_2Cl_2:CH_3NO_2 10:1	−30°C	72h	decomposition
**22**	NCS	5	$CHCl_3$	RT	1 h	decomposition
**23**	NBS	5	$CHCl_3$	RT	1 h	decomposition
**24**	NIS	5	CH_2Cl_2	RT	1 h	decomposition
**25**	PIDA	10	CH_2Cl_2	−80°C to RT	1 h	decomposition
**26**	DDQ, $MeSO_3H$	6	CH_2Cl_2	0°C	5 min	decomposition
**27**	DDQ	6	CH_2Cl_2	0°C	5 min	pure **2-diMes**
**28**	DDQ	6	CH_2Cl_2	−80°C	30 min	**2-diMes** and trace amounts of less oxidized products

Table S2. Attempts to oxidation of **2-diMes** (analysed using ^{1}H NMR and ESI-HRMS or APCI-HRMS).

Entry	Oxidant/Reagents	Eq.	Solvent	Temp.	Time	Outcome
**1**	DDQ	2	CH_2Cl_2	0°C	30 min	no changes
**2**	DDQ +1 drop of $MeSO_3H$	2	CH_2Cl_2	RT	5 min	decomposition
**3**	DDQ	2	CH_2Cl_2	RT	5 min	no changes
**4**	DDQ	2	CH_2Cl_2	RT	18h	decomposition

Alternative synthetic approaches to 1-diMes

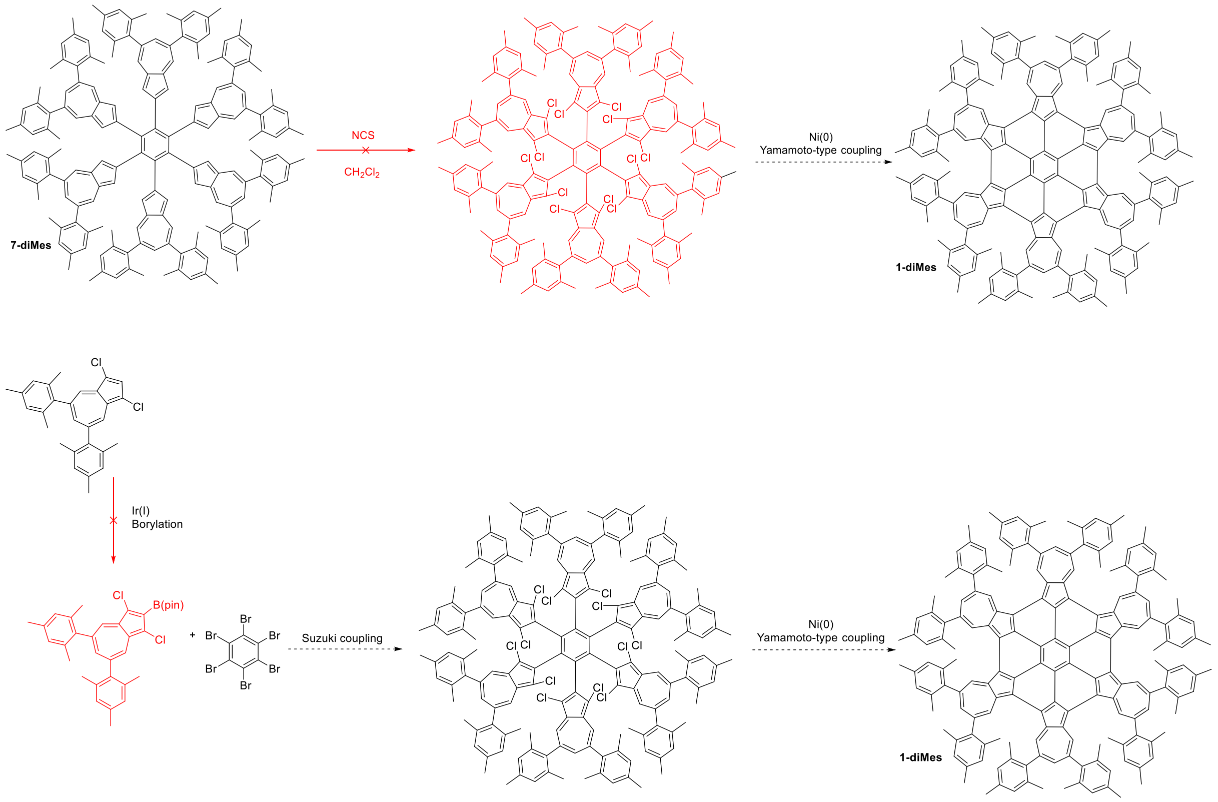

Figure S1. Tested alternative synthetic approaches to **1-diMes**.

Synthesis

4-diMes, 1-butyl-3,5-dimesitylpyridin-1-ium bromide

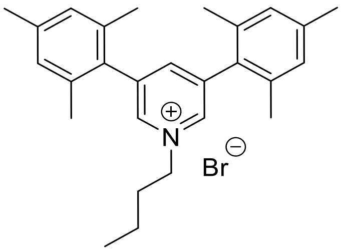

3,5-Dimesitylpyridine[1] (**3-diMes**, 2.00 g, 6.34 mmol) was placed in 20 mL vial and next 1-bromobutane (2.72 mL, 25.36 mmol) was added. The reaction mixture was stirred at 80°C for 17 h. Next solvent was removed under reduced pressure and precipitate was washed with hexane. Finally, product **4-diMes** was dried under reduced pressure giving 2.54 g (5.61 mmol) of white solid. Yield: 88%.

^{1}H NMR (500 MHz, $CDCl_3$) δ 9.01 (d, *J* = 1.5 Hz, 2H), 7.97 (t, *J* = 1.5 Hz, 1H), 7.00 (s, 4H), 5.35 (t, *J* = 7.2 Hz, 2H), 2.33 (s, 6H), 2.12 – 2.04 (m, 15H), 1.49 – 1.41 (m, 2H), 0.99 (t, *J* = 7.4 Hz, 3H).
^{13}C NMR (126 MHz, $CDCl_3$) δ 147.7, 143.2, 142.1, 140.0, 135.7, 130.1, 129.3, 62.6, 34.3, 21.2, 21.2, 19.4, 13.7.
HRMS-ESI: *m/z* calculated for $C_{27}H_{34}N$ (M-Br^-): 372.2686, measured: 372.2666.

5-diMes, 5,7-dimesitylazulene

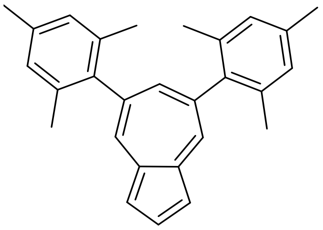

NaH (60% in mineral oil, 339 mg, 8.49 mmol) was placed in Schlenk flask and suspended in 10 mL of dry DMF. The reaction mixture was cooled down to 0°C and next freshly distilled cyclopentadiene was added dropwise under N_2. Next, 1-butyl-3,5-dimesitylpyridin-1-ium bromide (**4-diMes**, 2.56 g, 5.66 mmol) was used and the reaction mixture was refluxed for 3 h. The reaction mixture was cooled down to room temperature and quenched with 50 mL of water. The product was extracted using hexane (3 x 50 mL), the combined organic layer was dried using $MgSO_4$ and the solvent was removed under reduced pressure. Crude product was

purified using silica gel chromatography (petroleum ether) yielding 1.43 g of blue solid (3.92 mmol). Yield: 69%

^{1}H NMR (500 MHz, $CDCl_3$) δ 8.22 (d, *J* = 1.6 Hz, 2H), 7.94 (t, *J* = 3.7 Hz, 1H), 7.35 (d, *J* = 3.7 Hz, 2H), 7.30 (t, *J* = 1.4 Hz, 1H), 6.95 (s, 4H), 2.33 (s, 6H), 2.08 (s, 12H).
^{13}C NMR (126 MHz, $CDCl_3$) δ 142.0, 140.9, 139.8, 138.1, 137.6, 136.8, 135.8, 135.1, 128.3, 117.8, 21.2, 21.1.
HRMS-ESI: *m/z* calculated for $C_{28}H_{29}$ (M+H$^+$): 365.2264, measured: 365.2228.

6-diMes, 2-(5,7-dimesitylazulen-2-yl)-4,4,5,5-tetramethyl-1,3,2-dioxaborolane

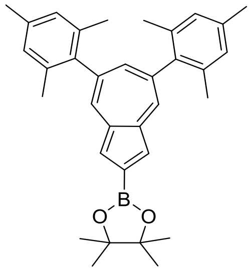

5,7-Dimesitylazulene (**5-diMes**, 1g, 2.74 mmol), $[Ir(cod)OMe)]_2$ (91 mg, 0.137 mmol), 2,2'-dmbpy (50 mg, 0.274 mmol), $B_2(pin)_2$ (766 mg, 3.02 mmol) were placed in Schlenk flask and next 20 mL of dry THF was added. The reaction mixture was heated overnight at 80°C under N_2. Next, the solvent was removed under reduced pressure, and the product was purified using a silica gel column (eluent: hexane/CH_2Cl_2, *v/v*, 1/0 to 0/1). Finally, 5,7-dimesitylazulene **5-diMes** was isolated as blue solid in 89% yield (1.2 g, 2.45 mmol).
^{1}H NMR (500 MHz, $CDCl_3$) δ 8.19 (d, *J* = 1.6 Hz, 2H), 7.69 (s, 2H), 7.25 (t, *J* = 1.6 Hz, 1H), 6.92 (s, 4H), 2.30 (s, 6H), 2.05 (s, 12H), 1.38 (s, 12H).
^{13}C NMR (151 MHz, $CDCl_3$) δ 142.2, 141.7, 140.1, 139.4, 136.8, 135.7, 134.9, 128.2, 124.8, 83.8, 24.9, 21.0, 20.9.

HRMS-ESI: *m/z* calculated for $C_{34}H_{40}BO_2$ (M+H$^+$): 491.3122, measured: 491.3111.

7-diMes, 1,2,3,4,5,6-hexakis(5,7-dimesitylazulen-2-yl)benzene

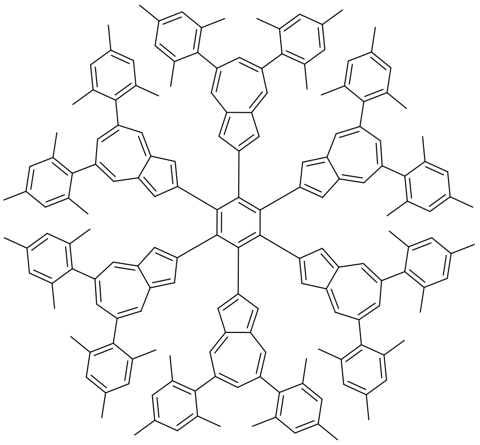

2-(5,7-dimesitylazulen-2-yl)-4,4,5,5-tetramethyl-1,3,2-dioxaborolane (**6-diMes**, 200 mg, 0.407 mmol), hexabromobenzene (22.49 mg, 0.041 µmol), Cs_2CO_3 (66.43 mg, 0.204 mmol), Pd(dppf)Cl_2 (14.92 mg, 20.39 µmol) were placed in a nitrogen-flushed Schlenk flask. Next, 10 mL of freshly distilled 1,4-dioxane and 2 mL of deoxygenated H_2O were injected into the reaction vessel, which was then immediately placed into sand bath preheated to 80°C and stirred for 1 h. After this time, solvents were evaporated under reduced pressure and reaction mixture was passed through a short silica gel column (CH_2Cl_2). Crude product was purified using size exclusion chromatography (BioBeads S-X3, toluene) yielding compound **7-diMes** as a green solid (82 mg, 0.036 mmol, 88%).

^{1}H NMR (500 MHz, $CDCl_3$) δ 7.52 (d, *J* = 1.5 Hz, 12H), 6.87 (t, *J* = 1.5 Hz, 6H), 6.79 (s, 24H), 6.77 (s, 12H), 2.25 (s, 36H), 1.80 (s, 72H).

^{13}C NMR (126 MHz, $CDCl_3$) δ 151.6, 142.2, 138.4, 138.2, 137.7, 136.2, 136.0, 135.5, 133.5, 127.9, 121.0, 21.0, 20.7.

APCI-HRMS *m/z* calculated for $C_{174}H_{163}$ (M+H$^+$): 2252.2749, measured: 2252.2651.

UV/Vis/NIR (CH_2Cl_2): 294 nm (216000 $M^{-1}cm^{-1}$), 600 nm (2500 $M^{-1}cm^{-1}$), 644 nm (2400 $M^{-1}cm^{-1}$).

2-diMes, 2,4,9,11,14,16,19,21,26,28,31,33-dodecamesitylhexaazuleno[2,1-*a*:1',2',3'-*cd*:1'',2''-*f*:2''',1'''-*j*:1'''',2'''',3''''-*lm*:1''''',2'''''-*o*]perylene

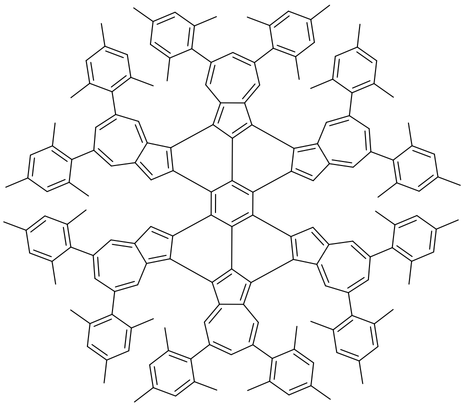

1,2,3,4,5,6-Hexakis(5,7-dimesitylazulen-2-yl)benzene (**7-diMes**, 20 mg, 8.9 µmol) was dissolved in 5 mL of deoxygenated dichloromethane under nitrogen atmosphere and the reaction flask was placed in an ice bath. Next, DDQ (6 eq., 12.1 mg, 53.3 µmol) in 5 mL of CH_2Cl_2 was added in one portion to the reaction mixture. An immediate change of colour occurred from green to brown. After 2 h of stirring at 0°C the reaction was quenched with hydrazine hydrate (51% aqueous solution, 0.05 mL) and passed through short basic Al_2O_3 column (Brockmann grade III). Crude product was purified using gel permeation chromatography (eluent: 0.5% NEt_3 in $CHCl_3$) yielding pure **2-diMes** as a brown solid (13 mg, 65%, 5.8 µmol).

^{1}H NMR (500 MHz, C_6D_6) δ 10.17 (d, *J* = 1.0 Hz, 4H), 9.76 (s, 4H), 8.90 (s, 4H), 7.89 (d, *J* = 1.5 Hz, 4H), 7.41 (s, 2H), 7.04 (t, *J* = 1.3 Hz, 4H), 6.99 (s, 4H), 6.90 (s, 8H), 6.87 (s, 4H), 6.85 (s, 4H), 6.84 (s, 4H), 2.38 (s, 9H), 2.38 (s, 9H), 2.30 (s, 9H), 2.27 (s, 9H), 1.95 (s, 9H), 1.94 (s, 9H), 1.88 (s, 9H), 1.79 (s, 9H), 1.74 (s, 9H).

^{13}C NMR (126 MHz, C_6D_6) δ 142.8, 142.6, 142.6, 141.9, 140.6, 138.1, 138.0, 136.4, 136.2, 136.1, 135.9, 135.7, 135.7, 135.7, 135.6, 135.6, 135.7, 135.6, 135.5, 135.4, 135.3, 135.0, 134.7, 133.0, 128.8, 128.7, 128.6, 128.4, 126.4, 128.2, 125.1, 123.8, 122.3, 120.6, 21.0, 21.0, 20.9, 20.8, 20.7, 20.5, 20.4, 20.4, 20.3 (two aromatic C signals overlapped).

APCI-HRMS *m/z* calculated for $C_{174}H_{155}$ (M+H$^+$): 2245.2157, measured: 2245.2103.

UV/Vis/NIR (CH_2Cl_2): 390 nm (92000 M^{-1}cm^{-1}), 1151 nm (9600 M^{-1}cm^{-1}).

X-ray single crystal diffraction

Suitable crystals were selected and measured using Rigaku XtaLAB Synergy-R diffractometer. The crystals were kept at 100 K during data collection. The structures were solved with the olex2.solve[9] or SHELXS[10] structure solution programs and refined with the SHELXL[11] refinement package using least squares minimization.[11]

Table S3. Parameters of X-ray single crystal diffraction experiment for **2-diMes** (CCDC 2473647).

	2-diMes
Crystal data	
Chemical formula	$C_{174}H_{152}{\cdot}2.5(C_7H_8){\cdot}0.35[C7H_9]{\cdot}3.5[C7H_9]$
M_r	2831.88
Crystal system, space group	Triclinic, $P\bar{1}$
Temperature (K)	100
a, b, c (Å)	15.168 (3), 21.871 (4), 28.353 (6)
α, β, γ (°)	70.71 (3), 75.74 (3), 71.46 (3)
V (Å^3)	8311 (4)
Z	2
Radiation type	Cu $K\alpha$
μ (mm^{-1})	0.48
Crystal size (mm)	0.23 × 0.10 × 0.03
Data collection	
Diffractometer	XtaLAB Synergy R, DW system, HyPix-Arc 150
Absorption correction	Multi-scan<br>*CrysAlis PRO* 1.171.43.105a (Rigaku Oxford Diffraction, 2024) Empirical absorption correction using spherical harmonics, implemented in SCALE3 ABSPACK scaling algorithm.
T_{min}, T_{max}	0.640, 1.000
No. of measured, independent and observed [$I > 2\sigma(I)$] reflections	218927, 33703, 15709
R_{int}	0.073
$(\sin \theta/\lambda)_{max}$ (Å^{-1})	0.629
Refinement	
$R[F^2 > 2\sigma(F^2)]$, $wR(F^2)$, S	0.122, 0.413, 1.07
No. of reflections	33703
No. of parameters	1978
No. of restraints	457
H-atom treatment	H-atom parameters constrained
$\Delta\rho_{max}$, $\Delta\rho_{min}$ (e Å^{-3})	0.70, −0.40

Table S4. Parameters of X-ray single crystal diffraction experiment for **7-diMes** (CCDC 2473646).

	7-diMes
Crystal data	
Chemical formula	$C_{174}H_{162}·5(C_7H_8)·$[+solvents]
M_r	2825.46
Crystal system, space group	Monoclinic, $C2/c$
Temperature (K)	100
a, b, c (Å)	24.255 (5), 35.541 (7), 20.452 (4)
β (°)	101.71 (3)
V (Å^3)	17264 (6)
Z	4
Radiation type	Cu $K\alpha$
μ (mm^{-1})	0.46
Crystal size (mm)	0.39 × 0.14 × 0.10
Data collection	
Diffractometer	XtaLAB Synergy R, DW system, HyPix-Arc 150
Absorption correction	Multi-scan<br>*CrysAlis PRO* 1.171.42.63a (Rigaku Oxford Diffraction, 2022) Empirical absorption correction using spherical harmonics, implemented in SCALE3 ABSPACK scaling algorithm.
T_{min}, T_{max}	0.486, 1.000
No. of measured, independent and observed [$I > 2\sigma(I)$] reflections	53754, 16529, 9489
R_{int}	0.113
(sin θ/λ)$_{max}$ (Å^{-1})	0.623
Refinement	
$R[F^2 > 2\sigma(F^2)]$, $wR(F^2)$, S	0.097, 0.307, 1.04
No. of reflections	16529
No. of parameters	1013
No. of restraints	54
H-atom treatment	H-atom parameters constrained
$\Delta\rho_{max}$, $\Delta\rho_{min}$ (e Å^{-3})	0.46, −0.30

Cations sensing experiments

Materials and methods

Regarding metal cation binding experiments (UV/Vis titrations) the following water was used: Merck, product no. 1.15333.2500. For the titrations, cations were introduced as hexafluorophosphate (for Li^+, Na^+, K^+, Cs^+) or tetrafluoroborate (for Rb^+) salts.

Titration experiments methodology

Stock solution of **2-diMes** was prepared by dissolving a given weighted amount of the compound in tetrahydrofuran (THF) to a concentration of $2{\cdot}10^{-4}$ M. The sample for titration was prepared by diluting the stock solution with THF and water to obtain a 1/1, *v*/*v* sample with a concentration of $2{\cdot}10^{-5}$ M. Regarding titrations the following water was used: Merck, product no. 1.15333.2500). Each titration experiment consisted of 10 steps. Initially, the UV/Vis spectrum of pure **2-diMes** was measured. Subsequently, cation solutions were introduced in 9 consecutive steps to provide the following molar equivalents of cation to **2-diMes**: 0.25, 0.5, 0.75, 1, 2, 3, 5, 10, 20. During the titration experiment, the solution in the cuvette was well-mixed with a magnetic stirrer (1200 rpm, mixing for about 30 s after the addition of a cation). Notably, the fresh solution of native **2-diMes** was prepared for each titration experiment, and the absorbance of this sample was taken as the "0" point for further analysis. Absorption maximum (λ_{max}) of 396 nm was taken for Bindfit analyses.[12–14] Association constant (K_a) value was taken for the best fit (best model with the most satisfying covariance and RMS value; models with the negative K_a or K_a error values, as well as enormously high values, were excluded). For the systems in which comparative fitting qualities were found, both fits were considered (i.e. systems with Li^+, K^+ and Cs^+, see data in Section "Titration experiments").

Table S5. Comparison of binding parameters for **2-diMes** and tested s-block metal cations.

**Cation**	**K_a (10^4 M^{-1})**	**Fitting model[a]**
Li^+	0.15±0.01<br>1.22±0.09	1:1<br>2:1, statistical
Na^+	4.27±1.02	2:1, statistical
K^+	1.35±0.18<br>1.03±0.11	1:1<br>2:1, statistical
Rb^+	3.30±0.43	2:1, non-cooperative
Cs^+	1.99±0.30<br>1.83±0.29	1:1<br>2:1, non-cooperative

[a] data shown for best fitting parameters quality.

Titration experiments

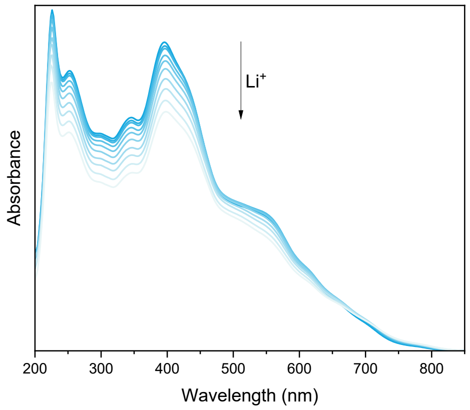

Figure S2. UV-vis spectra of **2-diMes** (THF:H_2O = 1/1 *v*/*v*, $2 \cdot 10^{-5}$ M) in the presence of various molar equivalents of Li^+.

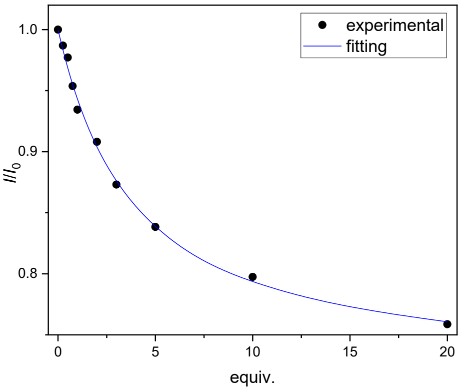

Figure S3. Titration curve and global fitting (Bindfit) regarding interactions between **2-diMes** and Li^+ in water (fitting conditions: model 2:1 statistical, Nelder-Mead method-algorithm, dilution correction, fitting parameters: K_a = 1.22 $\times 10^4$ M^{-1}, error: 7.64%, RMS = 7.29×10‒3, covariance = 3.25×10^{-3}).

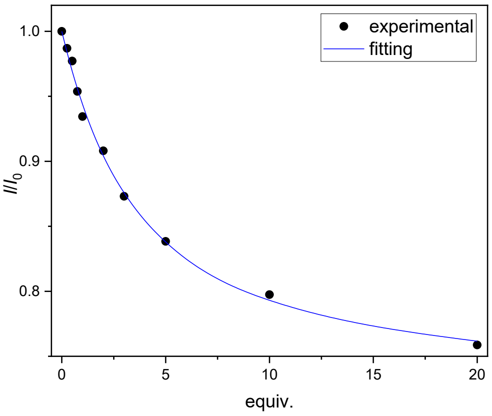

Figure S4. Titration curve and global fitting (Bindfit) regarding interactions between **2-diMes** and Li^+ in water (fitting conditions: model 1:1, Nelder-Mead method-algorithm, dilution correction, fitting parameters: K_a = 1.54 $\times 10^3$ M^{-1}, error: 9.36%, RMS = 7.41×10^{-3}, covariance = 3.38×10^{-3}).

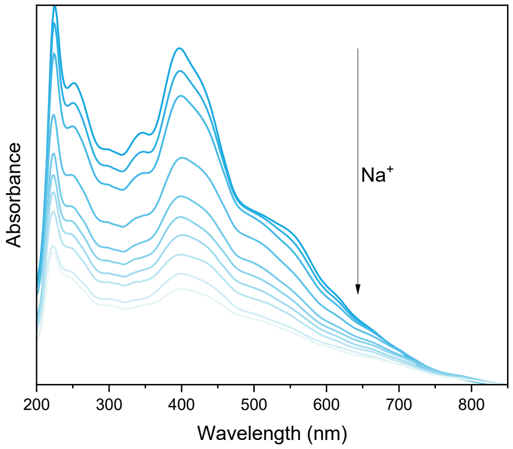

Figure S5. UV/Vis spectra of **2-diMes** (THF:H_2O = 1/1 *v*/*v*, $2{\cdot}10^{-5}$ M) in the presence of various molar equivalents of Na^+.

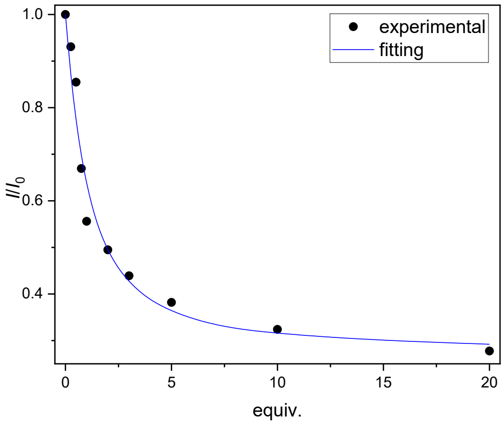

Figure S6. Titration curve and global fitting (Bindfit) regarding interactions between **2-diMes** and Na^+ in water (fitting conditions: model 2:1 statistical, Nelder-Mead method-algorithm, dilution correction, fitting parameters: K_a = 4.27 $\times 10^4$ M^{-1}, error: 24.29%, RMS = 5.68×10^{-2}, covariance = 2.97×10^{-2}).

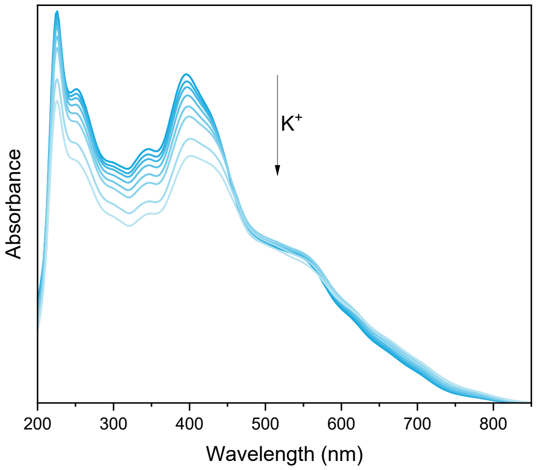

Figure S7. UV/Vis spectra of **2-diMes** (THF:H_2O = 1/1 *v*/*v*, $2{\cdot}10^{-5}$ M) in the presence of various molar equivalents of K^+.

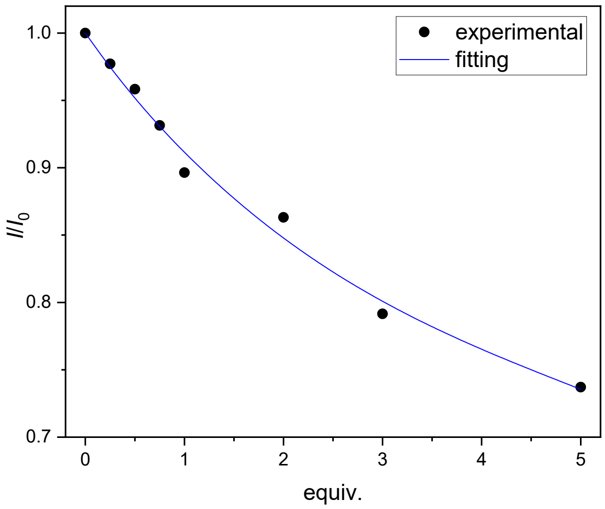

Figure S8. Titration curve and global fitting (Bindfit) regarding interactions between **2-diMes** and K^+ in water (fitting conditions: model 2:1 statistical, Nelder-Mead method-algorithm, dilution correction, fitting parameters: K_a = 1.03 ×10^4 M^{-1}, error: 11.03%, RMS = 1.14×10^{-2}, covariance = 1.00×10^{-2}).

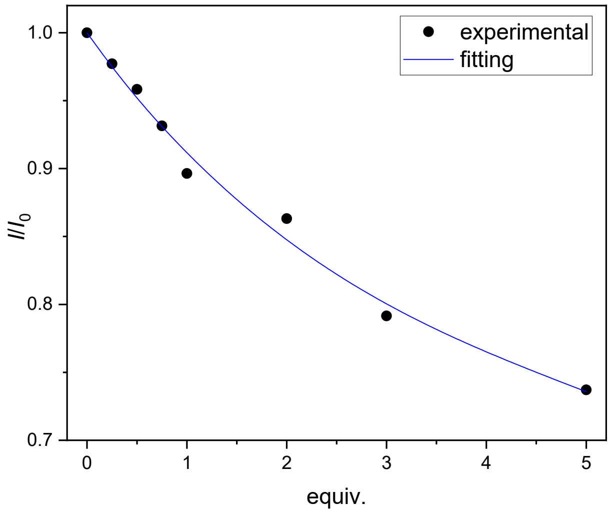

Figure S9. Titration curve and global fitting (Bindfit) regarding interactions between **2-diMes** and K^+ in water (fitting conditions: model 1:1 statistical, Nelder-Mead method-algorithm, dilution correction, fitting parameters: K_a = 1.35 ×10^4 M^{-1}, error: 13.22%, RMS = 1.14×10^{-2}, covariance = 1.00×10^{-2}).

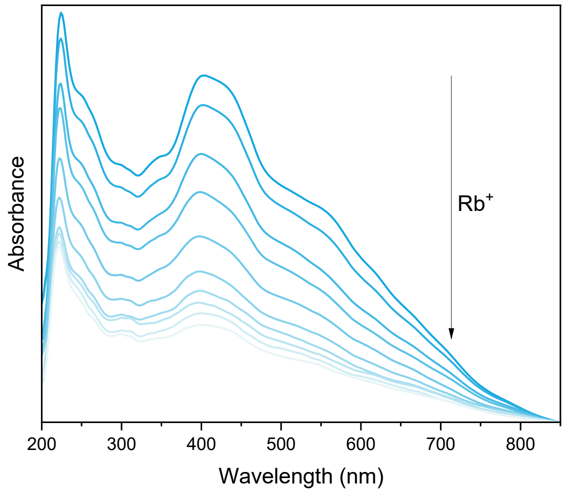

Figure S10. UV/Vis spectra of **2-diMes** (THF:H_2O = 1/1 *v*/*v*, $2{\cdot}10^{-5}$ M) in the presence of various molar equivalents of Rb^+.

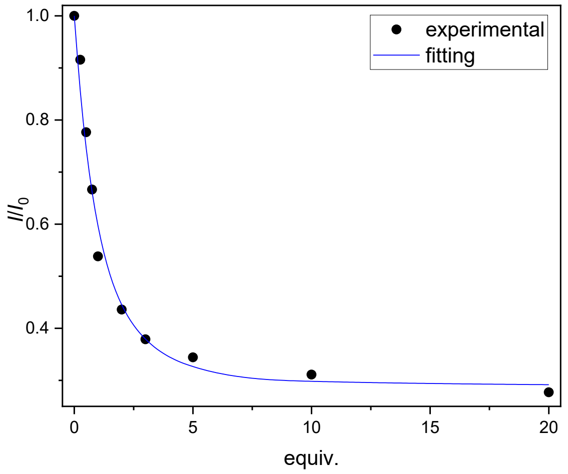

Figure S11. Titration curve and global fitting (Bindfit) regarding interactions between **2-diMes** and Rb^+ in water (fitting conditions: model 2:1 non-cooperative, Nelder-Mead method-algorithm, dilution correction, fitting parameters: K_a = 3.30 ×10^4 M^{-1}, error: 12.97%, RMS = 2.20×10^{-2}, covariance = 1.39×10^{-2}).

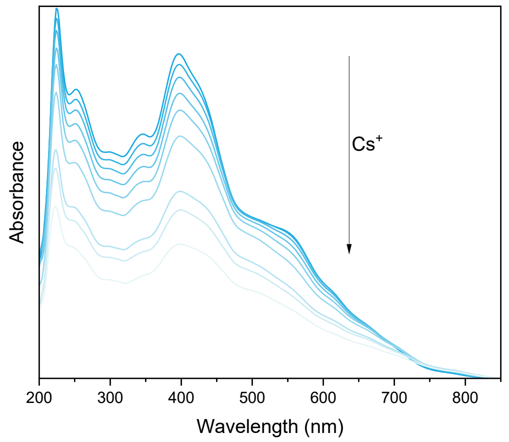

Figure S12. UV-Vis spectra of **2-diMes** (THF:H_2O = 1/1 *v*/*v*, $2{\cdot}10^{-5}$ M) in the presence of various molar equivalents of Cs^+.

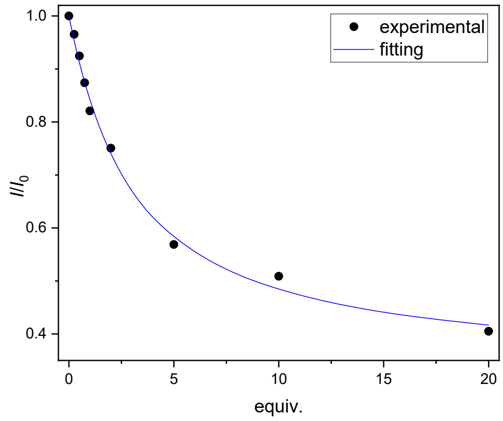

Figure S13. Titration curve and global fitting (Bindfit) regarding interactions between **2-diMes** and Cs^+ in water (fitting conditions: model 2:1 non-cooperative, Nelder-Mead method-algorithm, dilution correction, fitting parameters: K_a = 1.83 ×10^4 M^{-1}, error: 16.25%, RMS = 2.05×10^{-3}, covariance = 4.93×10^{-3}).

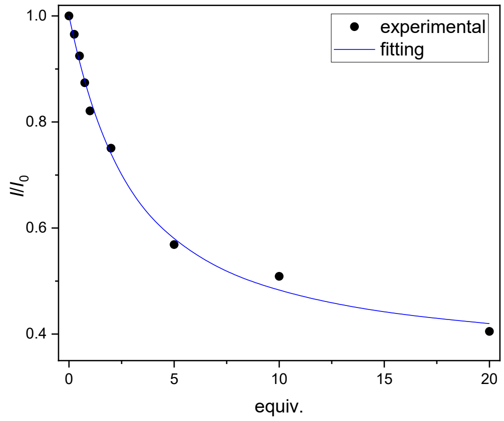

Figure S14. Titration curve and global fitting (Bindfit) regarding interactions between **2-diMes** and Cs^+ in water (fitting conditions: model 1:1, Nelder-Mead method-algorithm, dilution correction, fitting parameters: K_a = 1.99 ×10^4 M^{-1}, error: 15.17%, RMS = 2.08×10^{-3}, covariance = 5.15×10^{-3}).

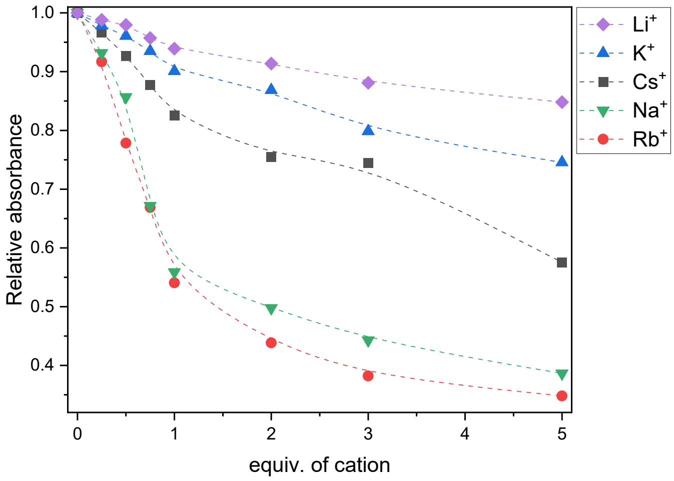

Figure S15. Comparison of titration curves (up to 5 equiv. of a cation).

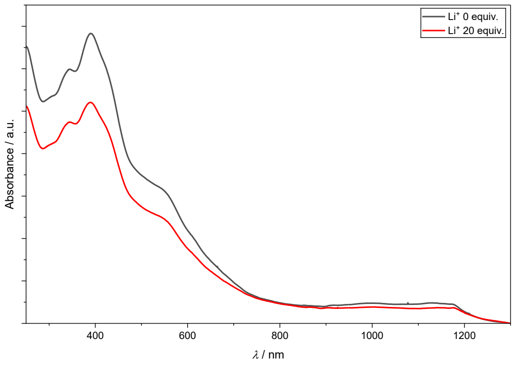

Figure S16. UV/Vis/NIR spectra of **2-diMes** and **2-diMes** in a presence of 20 equiv. of Li^{+} salt (THF/H_2O), 1/1).

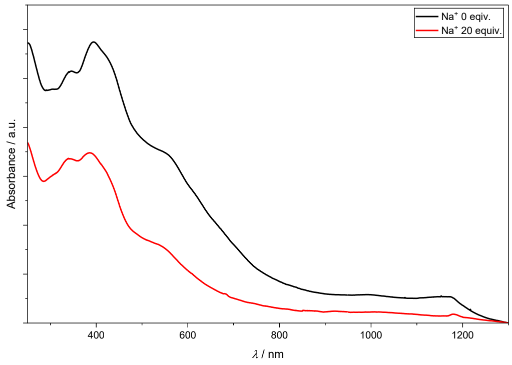

Figure S17. UV/Vis/NIR spectra of **2-diMes** and **2-diMes** in a presence of 20 equiv. of Na^{+} salt (THF/H_2O), 1/1).

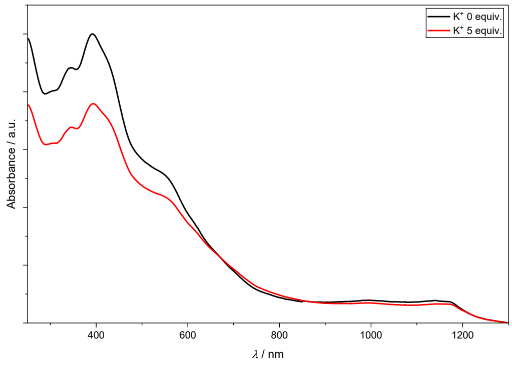

Figure S18. UV/Vis/NIR spectra of **2-diMes** and **2-diMes** in a presence of 5 equiv. of K^{+} salt (THF/H_2O), 1/1).

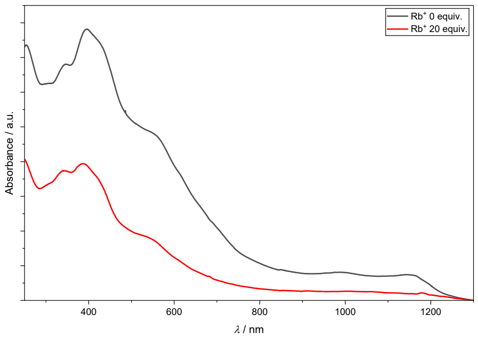

Figure S19. UV/Vis/NIR spectra of **2-diMes** and **2-diMes** in a presence of 20 equiv. of Rb^+ salt (THF/H_2O), 1/1).

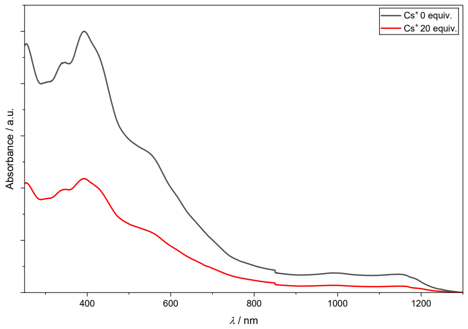

Figure S20. UV/Vis/NIR spectra of **2-diMes** and **2-diMes** in a presence of 20 equiv. of Rb^+ salt (THF/H_2O), 1/1).

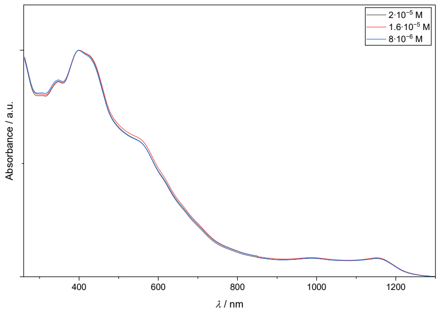

Figure S21. Concentration-dependent UV/Vis/NIR spectrum of **2-diMes** (THF/H_2O, 1/1, 25°C).

Photoacoustic (PA) and photothermal deflection spectroscopy (PDS)

Photoacoustic (PA) and **photothermal deflection spectroscopy** (PDS) were employed to determine the optical absorption through photothermal conversion processes. For both methods, thin-film samples were prepared by drop-casting DCM solutions onto Spectrosil 2000 substrates. PDS and PA enable the measurement of a signal proportional to absorbance with a high dynamic range while remaining insensitive to light scattering and other unwanted effects present in UV/VIS spectroscopy.

The PA spectrum was measured using the gas-microphone method. Optical excitation was provided by a tuneable light source consisting of a 200 W quartz-tungsten halogen lamp (Newport 67011 Research QTH Lamp) and a grating monochromator (Andor Kymera 328i). The beam was mechanically modulated at 10 Hz, and the excitation wavelength was scanned across the desired spectral range. Generated pressure waves were detected by an acoustic transducer coupled to a lock-in amplifier (Stanford Research Systems, SR860).

PDS measurement was performed in a custom setup working in the transverse configuration. The thin film sample was placed in a quartz cuvette filled with an immersion liquid, while for the DCM solution the primary solvent acted as a thermooptic liquid. Both were irradiated by a normally incident mechanically modulated, monochromatic pump beam. A probing laser beam was directed parallel to the surface of the sample and its deflection was detected by a quadrant photodiode (Newport 2901). The light absorption of the material causes the immersion liquid to heat up, thereby altering its refractive index, deflecting the probing beam. The signal from the photodiode was demodulated by a lock-in amplifier.

The sub-gap absorption data A(E) were fitted to the Urbach formula assuming linear dependence of the PA and PDS signal on the optical absorption coefficient, leading to determination of the Urbach energy (E_U) representing the electronic disorder:

$$A(E) = A_0 \exp\left(\frac{E}{E_U}\right)$$

where A_0 - material constant, E – photon energy.

The photostability test was performed upon illumination at a power density of 222 W/cm^2 at an excitation wavelength of 980 nm on the same samples used for the spectroscopic measurements, at a temperature of 293 K, in ambient air conditions. The photoacoustic signal amplitude was measured over a period of 60 minutes with 1-second intervals.

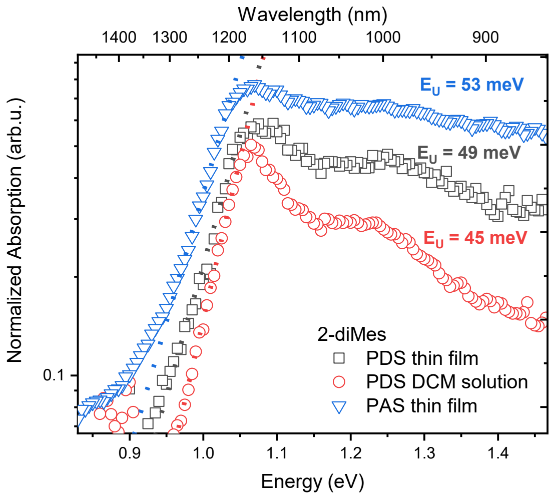

Figure S22. Absorption edge of **2-diMes** thin film and DCM solution measured by PAS and PDS, with corresponding Urbach energies: 53 meV (PAS thin film), 49 meV (PDS thin film), and 45 meV (PDS solution).

NMR Spectra

4-diMes, 1-butyl-3,5-dimesitylpyridin-1-ium bromide

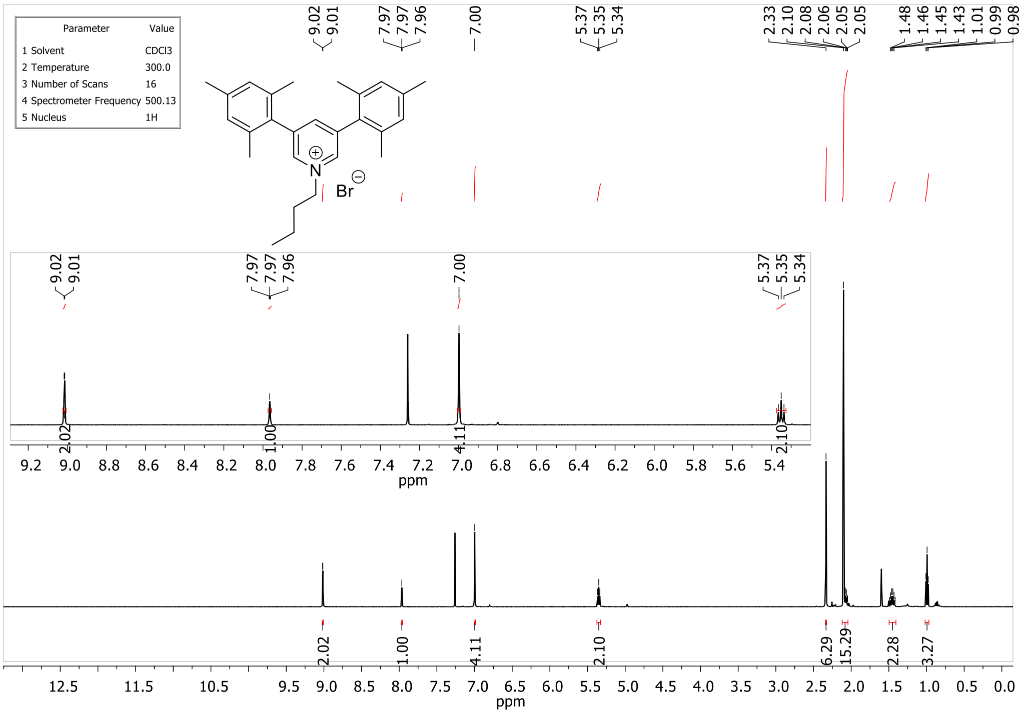

Figure S23. ^{1}H NMR spectrum of **4-diMes** ($CDCl_3$, 500 MHz, 300K).

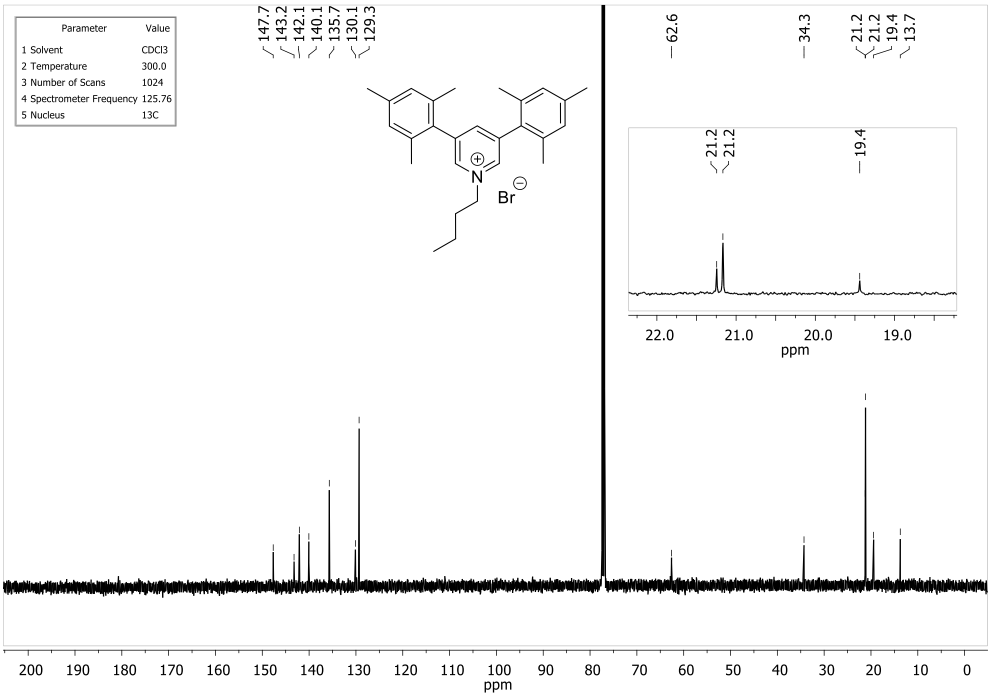

Figure S24. ^{13}C NMR spectrum of **4-diMes** ($CDCl_3$, 126 MHz, 300K).

5-diMes, 5,7-dimesitylazulene

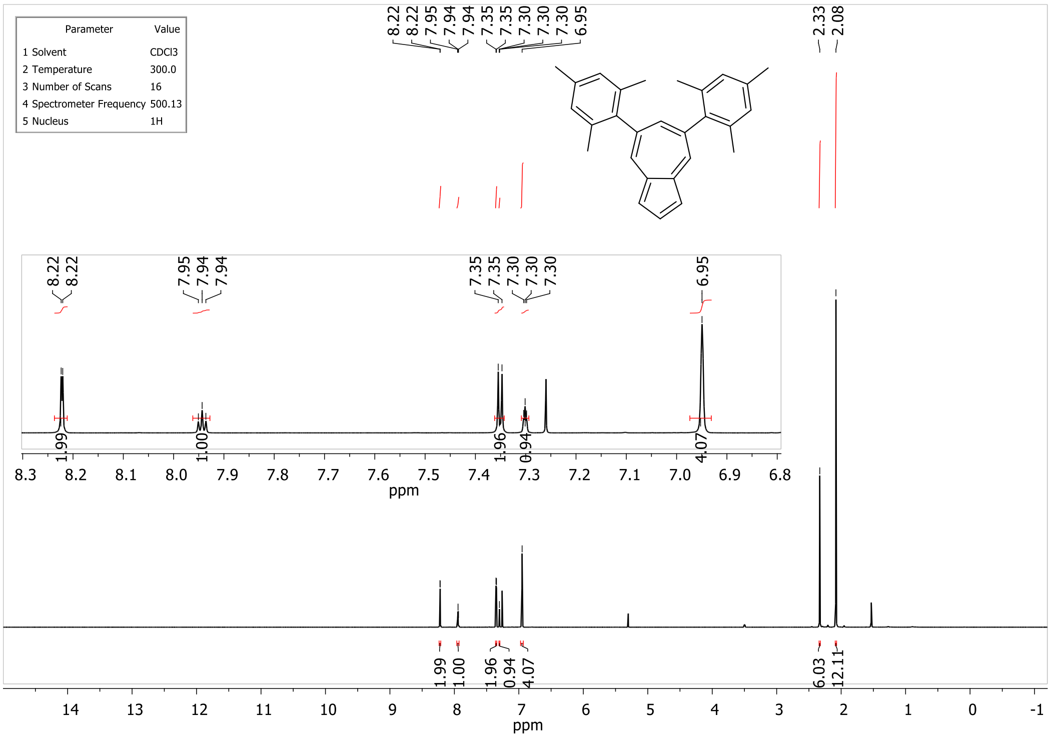

Figure S25. ^{1}H NMR spectrum of **5-diMes** ($CDCl_3$, 500 MHz, 300K).

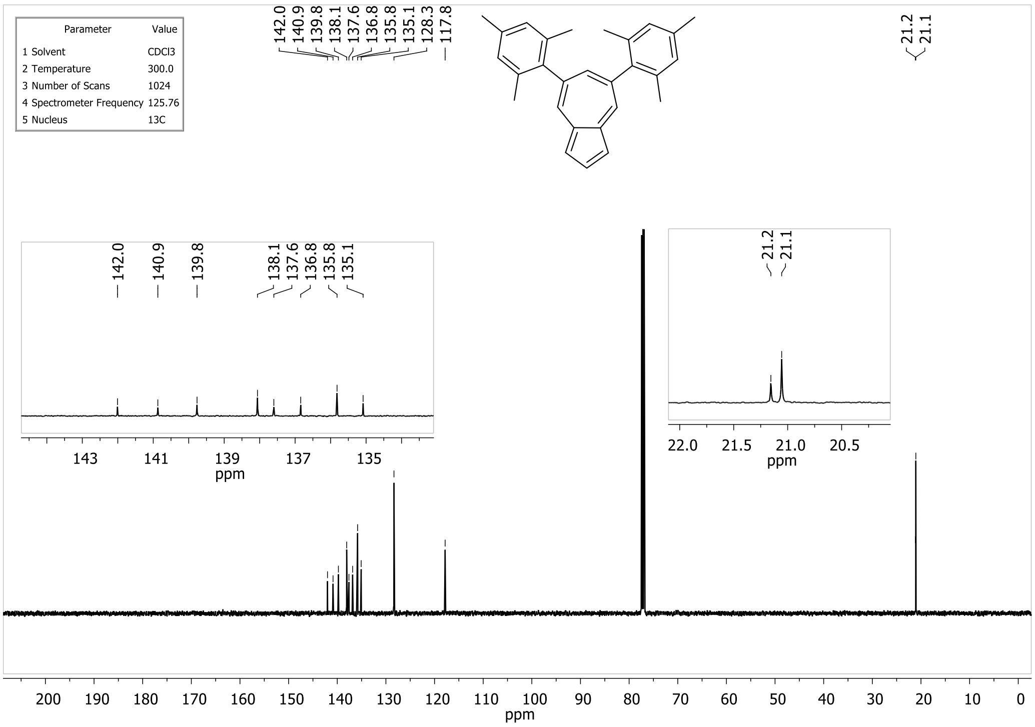

Figure S26. ^{13}C NMR spectrum of **5-diMes** ($CDCl_3$, 126 MHz, 300K).

6-diMes, 2-(5,7-dimesitylazulen-2-yl)-4,4,5,5-tetramethyl-1,3,2-dioxaborolane

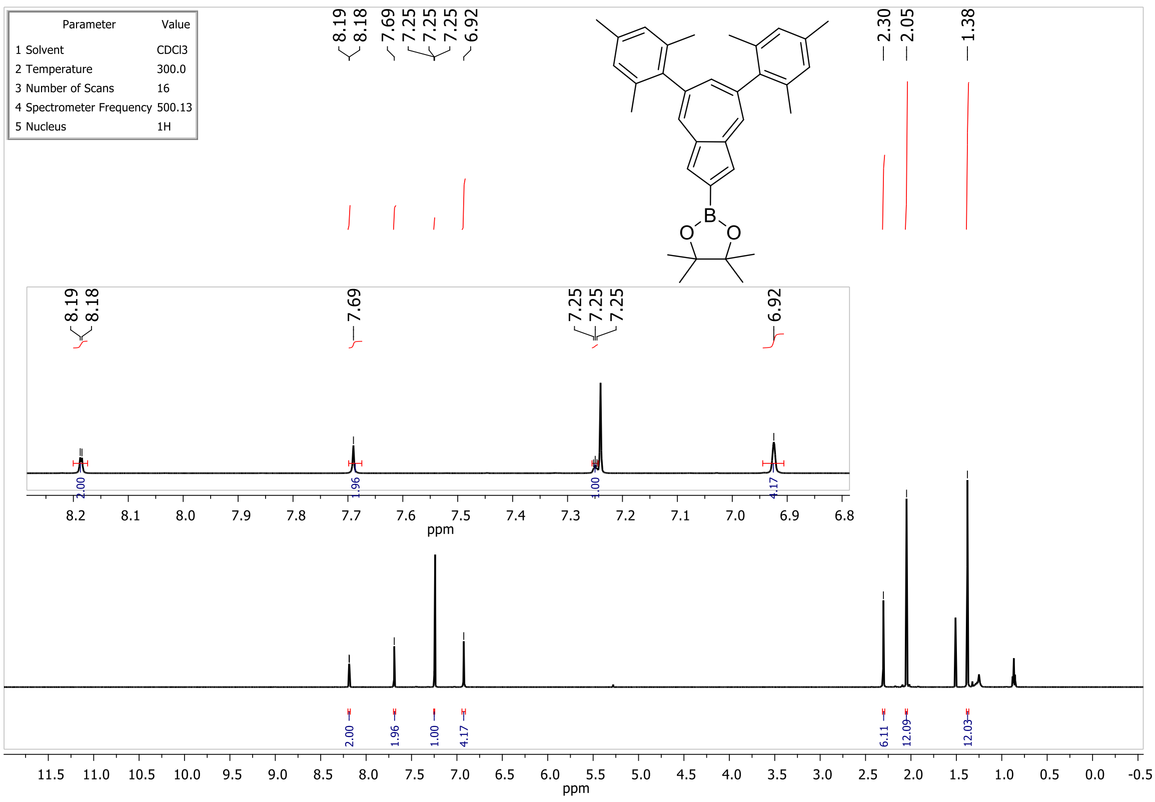

Figure S27. ^{1}H NMR spectrum of **6-diMes** ($CDCl_3$, 500 MHz, 300K).

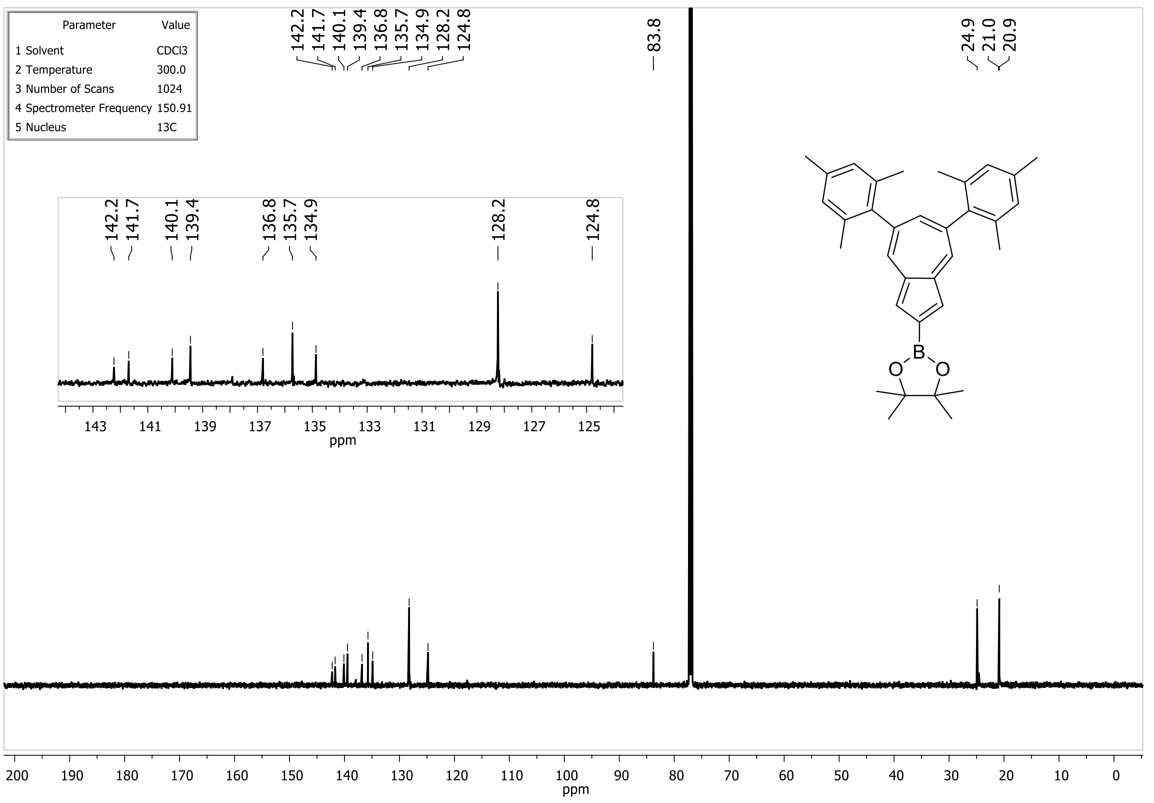

Figure S28. ^{13}C NMR spectrum of **6-diMes** ($CDCl_3$, 126 MHz, 300K).

7-diMes, 1,2,3,4,5,6-hexakis(5,7-dimesitylazulen-2-yl)benzene

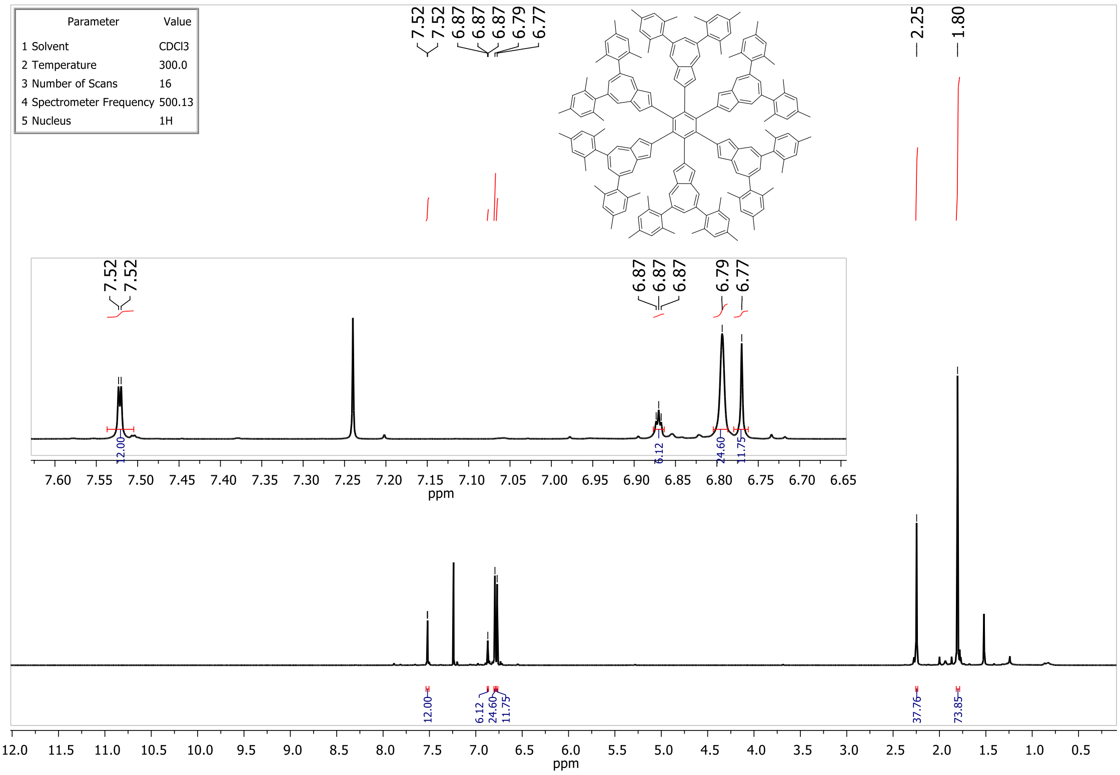

Figure S29. ^{1}H NMR spectrum of **7-diMes** ($CDCl_3$, 500 MHz, 300K).

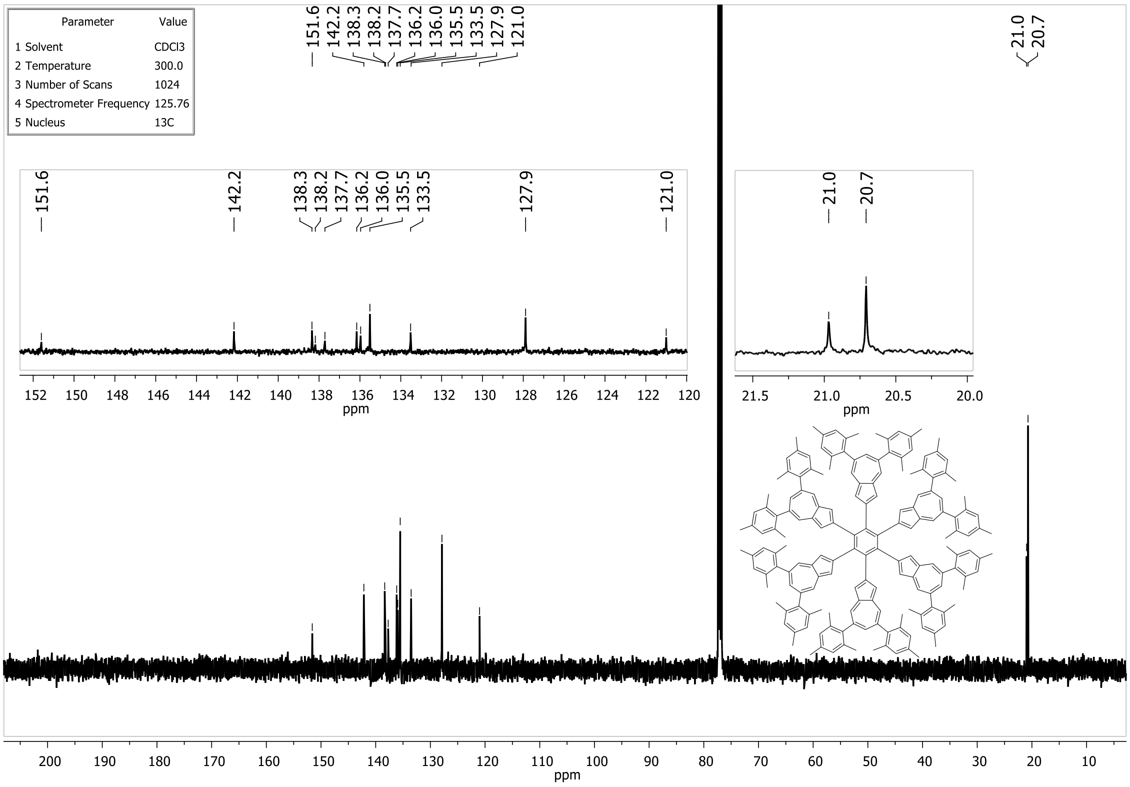

Figure S30. ^{13}C NMR spectrum of **7-diMes** ($CDCl_3$, 126 MHz, 300K).

2-diMes, 2,4,9,11,14,16,19,21,26,28,31,33-dodecamesitylhexaazuleno[2,1-*a*:1',2',3'-*cd*:1'',2''-*f*:2''',1'''-*j*:1'''',2'''',3''''-*lm*:1''''',2'''''-*o*]perylene

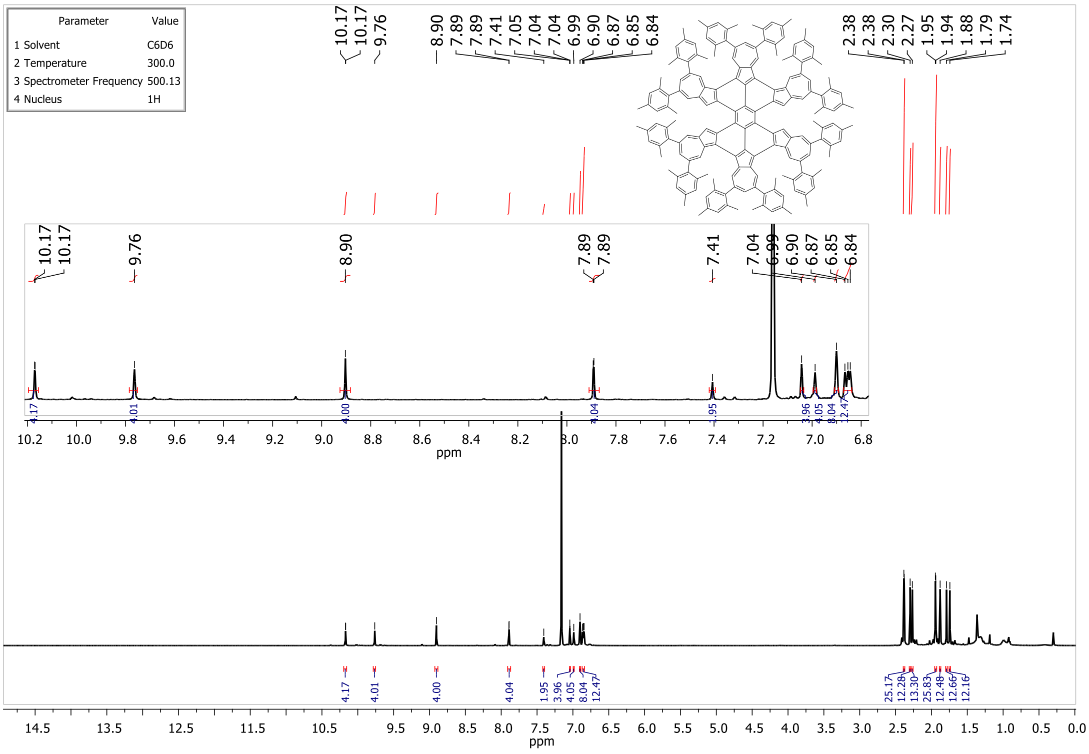

Figure S31. ^{1}H NMR spectrum of **2-diMes** (C_6D_6, 500 MHz, 300K).

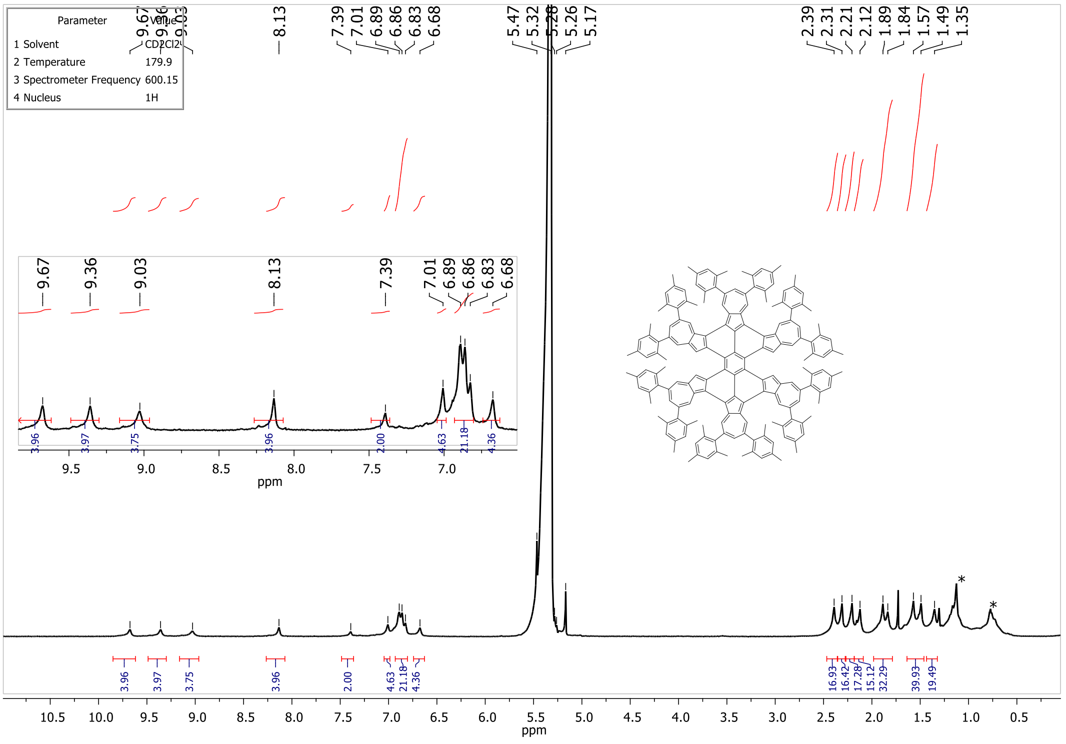

Figure S32. ^{1}H NMR spectrum of **2-diMes** (CD_2Cl_2, 600 MHz, 180K).

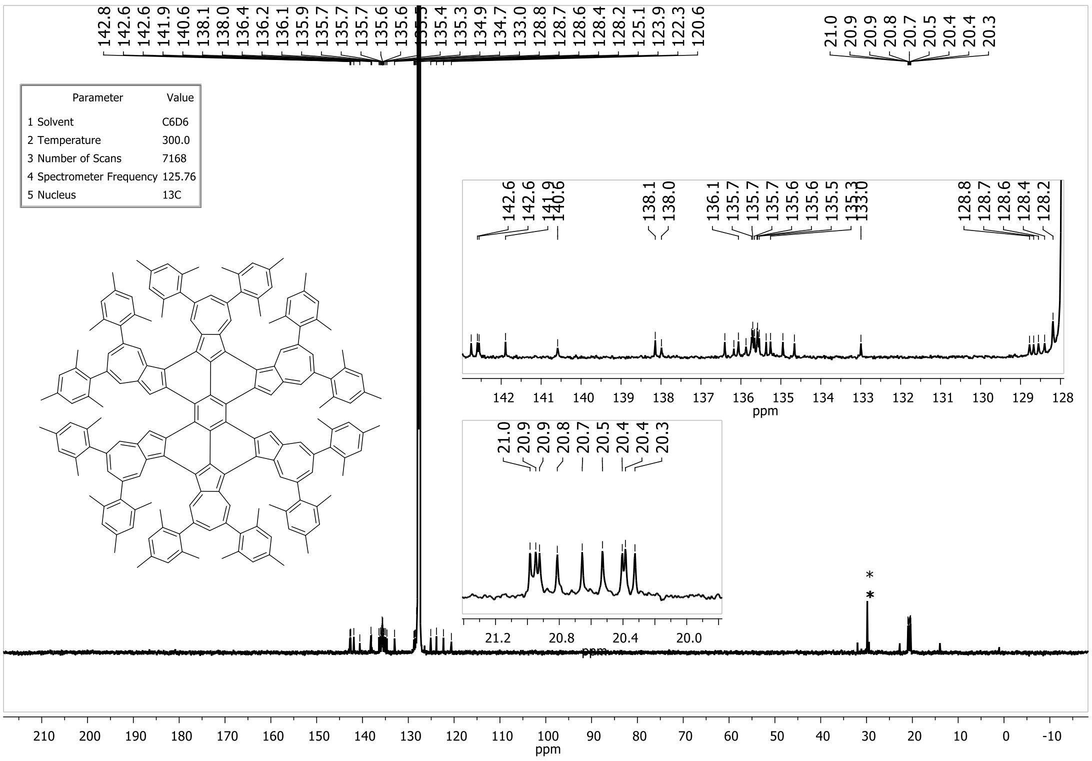

Figure S33. ^{13}C NMR spectrum of **2-diMes** (C_6D_6, 126 MHz, 300K).

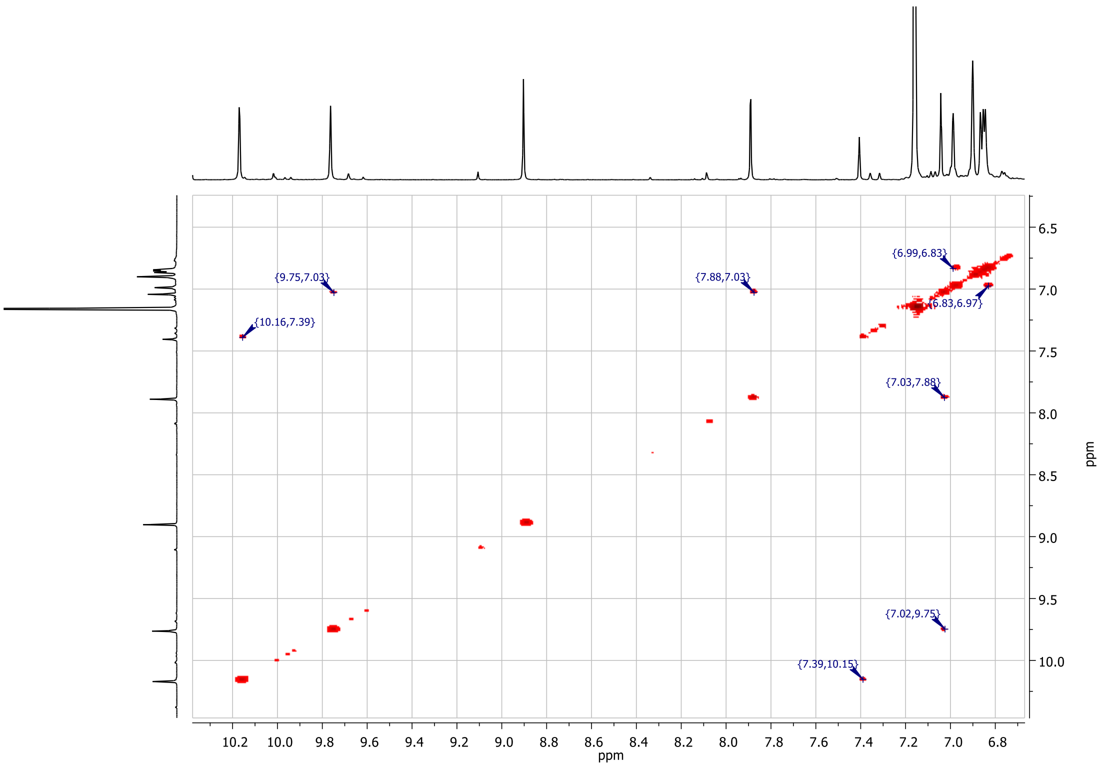

Figure S34. COSY spectrum of **2-diMes** (C_6D_6, 300K).

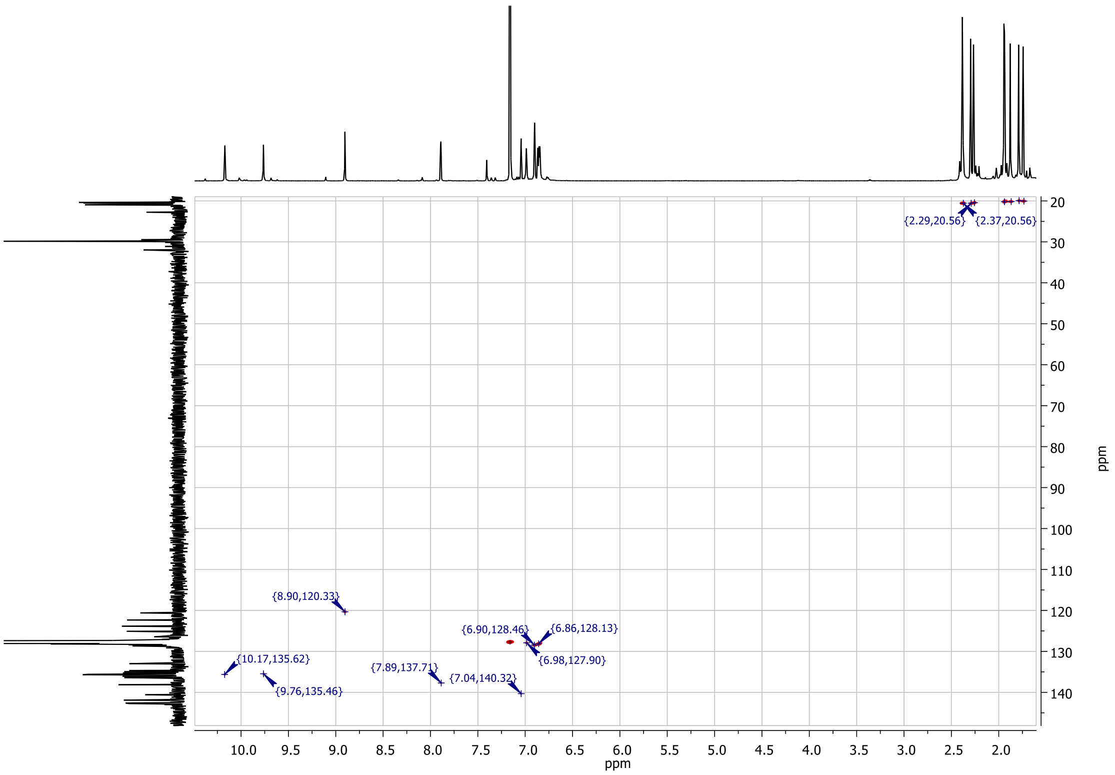

Figure S35. HSQC spectrum of **2-diMes** (C_6D_6, 300K).

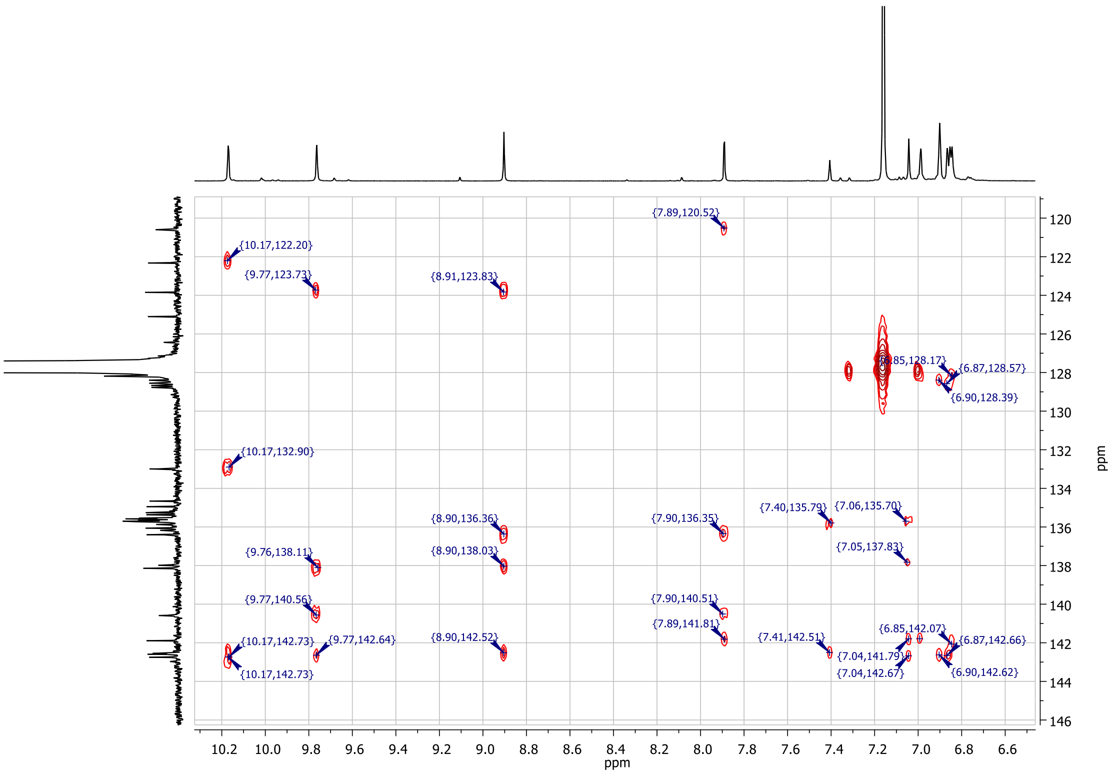

Figure S36. Part of HMQC spectrum of **2-diMes** (C_6D_6, 300K).

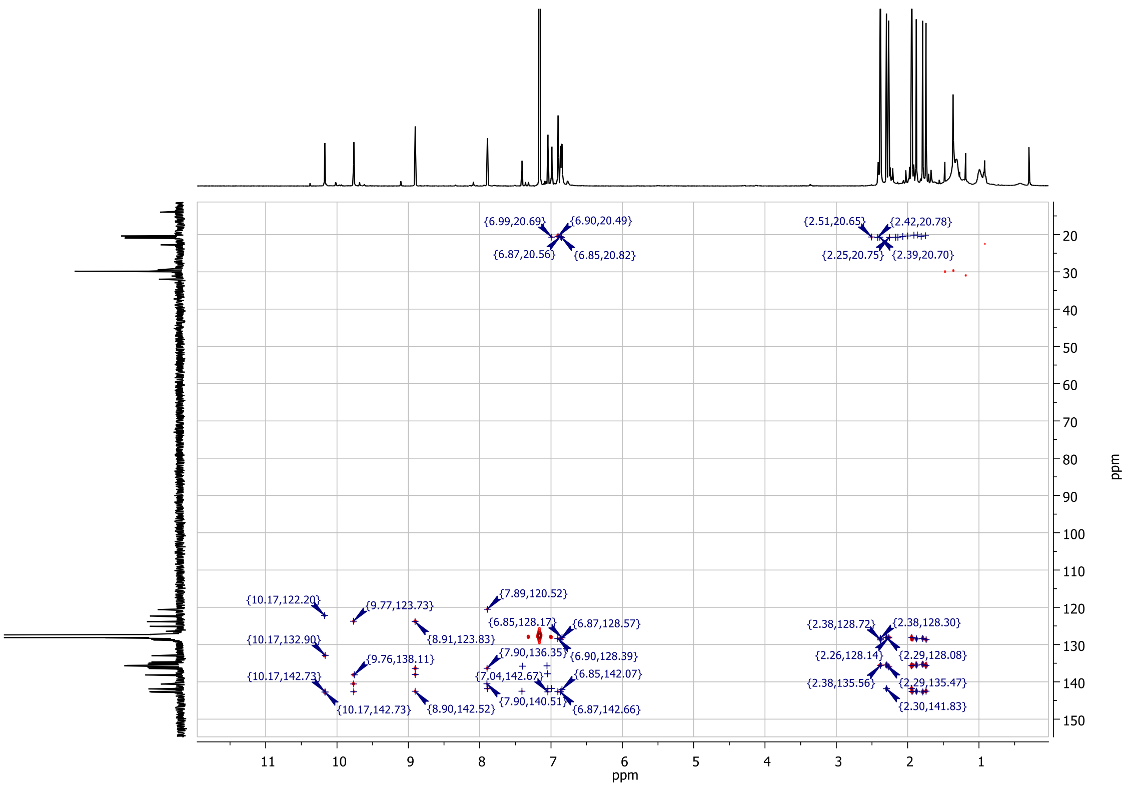

Figure S37. HMQC spectrum of **2-diMes** (C_6D_6, 300K).

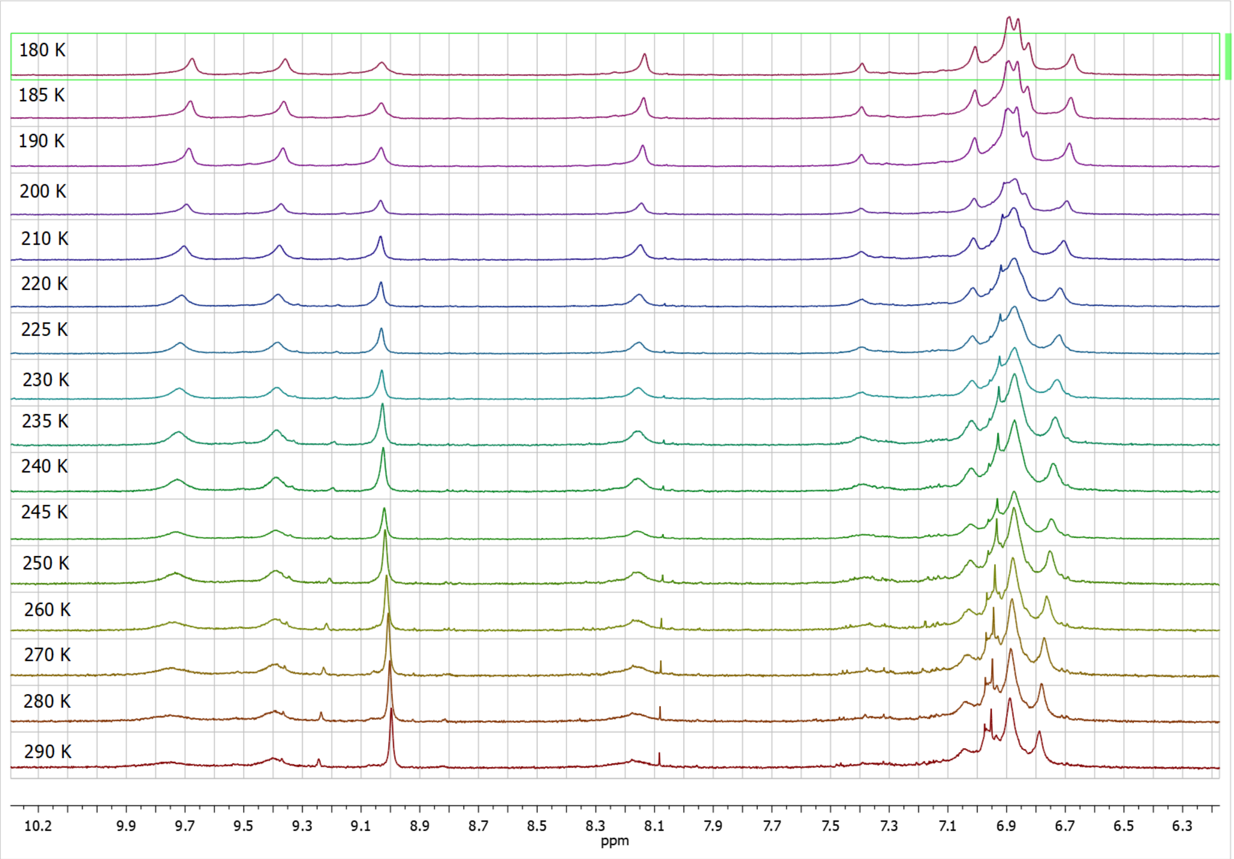

Figure S38. Variable-temperature ^{1}H NMR spectra of **2-diMes** in CD_2Cl_2, 600 MHz.

Electrochemistry

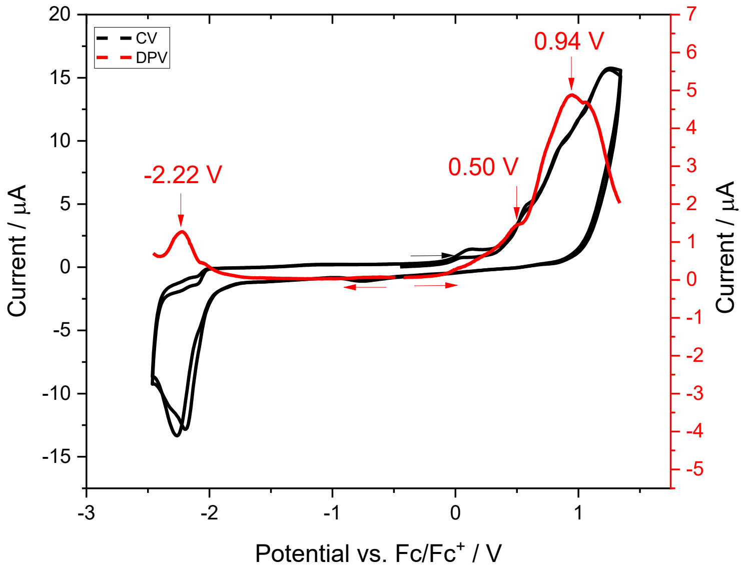

Figure S39. CV (black) and DPV (red) of compound **7-diMes** ($[NBu_4][PF_6]$ in CH_2Cl_2 (0.1 M) as a supporting electrolyte, 50 mV/s scanning rate).

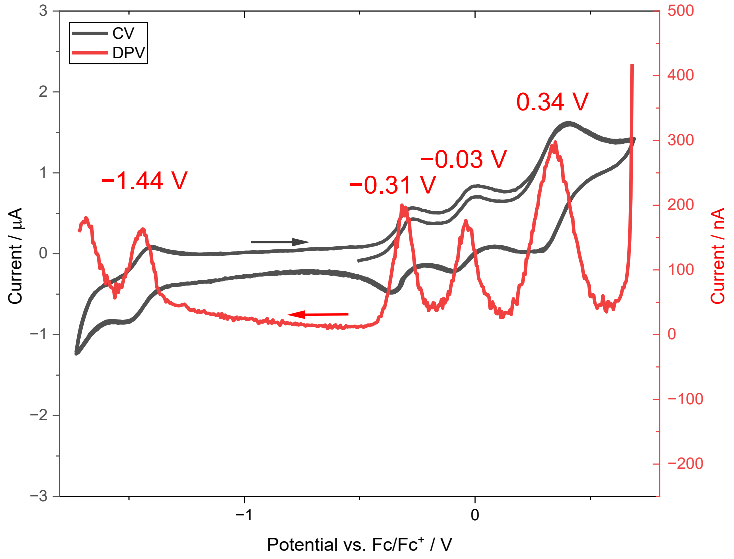

Figure S40. CV (black) and DPV (red) of compound **2-diMes** ($[NBu_4][PF_6]$ in CH_2Cl_2 (0.1 M) as a supporting electrolyte, 50 mV/s scanning rate).

HRMS spectra

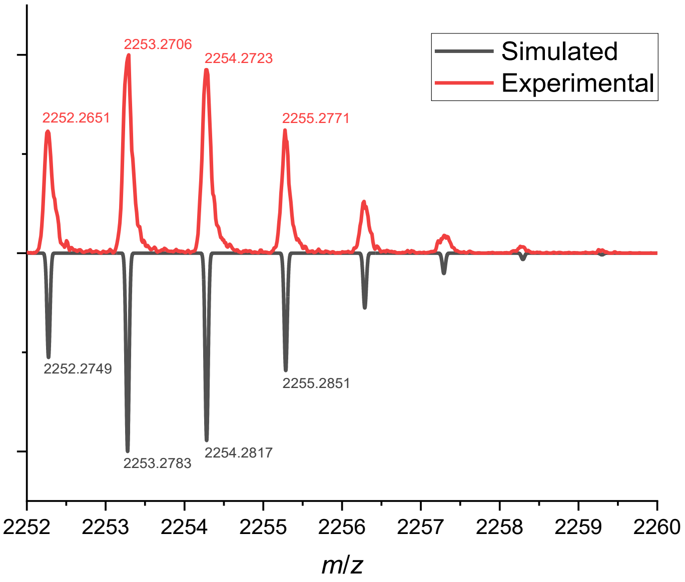

Figure S41. APCI-HRMS spectrum of **7-diMes.**

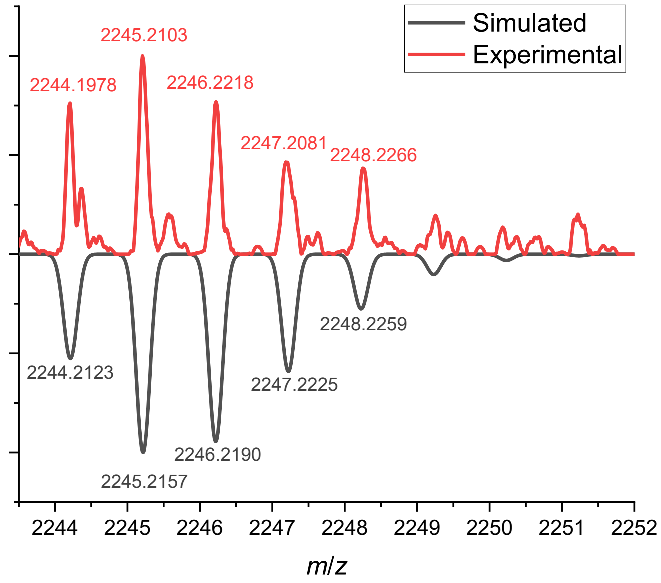

Figure S42. APCI-HRMS spectrum of **2-diMes.**

UV/Vis/NIR and fluorescence spectra

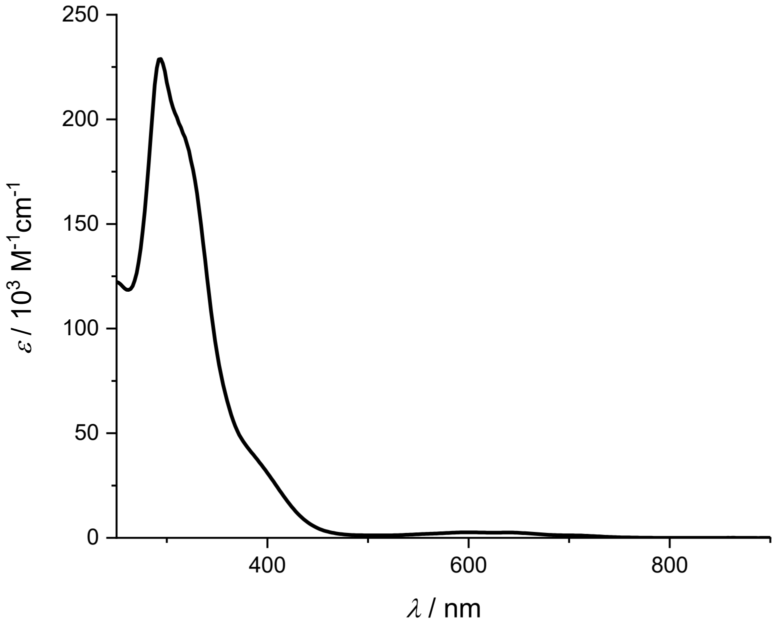

Figure S43. UV/Vis/NIR spectrum of **7-diMes** (CH_2Cl_2, c~10^{-6} M, 20°C).

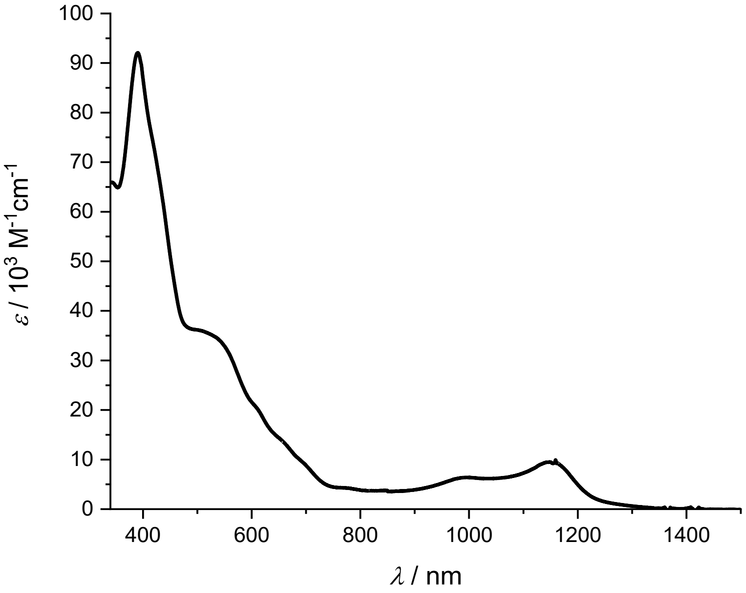

Figure S44. UV/Vis/NIR spectrum of **2-diMes** (CH_2Cl_2, c~10^{-6} M, 20°C).

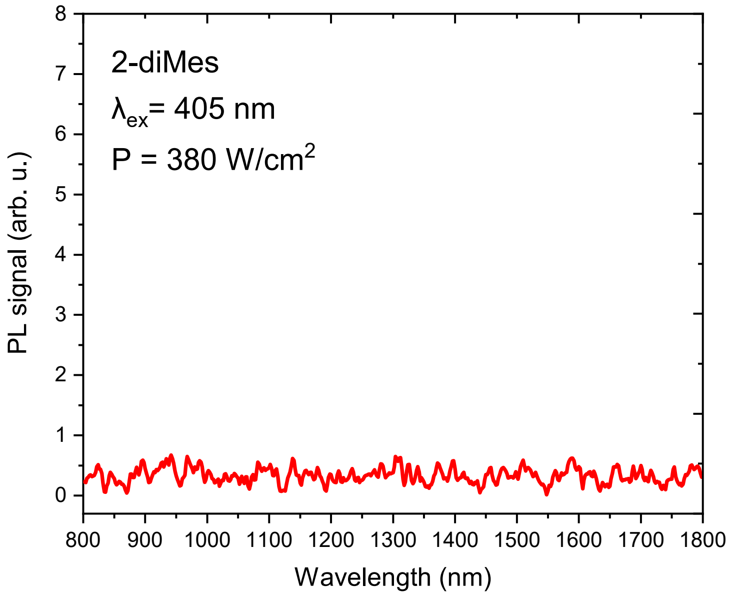

Figure S45. Emission of **2-diMes** (CH_2Cl_2 solution).

Spectroelectrochemistry

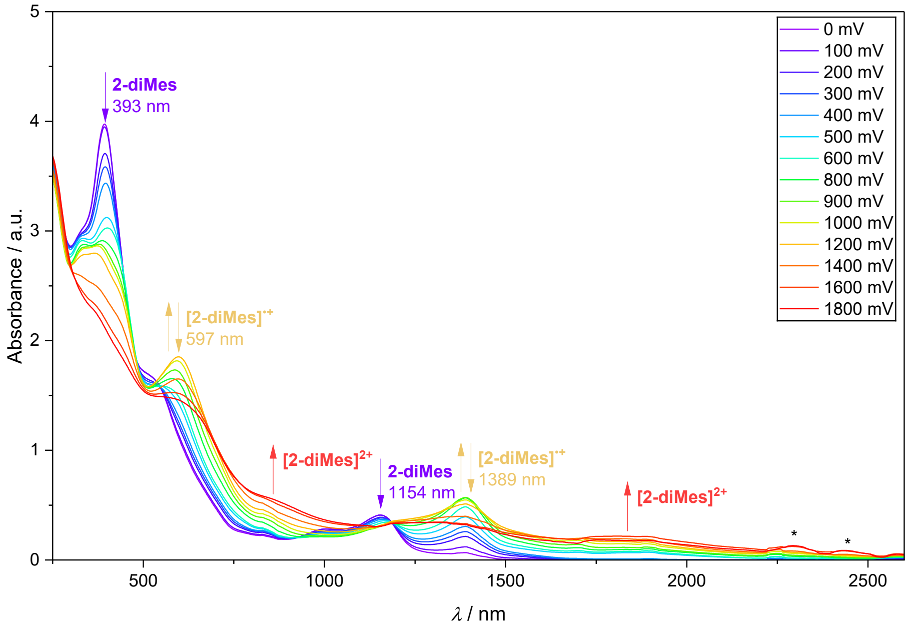

Figure S46. Spectroelectrochemical UV/Vis/NIR measurements of **2-diMes** (c~10^{-5} M, 20°C in 0.1 M N[nBu$_4$]PF$_6$ CH_2Cl_2 solution). Platinum grid working electrode, platinum counter, and a Ag/AgCl chloride reference electrode. Potentials given versus a reference electrode.

DFT calculations

Molecular frontier orbitals

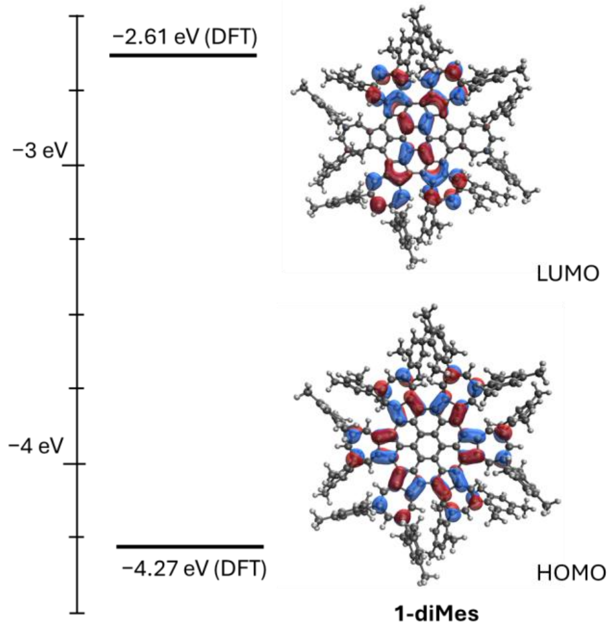

Figure S47. Molecular frontier orbitals of **1-diMes**, B3LYP/6-31g(d,p).

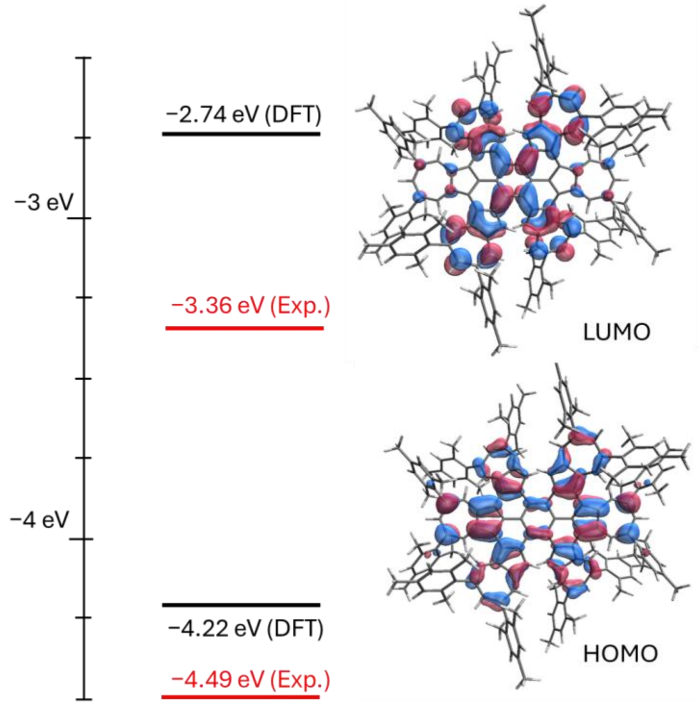

2-diMes

Figure S48. Molecular frontier orbitals of **2-diMes**, B3LYP/6-31g(d,p) and experimental HOMO and LUMO levels.

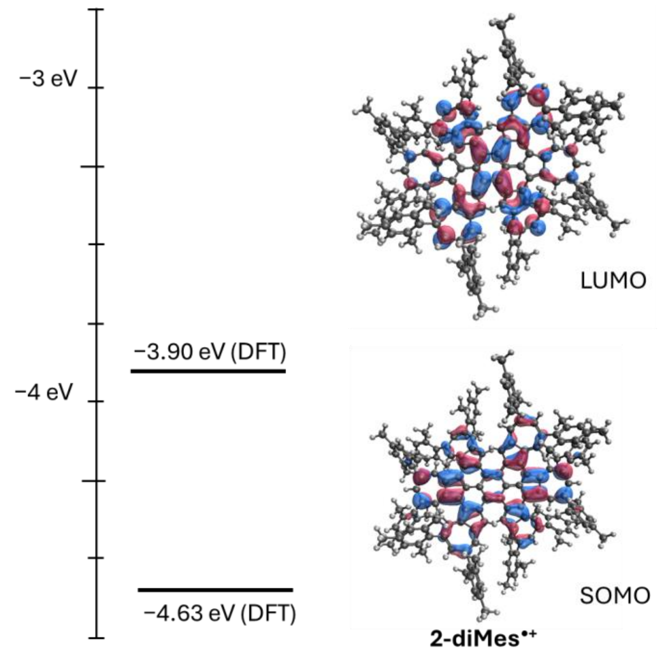

Figure S49. Molecular frontier orbitals of **[2-diMes]•+**, UB3LYP/6-31g(d,p).

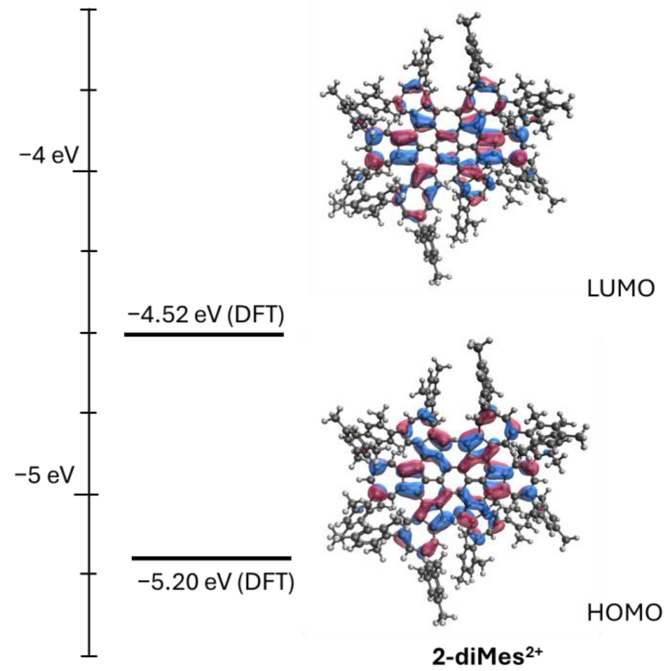

Figure S50. Molecular frontier orbitals of [**2-diMes**]$^{2+}$, B3LYP/6-31g(d,p).

Table S6. Calculated electronic transitions for **1-diMes**, B3LYP/6-31g(d,p).

Transition	Energy	Wavelength	Oscillator strength	Contribution
**$S_0 \rightarrow S_1$**	**1.1731 eV**	**1056.89 nm**	**f= 0.00000**	**H -> L 93.6%**
**$S_0 \rightarrow S_2$**	1.2106 eV	1024.15 nm	f= 0.00040	H-2 -> L 46.6%, H-1 -> L+1 36.2%, H -> L+2 16.6%
**$S_0 \rightarrow S_3$**	1.2115 eV	1023.39 nm	f= 0.00040	H -> L+1 93.0%
**$S_0 \rightarrow S_4$**	1.3528 eV	916.50 nm	f= 0.03550	H-1 -> L 82.9%, H-2 -> L+1 13.8%
**$S_0 \rightarrow S_5$**	1.4170 eV	874.98 nm	f= 0.13090	H-2 -> L+1 75.5%, H-1 -> L 10.2%
**$S_0 \rightarrow S_6$**	1.4180 eV	874.36 nm	f= 0.17660	H-1 -> L+1 47.5%, H-2 -> L 37.5%, H -> L+8 5.5%
**$S_0 \rightarrow S_7$**	1.6587 eV	747.48 nm	f= 0.00050	H -> L+2 82.6%, H-2 -> L 9.0%, H-1 -> L+1 7.8%
**$S_0 \rightarrow S_8$**	1.7162 eV	722.43 nm	f= 0.00000	H-1 -> L+2 87.4%, H -> L+1 5.4%
**$S_0 \rightarrow S_9$**	1.7219 eV	720.04 nm	f= 0.00000	H-2 -> L+2 87.7%, H -> L 5.2%

Table S7. Calculated electronic transitions for **2-diMes**, B3LYP/6-31g(d,p).

Transition	Energy	Wavelength	Oscillator strength	Contribution
**$S_0 \rightarrow S_1$**	**1.1385 eV**	**1089.01 nm**	**f= 0.24130**	**H -> L 96.3%**
**$S_0 \rightarrow S_2$**	1.1953 eV	1037.26 nm	f= 0.02190	H -> L+1 88.3%, H-1 -> L+2 7.8%
**$S_0 \rightarrow S_3$**	1.3013 eV	952.77 nm	f= 0.00000	H-1 -> L 96.2%
**$S_0 \rightarrow S_4$**	1.3379 eV	926.71 nm	f= 0.00270	H -> L+2 48.2%, H-1 -> L+1 47.0%
**$S_0 \rightarrow S_5$**	1.6271 eV	761.99 nm	f= 0.00590	H-1 -> L+1 48.1%, H -> L+2 46.2%
**$S_0 \rightarrow S_6$**	1.6799 eV	738.05 nm	f= 0.04930	H-2 -> L 56.0%, H -> L+3 18.1%, H-1 -> L+2 16.3%
**$S_0 \rightarrow S_7$**	1.7924 eV	691.72 nm	f= 0.00230	H-3 -> L 60.5%, H -> L+6 18.0%, H-1 -> L+3 11.9%
**$S_0 \rightarrow S_8$**	1.8134 eV	683.71 nm	f= 0.00650	H-1 -> L+2 74.0%, H-2 -> L 13.5%, H -> L+1 5.6%, H -> L+3 5.3%
**$S_0 \rightarrow S_9$**	1.8360 eV	675.30 nm	f= 0.05210	H -> L+3 65.4%, H-2 -> L 26.1

Table S8. Calculated electronic transitions for **[2-diMes]$^{•+}$**, UB3LYP/6-31g(d,p).

Transition	Energy	Wavelength	Oscillator strength	Contribution
**$D_0 \rightarrow D_1$**	**0.3894 eV**	**3183.98 nm**	**f= 0.10100**	**Hb -> Lb 95.2%, Ha -> La 5.2%**
**$D_0 \rightarrow D_2$**	0.8560 eV	1448.41 nm	f= 0.01380	Hb-1 -> Lb 93.0%
**$D_0 \rightarrow D_3$**	0.9600 eV	1291.50 nm	f= 0.04730	Hb-2 -> Lb 88.1%
**$D_0 \rightarrow D_4$**	1.0027 eV	1236.50 nm	f= 0.00000	Hb -> Lb+1 60.2%, Hb-3 -> Lb 24.3%, Ha-1 -> La 10.4%
**$D_0 \rightarrow D_5$**	1.0765 eV	1151.73 nm	f= 0.23370	Ha -> La 86.1%
**$D_0 \rightarrow D_6$**	1.0897 eV	1137.78 nm	f= 0.00000	Hb-3 -> Lb 73.2%, Hb -> Lb+1 21.7%
**$D_0 \rightarrow D_7$**	1.1155 eV	1111.47 nm	f= 0.01600	Ha -> La+1 76.6%, Ha-1 -> La+2 11.1%, Hb-2 -> Lb 5.1%
**$D_0 \rightarrow D_8$**	1.1322 eV	1095.07 nm	f= 0.00160	Hb -> Lb+2 68.6%, Ha-1 -> La+1 12.6%, Ha -> La+2 10.3%
**$D_0 \rightarrow D_9$**	1.2822 eV	966.96 nm	f= 0.00000	Ha -> La+2 36.4%, Ha-1 -> La+1 32.9%, Hb -> Lb+2 26.0%

Table S9. Calculated electronic transitions for **[2-diMes]$^{2+}$**, B3LYP/6-31g(d,p).

Transition	Energy	Wavelength	Oscillator strength	Contribution
**$S_0 \rightarrow S_1$**	**0.6468 eV**	**1916.89 nm**	**f= 0.33790**	**H -> L 112.4%, H <- L -14.8%**
**$S_0 \rightarrow S_2$**	0.8488 eV	1460.70 nm	f= 0.02410	H-1 -> L 98.1%
**$S_0 \rightarrow S_3$**	0.9710 eV	1276.87 nm	f= 0.09030	H-2 -> L 96.3%
**$S_0 \rightarrow S_4$**	0.9856 eV	1257.96 nm	f= 0.00000	H-3 -> L 99.5%
**$S_0 \rightarrow S_5$**	1.1968 eV	1035.96 nm	f= 0.00070	H -> L+2 99.2%
**$S_0 \rightarrow S_6$**	1.2393 eV	1000.44 nm	f= 0.00000	H -> L+1 97.8%
**$S_0 \rightarrow S_7$**	1.4593 eV	849.61 nm	f= 0.00160	H-4 -> L 81.1%, H-5 -> L 14.4%
**$S_0 \rightarrow S_8$**	1.4605 eV	848.92 nm	f= 0.00140	H-5 -> L 81.0%, H-4 -> L 16.6%
**$S_0 \rightarrow S_9$**	1.4633 eV	847.29 nm	f= 0.00040	H-6 -> L 97.8%

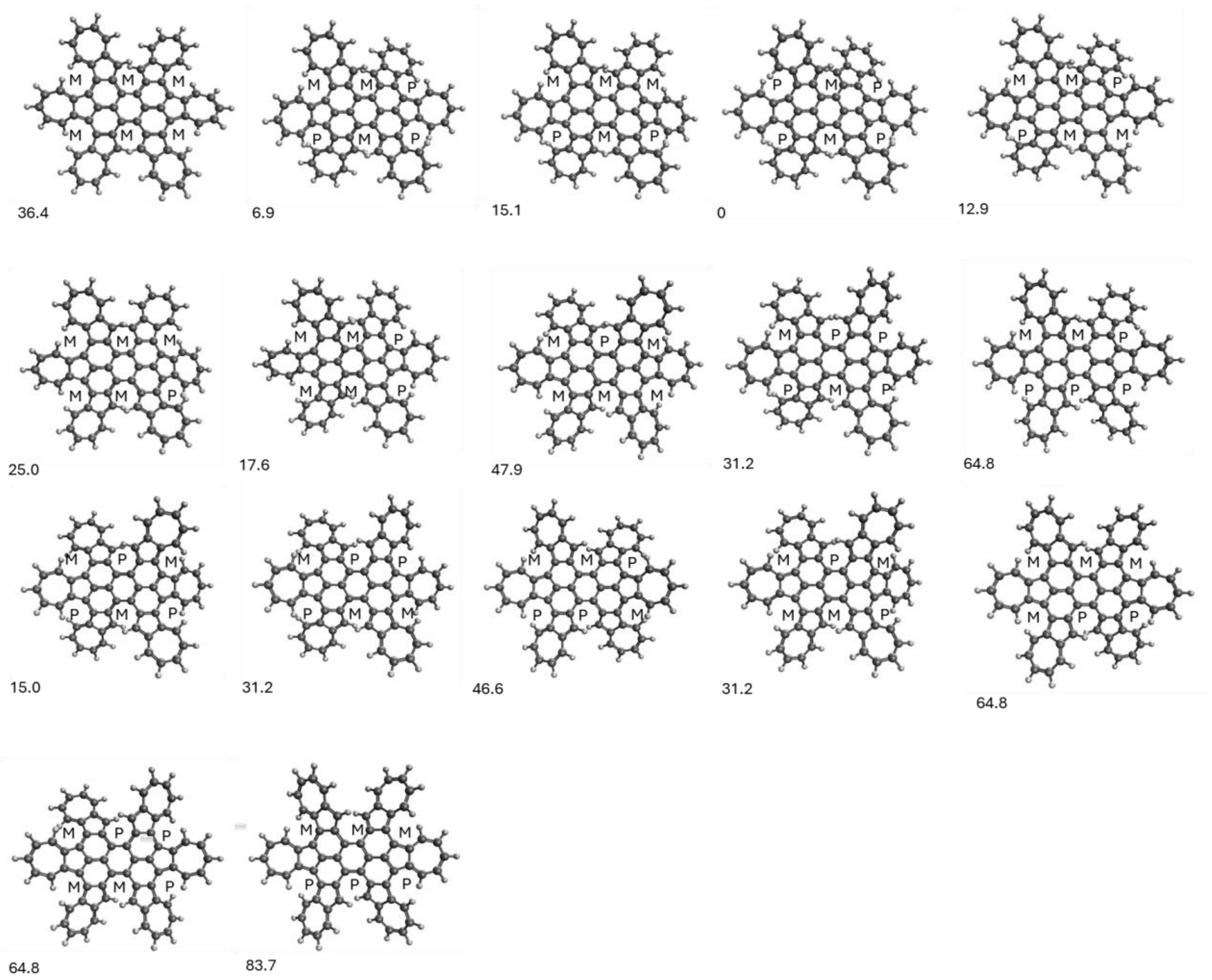

Figure S51. Relative energies of **2-diMes** conformers (kJ/mol), B3LYP/6-31g(d,p). Only one enantiomer from pair of enantiomers calculated.

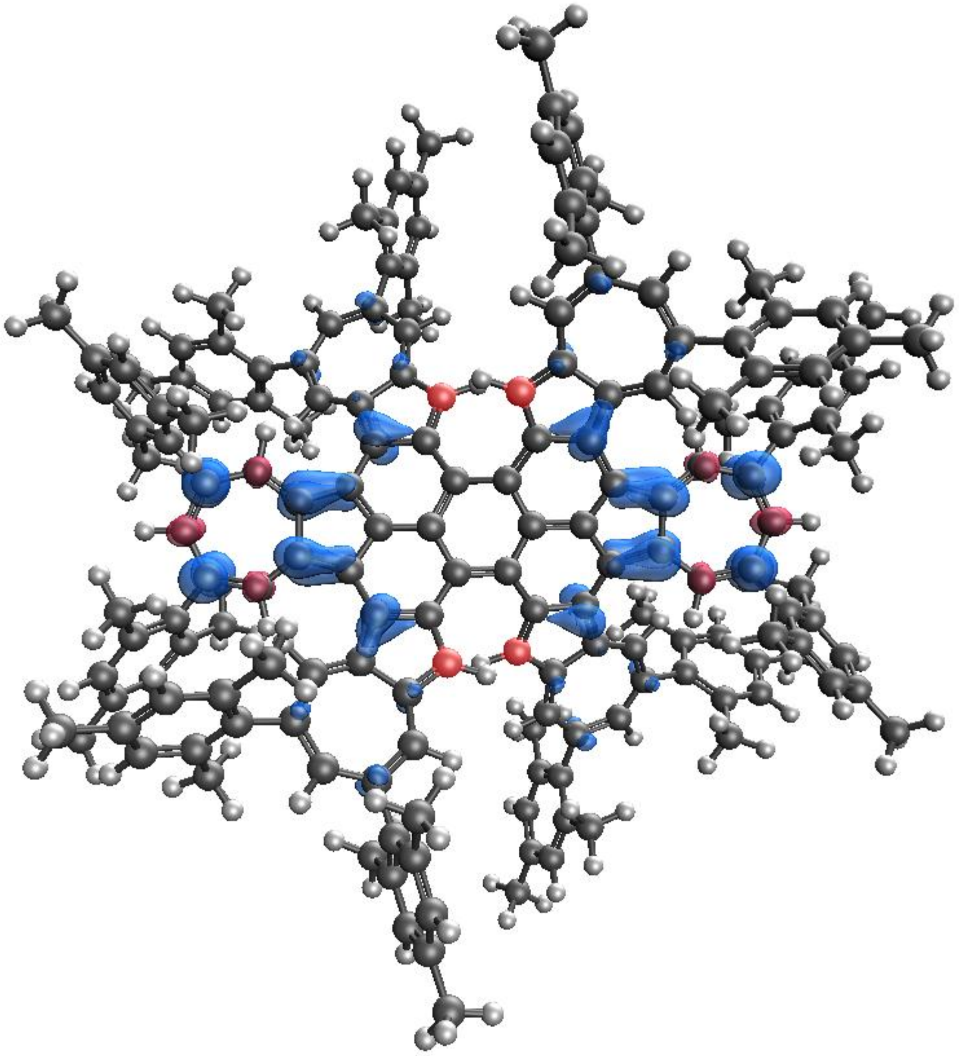

Figure S52. Spin density of intermediate radical cation **[2-diMes]$^{+\bullet}$** possibly leading to more oxidated species from **2-diMes** (UB3LYP/6-31g(d,p)). There is no positive spin density on relevant carbon atoms (marked with red).

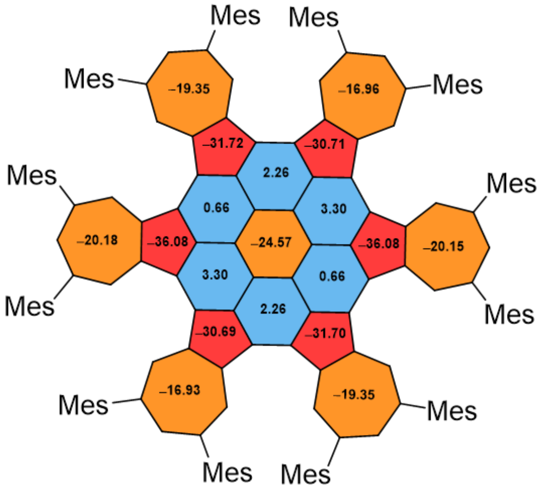

1-diMes

Figure S53. NICS(1)$_{zz}$ values of **1-diMes**, B3LYP6-31G(d,p).

DFT-optimized cartesian coordinates

Optimized geometry of 1-diMes, B3LYP/6-31g(d,p)

Energy: -6725.514412 a.u.

C	-9.17065	-3.09933	0.06796
C	-4.93194	0.66064	0.01986
C	-8.31450	-2.43738	-0.83841
C	-4.90393	-0.85734	-0.03841
C	-2.77381	-2.53931	0.11494
C	-3.00265	-3.91648	0.44380
C	-10.11934	-4.40692	-1.77029
C	-2.79694	-0.05766	-0.03256
C	-1.35320	-2.45004	-0.02491
C	1.39514	0.02926	-0.03981
C	-8.36173	-2.76408	-2.21269
C	-4.20860	-4.46836	0.87199
H	-5.04676	-3.77978	0.88287
C	0.72265	-1.19378	-0.01835
C	-3.58664	1.13393	-0.07680
C	-3.53911	-1.27959	0.02727
C	-10.05723	-4.06847	-0.41614
H	-10.71377	-4.57481	0.28781
C	-1.39553	-0.02865	-0.03999
C	-6.00666	-1.66279	-0.31209
H	-5.77111	-2.69610	-0.54989
C	-4.52749	-5.75688	1.34254
C	-4.39239	4.28541	-0.92314
H	-5.19632	3.55691	-0.94016
C	-7.38004	-1.35717	-0.36425
C	-1.45179	2.39247	-0.04009
C	2.79654	0.05828	-0.03223
C	-1.66398	-4.63755	0.41055
C	-2.87509	2.42313	-0.16842
C	-6.05322	1.42808	0.32386
H	-5.84594	2.46986	0.55138
C	-0.72302	1.19439	-0.01972
C	-0.67405	-1.22309	-0.05895
C	-9.26235	-3.74068	-2.65132
C	-1.40907	-5.93633	0.84747
H	-0.36046	-6.21669	0.83562
C	-1.62012	-8.22606	1.78904
C	1.35276	2.45073	-0.02726
C	-3.16385	3.79004	-0.49050
C	-6.37786	-5.74097	3.06503
C	0.67368	1.22369	-0.06020
C	3.58634	-1.13332	-0.07522
C	-0.65688	-3.69278	0.05240
C	-7.41250	1.07473	0.42029
C	2.87480	-2.42269	-0.16561
C	-9.14993	-2.79160	1.54951
H	-8.12794	-2.73481	1.93536
H	-9.67965	-3.56500	2.11068
H	-9.62890	-1.83199	1.77617
C	-7.97425	-0.15180	0.03766
H	-9.06143	-0.17208	0.05547
C	-2.25924	-6.93344	1.35192
C	-4.76944	5.55814	-1.39325
C	3.53859	1.28035	0.02631
C	1.45145	-2.39188	-0.03750
C	-5.95657	-6.01903	1.74725
C	0.65641	3.69354	0.04911
C	-11.06381	-5.47790	-2.26360
H	-10.58500	-6.46515	-2.25064
H	-11.38233	-5.28733	-3.29283
H	-11.95749	-5.54348	-1.63596
C	4.93164	-0.65970	0.02060

C	-3.64638	-6.83098	1.53327
H	-4.11459	-7.72825	1.93287
C	-0.96895	-8.29942	3.04035
C	-8.36774	2.12225	0.92526
C	4.20794	4.46961	0.86897
H	5.04633	3.78130	0.87939
C	2.77330	2.54013	0.11282
C	3.16354	-3.78994	-0.48646
C	-0.47637	-10.66402	2.65241
C	-6.85620	-6.60377	0.83133
C	0.80869	-3.66343	-0.10632
C	4.39236	-4.28617	-0.91732
H	5.19677	-3.55818	-0.93359
C	3.00201	3.91747	0.44114
C	-6.20439	5.74730	-1.81711
C	6.05322	-1.42646	0.32518
H	5.84631	-2.46796	0.55429
C	1.66329	4.63847	0.40743
C	4.90340	0.85823	-0.03932
C	-7.13934	6.31118	-0.92428
C	7.41255	-1.07274	0.42010
C	-7.48199	-2.06061	-3.22366
H	-7.56033	-0.97184	-3.13714
H	-7.76328	-2.34198	-4.24174
H	-6.42332	-2.30985	-3.09159
C	-1.85744	4.56810	-0.45615
C	-0.80911	3.66398	-0.10983
C	-7.68618	-6.06338	3.44463
H	-8.00228	-5.85557	4.46458
C	-5.43673	-5.12549	4.07676
C	4.52646	5.75805	1.34005
C	-6.59927	5.41749	-3.13169
C	1.85699	-4.56786	-0.45199
C	-8.37742	2.45183	2.29956
C	5.95569	6.02058	1.74394
C	6.60390	-5.41880	-3.12005
C	-8.15369	-6.91382	1.25373
C	-9.27031	2.75954	0.04662
C	6.85544	6.60264	0.82646
C	-8.58951	-6.65452	2.55663
C	4.76921	-5.55934	-1.38650
C	-6.43886	-6.90754	-0.59017
H	-6.09048	-6.00752	-1.10807
H	-7.27375	-7.32210	-1.16062
H	-5.61482	-7.62883	-0.62654
C	-1.12443	-10.56911	1.41793
H	-1.18815	-11.44927	0.78218
C	-8.85653	6.23663	-2.66488
C	6.00578	1.66362	-0.31463
C	-9.28922	3.40476	2.76638
H	-9.29337	3.65055	3.82612
C	-6.75091	6.67207	0.49207
C	-0.41011	-9.51585	3.44759
H	0.08173	-9.56817	4.41652
C	6.20463	-5.75095	-1.80759
C	-3.94000	6.67549	-1.56698
H	-4.44760	7.55287	-1.96271
C	-1.69409	-9.37158	0.96933
C	7.37912	1.35811	-0.36774
C	-10.19649	4.04139	1.91432
C	5.62990	-4.81686	-4.10859
H	4.78106	-5.48464	-4.29383
H	6.11907	-4.62137	-5.06619
H	5.21070	-3.87259	-3.74511
C	-8.44609	6.54877	-1.36565
H	-9.16012	6.99045	-0.67427
C	7.97377	0.15326	0.03501
C	-2.56062	6.84290	-1.37395
C	1.40796	5.93701	0.84493
H	0.35931	6.21721	0.83243

C	-1.35290	8.30063	-3.04743	H	-8.27343	2.44380	-1.85457
C	7.92383	-5.66697	-3.51432	H	-9.86538	3.20458	-1.97717
H	8.22500	-5.41102	-4.52782	C	7.68572	6.06776	3.44081
C	-7.91723	5.66811	-3.53048	H	8.00198	5.86201	4.46113
C	3.64482	6.83137	1.53254	C	2.55945	-6.84269	-1.37041
H	4.11265	7.72822	1.93351	C	8.31309	2.43771	-0.84426
C	1.66317	-5.88173	-0.87511	C	9.17008	3.10091	0.06039
H	0.62901	-6.21138	-0.86016	C	10.20040	-4.03445	1.91669
C	6.37711	5.74535	3.06229	C	1.98160	-8.17012	-1.78900
C	8.15323	6.91289	1.24784	C	6.43792	6.90358	-0.59558
H	8.83925	7.36944	0.53800	H	6.08855	6.00277	-1.11143
C	8.36859	-2.11914	0.92573	H	7.27300	7.31613	-1.16722
C	-1.98242	8.17111	-1.78963	H	5.61453	7.62555	-0.63325
C	-10.16562	3.70634	0.55812	C	0.15522	-11.96009	3.10417
H	-10.85204	4.19981	-0.12619	H	0.11654	-12.06580	4.19254
C	-1.23233	7.12515	-3.99210	H	1.21189	-12.00949	2.81250
H	-0.61583	6.32358	-3.57089	H	-0.34480	-12.82525	2.65901
H	-0.77923	7.43277	-4.93792	C	-5.62038	4.82294	-4.11984
H	-2.21033	6.68363	-4.21250	H	-5.19689	3.88028	-3.75726
C	8.58921	6.65626	2.55121	H	-4.77472	5.49545	-4.30258
C	-1.66396	5.88200	-0.87922	H	-6.10699	4.62653	-5.07856
C	2.25757	6.93351	1.35152	C	-0.84885	9.54779	-3.43205
C	-0.88420	-7.09811	3.95619	H	-0.37314	9.64307	-4.40577
H	-0.29139	-6.28875	3.51617	C	10.00118	6.98426	2.97767
H	-0.42232	-7.36916	4.90893	H	10.68789	6.15907	2.75037
H	-1.87497	-6.68072	4.16594	H	10.06116	7.16749	4.05447
C	-7.43908	1.78221	3.28038	H	10.37976	7.87010	2.45880
H	-6.39476	2.06690	3.11091	C	-10.00109	-6.98271	2.98425
H	-7.48330	0.69118	3.19850	H	-10.06100	-7.16130	4.06182
H	-7.69417	2.05675	4.30718	H	-10.37824	-7.87141	2.46921
C	-2.09047	9.29235	-0.94119	H	-10.68902	-6.15960	2.75315
C	3.93914	-6.67592	-1.56194	C	9.27328	-2.75453	0.04805
H	4.44662	-7.55330	-1.95780	C	-11.19642	5.04020	2.44823
C	-9.28388	2.45484	-1.43553	H	-12.12326	4.54381	2.76230
H	-9.72681	1.47511	-1.64866	H	-11.46738	5.77999	1.68914

H	-10.80411	5.57263	3.31977
C	7.13802	-6.31173	-0.91102
C	-2.37345	-9.32916	-0.38133
H	-2.21643	-10.26451	-0.92400
H	-1.98940	-8.51109	-0.99901
H	-3.45462	-9.17484	-0.28833
C	8.37744	-2.44892	2.30016
C	-10.27851	6.48284	-3.11272
H	-10.91442	5.61030	-2.91625
H	-10.33026	6.68402	-4.18688
H	-10.72268	7.33169	-2.58439
C	10.17008	-3.69954	0.56059
H	10.85823	-4.19129	-0.12322
C	5.43582	5.13268	4.07557
H	4.57243	5.78033	4.26577
H	5.94543	4.96459	5.02771
H	5.03642	4.17261	3.73202
C	1.57184	-10.52186	-1.37070
H	1.65724	-11.38358	-0.71270
C	1.34315	-8.29457	-3.04257
C	11.20189	-5.03090	2.45210
H	11.48763	-5.76062	1.68875
H	10.80361	-5.57545	3.31345
H	12.12085	-4.53052	2.78254
C	-2.74867	9.18917	0.41650
H	-3.81667	8.95746	0.33444
H	-2.65226	10.12806	0.96707
H	-2.29906	8.39535	1.02182
C	2.09169	-9.29412	-0.94395
C	8.85935	-6.23992	-2.64764
C	9.15054	2.79541	1.54242
C	10.05626	4.06935	-0.42589
H	10.71343	4.57666	0.27678
C	8.44660	-6.54699	-1.34775
H	9.16116	-6.97973	-0.65132
C	10.11714	4.40589	-1.78056
C	0.94366	-10.66906	-2.61046
C	9.29069	-3.39984	2.76787
H	9.29427	-3.64565	3.82761
C	8.35906	2.76249	-2.21904
C	2.74858	-9.19344	0.41457
H	3.81197	-8.94134	0.33483
H	2.67019	-10.14013	0.95484
H	2.28477	-8.41465	1.02886
C	6.74787	-6.66425	0.50698
H	6.00429	-7.46903	0.53444
H	6.30404	-5.80954	1.02781
H	7.61860	-6.99364	1.07928
C	10.26565	-6.54319	-3.10982
H	10.98202	-6.47363	-2.28568
H	10.58488	-5.85631	-3.89918
H	10.33807	-7.55995	-3.51599
C	-1.57477	10.52231	-1.36737
H	-1.66448	11.38364	-0.70944
C	-0.94880	10.67282	-2.60753
C	9.28918	-2.44976	-1.43411
H	9.75024	-1.47837	-1.64758
H	8.27834	-2.41950	-1.85107
H	9.85512	-3.21038	-1.97688
C	0.83463	-9.53996	-3.42788
H	0.34587	-9.62971	-4.39560
C	7.43692	-1.78114	3.28017
H	7.69208	-2.05474	4.30721
H	6.39335	-2.06832	3.11031
H	7.47864	-0.69005	3.19795
C	0.42331	-12.01218	-3.06672
H	1.18043	-12.55353	-3.64784
H	-0.45748	-11.90391	-3.70678
H	0.15290	-12.64641	-2.21738
C	9.25929	3.73846	-2.65986

H	9.29665	3.97555	-3.72094
C	1.21113	-7.11437	-3.97987
H	2.18557	-6.66557	-4.20118
H	0.59102	-6.31899	-3.55224
H	0.75665	-7.41962	-4.92579
C	11.06122	5.47611	-2.27629
H	10.58200	6.46318	-2.26577
H	11.37993	5.28322	-3.30503
H	11.95480	5.54352	-1.64871
C	7.47837	2.05762	-3.22820
H	6.41982	2.30699	-3.09545
H	7.55685	0.96897	-3.14025
H	7.75867	2.33760	-4.24693
C	-0.37576	12.00343	-3.03649
H	0.69285	12.07129	-2.79676
H	-0.87561	12.83540	-2.53206
H	-0.47251	12.15104	-4.11649
H	-0.10639	9.55271	4.41203
C	0.39693	9.50665	3.44870
C	0.96025	8.29270	3.04014
C	0.86583	7.08613	3.94814
C	1.61861	8.22502	1.79228
C	1.69257	9.37309	0.97602
C	2.36874	9.33358	-0.37633
C	1.11851	10.56801	1.42587
H	1.17632	11.44832	0.78985
C	0.47026	10.65927	2.66060
C	-0.10917	11.96875	3.14269
H	0.40251	7.35391	4.90110
H	1.85378	6.66241	4.15834
H	0.27013	6.28245	3.50163
H	1.96813	8.53038	-1.00316
H	3.44679	9.15761	-0.28735
H	2.22899	10.27838	-0.90751
H	-0.99808	11.81003	3.76067
H	-0.38583	12.61650	2.30568
H	0.61521	12.52075	3.75471
H	-9.30068	-3.97926	-3.71203
H	-5.03930	-4.16510	3.73180
H	-4.57206	-5.77142	4.26689
H	-5.94580	-4.95718	5.02914
H	-8.83960	-7.37243	0.54509
H	5.76980	2.69663	-0.55337
H	-6.00912	7.47864	0.51621
H	-6.30556	5.82084	1.01728
H	-7.62280	7.00218	1.06218
H	9.06096	0.17354	0.05139
H	-8.21314	5.42042	-4.54759
H	-0.62991	6.21204	-0.86422
H	9.68255	3.56855	2.10177
H	9.62770	1.83513	1.76997
H	8.12887	2.74140	1.92955

Optimized geometry of 2-diMes (the lowest energy conformation), B3LYP/6-31g(d,p)

Energy: -6723.654230 a.u.

C	-9.28588	-2.81898	0.78752
C	-4.96290	0.70958	-0.18272
C	-8.36320	-2.47229	-0.22386
C	-4.95610	-0.74984	0.17467
C	-2.89395	-2.30208	0.82103
C	-3.27046	-3.44756	1.60127
C	-10.15306	-4.63238	-0.60990
C	-2.80455	-0.01112	-0.00802
C	-1.46911	-2.37192	0.61613
C	1.38071	0.00717	-0.00963
C	-8.34122	-3.20857	-1.43158
C	-4.46580	-3.65437	2.27164
H	-5.21070	-2.87286	2.16011
C	0.70704	-1.24343	-0.14483
C	-3.60509	1.10377	-0.37870
C	-3.59435	-1.13251	0.36569
C	-10.16165	-3.89088	0.57376
H	-10.86673	-4.15346	1.35930
C	-1.38069	-0.00521	-0.00977
C	-6.05536	-1.59916	0.11550
H	-5.82433	-2.65854	0.18628
C	-4.86453	-4.71579	3.12375
C	-4.50391	3.62260	-2.27759
H	-5.24288	2.83579	-2.16389
C	-7.42523	-1.30943	-0.04684
C	-1.49094	2.36063	-0.63494
C	2.80457	0.01296	-0.00770
C	-2.03601	-4.27406	1.76151
C	-2.91581	2.27943	-0.83570
C	-6.06933	1.54927	-0.11915
H	-5.84790	2.61063	-0.19073
C	-0.70698	1.24532	-0.14595
C	-0.69577	-1.24978	0.12567
C	-9.23511	-4.27074	-1.60089
C	-1.89863	-5.52205	2.38970
H	-0.90375	-5.95840	2.31162
C	-2.37943	-7.61336	3.62617
C	1.46897	2.37442	0.61436
C	-3.30391	3.42285	-1.61330
C	-6.37391	-3.75192	4.90505
C	0.69578	1.25186	0.12469
C	3.60529	-1.10225	-0.37730
C	-0.98489	-3.58277	1.17314
H	0.04419	-3.90960	1.18485
C	-7.43592	1.24751	0.04868
C	2.91613	-2.27847	-0.83337
C	-9.35244	-2.07015	2.10089
H	-8.35576	-1.85925	2.49832
H	-9.89498	-2.65481	2.84707
H	-9.86576	-1.10686	1.99895
C	-8.00603	-0.03345	0.00184
H	-9.09305	-0.03799	0.00372
C	-2.82806	-6.27365	3.10274
C	-4.91456	4.68331	-3.12489
C	3.59416	1.13472	0.36526
C	1.49111	-2.35910	-0.63280
C	-6.21423	-4.59887	3.78509
C	0.98468	3.58569	1.17037
H	-0.04439	3.91259	1.18159
C	-11.08845	-5.80238	-0.80650
H	-10.60312	-6.74836	-0.53535
H	-11.40384	-5.89100	-1.85066
H	-11.98430	-5.70698	-0.18617
C	4.96300	-0.70757	-0.18155

C	-4.14158	-5.86937	3.43359
H	-4.67396	-6.57362	4.06975
C	-1.86257	-7.72884	4.93389
C	-8.38431	2.40128	0.22921
C	4.46529	3.65760	2.26990
H	5.21016	2.87598	2.15897
C	2.89374	2.30464	0.81963
C	3.30414	-3.42315	-1.60935
C	-1.54508	-10.13159	4.60218
C	-7.30269	-5.37380	3.33274
C	1.01775	-3.57401	-1.18971
H	-0.00893	-3.90825	-1.20466
C	4.50454	-3.62519	-2.27245
H	5.24432	-2.83907	-2.15936
C	3.27012	3.45054	1.59928
C	-6.26817	4.56037	-3.77720
C	6.06978	-1.54675	-0.11748
H	5.84881	-2.60826	-0.18811
C	2.03569	4.27727	1.75865
C	4.95594	0.75214	0.17461
C	-7.35781	5.32711	-3.31413
C	7.43631	-1.24426	0.04938
C	-7.39574	-2.85374	-2.55925
H	-7.43877	-1.78656	-2.80002
H	-7.64536	-3.41684	-3.46226
H	-6.35271	-3.07686	-2.30897
C	-2.07629	4.25887	-1.77733
C	-1.01789	3.57527	-1.19301
H	0.00866	3.90992	-1.20795
C	-7.61253	-3.70483	5.55353
H	-7.72487	-3.05969	6.42226
C	-5.22529	-2.92163	5.43492
C	4.86381	4.71935	3.12164
C	-6.43081	3.71691	-4.89943
C	2.07605	-4.25877	-1.77282

C	-8.36212	3.14149	1.43459
C	6.21335	4.60274	3.78335
C	6.43629	-3.72602	-4.89070
C	-8.52388	-5.29789	4.01411
C	-9.31209	2.73993	-0.78012
C	7.30181	5.37772	3.33102
C	-8.70140	-4.47180	5.12680
C	4.91468	-4.68784	-3.11762
C	-7.17828	-6.28578	2.13182
H	-6.70903	-5.77579	1.28494
H	-8.16157	-6.63922	1.81168
H	-6.56412	-7.16698	2.35074
C	-2.06374	-9.99542	3.31109
H	-2.14899	-10.87532	2.67708
C	-8.76384	4.42441	-5.10209
C	6.05486	1.60185	0.11481
C	-9.26312	4.19737	1.60484
H	-9.24231	4.75816	2.53686
C	-7.23024	6.23568	-2.11100
C	-1.45601	-8.98533	5.39754
H	-1.05948	-9.06861	6.40712
C	6.26892	-4.56986	-3.76962
C	-4.20080	5.84204	-3.43696
H	-4.74152	6.54403	-4.06855
C	-2.48082	-8.75798	2.80757
C	7.42480	1.31261	-0.04760
C	-10.19210	4.54567	0.61923
C	5.29221	-2.89476	-5.42880
H	4.41791	-3.51422	-5.65604
H	5.58756	-2.37620	-6.34445
H	4.96041	-2.13810	-4.70945
C	-8.58339	5.24675	-3.98721
H	-9.41803	5.84422	-3.62722
C	8.00599	0.03686	0.00149
C	-2.88804	6.25422	-3.11311

C	1.89824	5.52559	2.38611
H	0.90343	5.96202	2.30745
C	-1.93984	7.71681	-4.94735
C	7.68033	-3.67822	-5.52878
H	7.80041	-3.03059	-6.39461
C	-7.67353	3.66542	-5.53954
C	4.14080	5.87309	3.43078
H	4.67294	6.57754	4.06692
C	1.94900	-5.50865	-2.39919
H	0.95616	-5.95036	-2.32531
C	6.37292	3.75609	4.90353
C	8.52287	5.30214	4.01263
H	9.35648	5.90610	3.66114
C	8.38546	-2.39754	0.22867
C	-2.45061	7.59721	-3.63761
C	-10.19536	3.80556	-0.56530
H	-10.90020	4.06597	-1.35174
C	-1.81354	6.51154	-5.85250
H	-1.19825	5.72804	-5.39740
H	-1.35862	6.78767	-6.80714
H	-2.78924	6.05972	-6.06390
C	8.70029	4.47630	5.12555
C	-1.95023	5.50811	-2.40529
C	2.82751	6.27740	3.09918
C	-1.74042	-6.52223	5.83783
H	-1.13254	-5.73481	5.37954
H	-1.27934	-6.79472	6.79056
H	-2.71821	-6.07693	6.05341
C	-7.40385	2.80198	2.55618
H	-6.36538	3.03683	2.29780
H	-7.43240	1.73512	2.80041
H	-7.65379	3.36470	3.45933
C	-2.55623	8.74068	-2.81794
C	4.19988	-5.84638	-3.42838
H	4.74051	-6.54992	-4.05832
C	-9.37158	1.99354	-2.09522
H	-9.87636	1.02555	-1.99516
H	-8.37316	1.79222	-2.49330
H	-9.91951	2.57491	-2.84004
C	7.61144	3.70930	5.55225
H	7.72370	3.06438	6.42115
C	2.88649	-6.25676	-3.10527
C	8.36229	2.47571	-0.22549
C	9.28526	2.82314	0.78541
C	10.19510	-4.54078	0.61559
C	2.44868	-7.59991	-3.62904
C	7.17751	6.28921	2.12971
H	6.71056	5.77805	1.28224
H	8.16062	6.64442	1.81097
H	6.56126	7.16925	2.34738
C	-1.07047	-11.47196	5.11291
H	-1.17868	-11.54741	6.19895
H	-0.00991	-11.63130	4.88099
H	-1.62841	-12.29460	4.65586
C	-5.28111	2.89528	-5.44029
H	-4.94278	2.14051	-4.72200
H	-4.41167	3.52134	-5.66813
H	-5.57389	2.37528	-6.35595
C	-1.54341	8.97621	-5.41189
H	-1.15155	9.06266	-6.42303
C	10.03307	4.39334	5.83246
H	10.62785	3.54920	5.46135
H	9.90581	4.24722	6.90952
H	10.62447	5.30052	5.67854
C	-10.03428	-4.38851	5.83349
H	-9.90715	-4.24374	6.91075
H	-10.62642	-5.29500	5.67839
H	-10.62823	-3.54341	5.46324
C	9.31228	-2.73493	-0.78188
C	-11.17723	5.66914	0.84255

H	-12.05032	5.32353	1.41017
H	-11.54472	6.07322	-0.10510
H	-10.72781	6.48852	1.41202
C	7.35636	-5.33802	-3.30334
C	-3.03635	-8.66978	1.40333
H	-3.07287	-9.65730	0.93639
H	-2.42686	-8.01856	0.76725
H	-4.04977	-8.25371	1.39506
C	8.36498	-3.13839	1.43371
C	-10.10087	4.33654	-5.80038
H	-10.68271	3.47950	-5.43850
H	-9.97999	4.20887	-6.88055
H	-10.70172	5.23408	-5.62793
C	10.19652	-3.80011	-0.56855
H	10.90063	-4.05964	-1.35595
C	5.22433	2.92575	5.43337
H	4.34900	3.54557	5.65557
H	5.51323	2.40566	6.35021
H	4.89675	2.17030	4.71080
C	2.13661	-9.98179	-3.30909
H	2.21455	-10.85870	-2.67003
C	1.93417	-7.71917	-4.93729
C	11.18122	-5.66370	0.83735
H	11.55001	-6.06530	-0.11084
H	10.73216	-6.48484	1.40459
H	12.05339	-5.31844	1.40658
C	-3.10555	8.64802	-1.41155
H	-4.11660	8.22632	-1.39959
H	-3.14568	9.63487	-0.94348
H	-2.48987	7.99958	-0.77860
C	2.54927	-8.74228	-2.80714
C	8.76589	-4.44514	-5.09344
C	9.35260	2.07483	2.09904
C	10.16060	3.89521	0.57086
H	10.86590	4.15835	1.35602

C	8.58317	-5.26156	-3.97443
H	9.41771	-5.85534	-3.60820
C	10.15134	4.63619	-0.61314
C	1.62793	-10.12312	-4.60366
C	9.26691	-4.19367	1.60244
H	9.24754	-4.75497	2.53420
C	8.33966	3.21144	-1.43352
C	3.09191	-8.64802	-1.39826
H	4.10390	-8.22870	-1.38278
H	3.12781	-9.63405	-0.92814
H	2.47505	-7.99687	-0.76927
C	7.22674	-6.23872	-2.09454
H	6.62654	-7.12965	-2.31315
H	6.73957	-5.72641	-1.25935
H	8.20974	-6.57666	-1.75737
C	10.08585	-4.40966	-5.82799
H	10.91446	-4.70288	-5.17693
H	10.29806	-3.41126	-6.22250
H	10.08250	-5.09936	-6.68135
C	-2.14930	9.98111	-3.32240
H	-2.23771	10.86007	-2.68752
C	-1.63673	10.12140	-4.61549
C	9.37002	-1.98761	-2.09652
H	9.88374	-1.02407	-1.99814
H	8.37095	-1.77711	-2.48809
H	9.90785	-2.57252	-2.84578
C	1.53196	-8.97771	-5.39934
H	1.13062	-9.06274	-6.40686
C	7.40760	-2.80010	2.55642
H	7.65889	-3.36299	3.45909
H	6.36904	-3.03565	2.29901
H	7.43556	-1.73331	2.80106
C	1.21809	-11.47673	-5.13513
H	2.06448	-11.98878	-5.60988
H	0.42958	-11.38868	-5.88834

H	0.85470	-12.12770	-4.33439
C	9.23316	4.27382	-1.60362
H	9.21682	4.82678	-2.54039
C	1.80247	-6.51315	-5.84066
H	2.77652	-6.05799	-6.05246
H	1.18517	-5.73205	-5.38408
H	1.34750	-6.78963	-6.79517
C	11.08629	5.80641	-0.81049
H	10.60164	6.75206	-0.53697
H	11.39923	5.89632	-1.85527
H	11.98360	5.71023	-0.19237
C	7.39398	2.85576	-2.56076
H	6.35090	3.07838	-2.31020
H	7.43752	1.78853	-2.80121
H	7.64299	3.41871	-3.46402
C	-1.17293	11.46512	-5.12733
H	-0.11247	11.63117	-4.89973
H	-1.73427	12.28386	-4.66746
H	-1.28614	11.54052	-6.21287
H	1.04047	9.07023	6.39533
C	1.44698	8.98842	5.38962
C	1.85830	7.73261	4.92823
C	1.72915	6.52502	5.82989
C	2.37953	7.61747	3.62225
C	2.47756	8.76119	2.80194
C	3.02720	8.67140	1.39548
C	2.05574	9.99792	3.30318
H	2.13183	10.87590	2.66536
C	1.54033	10.13517	4.59551
C	1.12062	11.48593	5.12652
H	1.26578	6.79756	6.78149
H	2.70502	6.07682	6.04807
H	1.12066	5.73981	5.36861
H	2.41572	8.01909	0.76242
H	4.04079	8.25585	1.38405
H	3.06160	9.65838	0.92724
H	0.32580	11.39307	5.87250
H	0.76186	12.13741	4.32413
H	1.96078	12.00000	5.61007
H	-9.21925	-4.82415	-2.53740
H	-4.89697	-2.16687	4.71199
H	-4.35035	-3.54166	5.65811
H	-5.51451	-2.40076	6.35122
H	-9.35750	-5.90182	3.66258
H	5.82336	2.66117	0.18494
H	-6.63346	7.12750	-2.33529
H	-6.74048	5.72984	-1.27340
H	-8.21411	6.57194	-1.77470
H	9.09301	0.04167	0.00250
H	-7.78821	3.02314	-6.41009
H	-0.95796	5.95107	-2.33139
H	9.89499	2.66010	2.84486
H	9.86650	1.11183	1.99728
H	8.35616	1.86346	2.49682

Optimized geometry of 2-diMes$^{+\bullet}$, UB3LYP/6-31g(d,p)

Energy: -6727.9994443 a.u.

C	-9.28588	-2.81898	0.78753
C	-4.96289	0.70958	-0.18272
C	-8.36320	-2.47229	-0.22386
C	-4.95610	-0.74984	0.17467
C	-2.89395	-2.30208	0.82103
C	-3.27046	-3.44756	1.60127
C	-10.15307	-4.63238	-0.60990
C	-2.80455	-0.01112	-0.00802
C	-1.46911	-2.37192	0.61613
C	1.38071	0.00717	-0.00963
C	-8.34122	-3.20857	-1.43158
C	-4.46580	-3.65437	2.27164
H	-5.21070	-2.87286	2.16011
C	0.70704	-1.24343	-0.14483
C	-3.60509	1.10377	-0.37870
C	-3.59435	-1.13251	0.36569
C	-10.16165	-3.89088	0.57376
H	-10.86673	-4.15346	1.35930
C	-1.38069	-0.00521	-0.00977
C	-6.05536	-1.59916	0.11550
H	-5.82433	-2.65854	0.18629
C	-4.86453	-4.71579	3.12375
C	-4.50391	3.62260	-2.27759
H	-5.24288	2.83580	-2.16389
C	-7.42523	-1.30943	-0.04684
C	-1.49094	2.36063	-0.63494
C	2.80457	0.01296	-0.00770
C	-2.03601	-4.27406	1.76151
C	-2.91581	2.27943	-0.83570
C	-6.06933	1.54927	-0.11915
H	-5.84790	2.61063	-0.19073
C	-0.70698	1.24532	-0.14595
C	-0.69577	-1.24978	0.12567
C	-9.23511	-4.27074	-1.60089
C	-1.89863	-5.52205	2.38970
H	-0.90375	-5.95840	2.31162
C	-2.37943	-7.61336	3.62617
C	1.46897	2.37442	0.61436
C	-3.30391	3.42285	-1.61330
C	-6.37391	-3.75192	4.90505
C	0.69578	1.25186	0.12469
C	3.60529	-1.10225	-0.37730
C	-0.98489	-3.58277	1.17314
H	0.04419	-3.90960	1.18484
C	-7.43592	1.24751	0.04868
C	2.91613	-2.27847	-0.83337
C	-9.35244	-2.07015	2.10089
H	-8.35576	-1.85925	2.49832
H	-9.89498	-2.65481	2.84707
H	-9.86576	-1.10686	1.99895
C	-8.00603	-0.03345	0.00184
H	-9.09305	-0.03799	0.00373
C	-2.82806	-6.27365	3.10275
C	-4.91456	4.68331	-3.12489
C	3.59416	1.13472	0.36526
C	1.49111	-2.35910	-0.63280
C	-6.21423	-4.59887	3.78509
C	0.98468	3.58569	1.17037
H	-0.04439	3.91260	1.18159
C	-11.08845	-5.80238	-0.80650
H	-10.60312	-6.74836	-0.53535
H	-11.40384	-5.89100	-1.85066
H	-11.98431	-5.70698	-0.18617
C	4.96300	-0.70757	-0.18155
C	-4.14158	-5.86937	3.43359

H	-4.67396	-6.57362	4.06975
C	-1.86257	-7.72884	4.93389
C	-8.38431	2.40128	0.22921
C	4.46529	3.65760	2.26990
H	5.21016	2.87598	2.15897
C	2.89374	2.30464	0.81963
C	3.30414	-3.42315	-1.60935
C	-1.54508	-10.13159	4.60218
C	-7.30269	-5.37380	3.33274
C	1.01776	-3.57401	-1.18971
H	-0.00893	-3.90825	-1.20466
C	4.50454	-3.62519	-2.27245
H	5.24432	-2.83907	-2.15936
C	3.27012	3.45054	1.59928
C	-6.26817	4.56037	-3.77720
C	6.06978	-1.54675	-0.11748
H	5.84881	-2.60826	-0.18811
C	2.03569	4.27727	1.75865
C	4.95594	0.75214	0.17461
C	-7.35781	5.32711	-3.31413
C	7.43631	-1.24426	0.04938
C	-7.39574	-2.85374	-2.55925
H	-7.43877	-1.78656	-2.80002
H	-7.64536	-3.41684	-3.46226
H	-6.35271	-3.07686	-2.30897
C	-2.07629	4.25887	-1.77733
C	-1.01789	3.57527	-1.19301
H	0.00866	3.90992	-1.20795
C	-7.61253	-3.70483	5.55353
H	-7.72487	-3.05969	6.42226
C	-5.22529	-2.92163	5.43492
C	4.86381	4.71935	3.12164
C	-6.43081	3.71691	-4.89943
C	2.07605	-4.25877	-1.77282
C	-8.36212	3.14149	1.43459
C	6.21335	4.60274	3.78335
C	6.43629	-3.72602	-4.89070
C	-8.52388	-5.29789	4.01411
C	-9.31209	2.73994	-0.78012
C	7.30181	5.37772	3.33102
C	-8.70140	-4.47180	5.12680
C	4.91468	-4.68784	-3.11762
C	-7.17828	-6.28578	2.13182
H	-6.70903	-5.77579	1.28494
H	-8.16157	-6.63922	1.81168
H	-6.56412	-7.16698	2.35074
C	-2.06374	-9.99542	3.31109
H	-2.14899	-10.87532	2.67708
C	-8.76384	4.42441	-5.10209
C	6.05486	1.60185	0.11481
C	-9.26312	4.19737	1.60484
H	-9.24231	4.75816	2.53686
C	-7.23024	6.23568	-2.11100
C	-1.45601	-8.98533	5.39754
H	-1.05948	-9.06861	6.40712
C	6.26892	-4.56986	-3.76962
C	-4.20080	5.84204	-3.43696
H	-4.74152	6.54403	-4.06855
C	-2.48082	-8.75798	2.80757
C	7.42480	1.31261	-0.04760
C	-10.19210	4.54567	0.61923
C	5.29221	-2.89476	-5.42880
H	4.41791	-3.51422	-5.65604
H	5.58756	-2.37620	-6.34445
H	4.96041	-2.13810	-4.70945
C	-8.58339	5.24675	-3.98721
H	-9.41803	5.84422	-3.62722
C	8.00600	0.03686	0.00149
C	-2.88804	6.25422	-3.11311
C	1.89824	5.52559	2.38611

H	0.90343	5.96202	2.30745
C	-1.93984	7.71681	-4.94735
C	7.68033	-3.67822	-5.52878
H	7.80041	-3.03059	-6.39461
C	-7.67353	3.66542	-5.53954
C	4.14080	5.87310	3.43078
H	4.67294	6.57754	4.06693
C	1.94900	-5.50865	-2.39919
H	0.95616	-5.95036	-2.32531
C	6.37292	3.75609	4.90353
C	8.52287	5.30214	4.01263
H	9.35647	5.90610	3.66114
C	8.38546	-2.39754	0.22867
C	-2.45061	7.59721	-3.63761
C	-10.19536	3.80556	-0.56530
H	-10.90020	4.06597	-1.35174
C	-1.81354	6.51154	-5.85250
H	-1.19825	5.72804	-5.39740
H	-1.35862	6.78767	-6.80714
H	-2.78924	6.05972	-6.06390
C	8.70029	4.47630	5.12555
C	-1.95023	5.50811	-2.40529
C	2.82751	6.27740	3.09918
C	-1.74042	-6.52223	5.83783
H	-1.13254	-5.73481	5.37954
H	-1.27934	-6.79472	6.79056
H	-2.71821	-6.07693	6.05341
C	-7.40385	2.80198	2.55619
H	-6.36538	3.03683	2.29780
H	-7.43240	1.73512	2.80041
H	-7.65379	3.36470	3.45933
C	-2.55623	8.74068	-2.81794
C	4.19988	-5.84638	-3.42838
H	4.74050	-6.54992	-4.05832
C	-9.37158	1.99354	-2.09522

H	-9.87636	1.02555	-1.99516
H	-8.37316	1.79222	-2.49330
H	-9.91952	2.57491	-2.84004
C	7.61144	3.70930	5.55225
H	7.72370	3.06439	6.42115
C	2.88649	-6.25676	-3.10527
C	8.36229	2.47571	-0.22549
C	9.28526	2.82314	0.78541
C	10.19510	-4.54078	0.61559
C	2.44868	-7.59991	-3.62904
C	7.17751	6.28921	2.12971
H	6.71056	5.77805	1.28224
H	8.16062	6.64443	1.81098
H	6.56126	7.16925	2.34738
C	-1.07047	-11.47196	5.11291
H	-1.17868	-11.54741	6.19895
H	-0.00991	-11.63130	4.88099
H	-1.62841	-12.29460	4.65586
C	-5.28111	2.89528	-5.44029
H	-4.94278	2.14051	-4.72200
H	-4.41167	3.52134	-5.66812
H	-5.57389	2.37528	-6.35595
C	-1.54341	8.97621	-5.41189
H	-1.15155	9.06266	-6.42303
C	10.03307	4.39335	5.83246
H	10.62785	3.54920	5.46135
H	9.90581	4.24722	6.90952
H	10.62447	5.30052	5.67854
C	-10.03428	-4.38851	5.83349
H	-9.90715	-4.24374	6.91075
H	-10.62642	-5.29500	5.67839
H	-10.62823	-3.54341	5.46324
C	9.31228	-2.73494	-0.78188
C	-11.17723	5.66914	0.84255
H	-12.05032	5.32353	1.41017

H	-11.54472	6.07322	-0.10509
H	-10.72781	6.48852	1.41202
C	7.35636	-5.33802	-3.30334
C	-3.03635	-8.66978	1.40333
H	-3.07287	-9.65730	0.93639
H	-2.42686	-8.01857	0.76725
H	-4.04977	-8.25371	1.39506
C	8.36498	-3.13839	1.43371
C	-10.10087	4.33654	-5.80037
H	-10.68271	3.47950	-5.43850
H	-9.97999	4.20887	-6.88055
H	-10.70172	5.23408	-5.62793
C	10.19652	-3.80011	-0.56855
H	10.90063	-4.05964	-1.35595
C	5.22433	2.92575	5.43337
H	4.34900	3.54557	5.65557
H	5.51323	2.40566	6.35021
H	4.89675	2.17030	4.71080
C	2.13661	-9.98179	-3.30909
H	2.21455	-10.85870	-2.67003
C	1.93417	-7.71917	-4.93729
C	11.18122	-5.66370	0.83735
H	11.55001	-6.06530	-0.11084
H	10.73216	-6.48484	1.40459
H	12.05339	-5.31844	1.40658
C	-3.10555	8.64802	-1.41155
H	-4.11660	8.22632	-1.39959
H	-3.14568	9.63486	-0.94347
H	-2.48987	7.99958	-0.77860
C	2.54927	-8.74228	-2.80714
C	8.76589	-4.44514	-5.09344
C	9.35261	2.07483	2.09904
C	10.16060	3.89521	0.57086
H	10.86590	4.15835	1.35602
C	8.58317	-5.26156	-3.97443
H	9.41771	-5.85534	-3.60820
C	10.15134	4.63619	-0.61314
C	1.62793	-10.12312	-4.60366
C	9.26691	-4.19367	1.60244
H	9.24754	-4.75497	2.53420
C	8.33966	3.21144	-1.43352
C	3.09191	-8.64802	-1.39826
H	4.10391	-8.22870	-1.38278
H	3.12781	-9.63405	-0.92814
H	2.47505	-7.99687	-0.76927
C	7.22674	-6.23872	-2.09454
H	6.62654	-7.12965	-2.31315
H	6.73957	-5.72641	-1.25935
H	8.20974	-6.57666	-1.75737
C	10.08585	-4.40966	-5.82799
H	10.91446	-4.70288	-5.17693
H	10.29806	-3.41126	-6.22250
H	10.08250	-5.09936	-6.68135
C	-2.14930	9.98111	-3.32240
H	-2.23771	10.86007	-2.68752
C	-1.63673	10.12140	-4.61549
C	9.37002	-1.98761	-2.09652
H	9.88374	-1.02407	-1.99815
H	8.37095	-1.77711	-2.48809
H	9.90785	-2.57252	-2.84578
C	1.53196	-8.97771	-5.39934
H	1.13062	-9.06274	-6.40686
C	7.40760	-2.80010	2.55642
H	7.65889	-3.36299	3.45909
H	6.36904	-3.03565	2.29901
H	7.43557	-1.73331	2.80106
C	1.21809	-11.47673	-5.13513
H	2.06448	-11.98878	-5.60989
H	0.42958	-11.38868	-5.88834
H	0.85470	-12.12769	-4.33439

C	9.23316	4.27382	-1.60362
H	9.21682	4.82679	-2.54039
C	1.80247	-6.51315	-5.84066
H	2.77652	-6.05799	-6.05246
H	1.18517	-5.73205	-5.38408
H	1.34750	-6.78963	-6.79517
C	11.08629	5.80641	-0.81049
H	10.60164	6.75206	-0.53697
H	11.39923	5.89632	-1.85527
H	11.98359	5.71023	-0.19237
C	7.39398	2.85576	-2.56076
H	6.35090	3.07838	-2.31020
H	7.43752	1.78853	-2.80121
H	7.64299	3.41871	-3.46402
C	-1.17293	11.46512	-5.12733
H	-0.11247	11.63117	-4.89973
H	-1.73427	12.28386	-4.66746
H	-1.28614	11.54052	-6.21287
H	1.04048	9.07023	6.39533
C	1.44698	8.98842	5.38962
C	1.85830	7.73261	4.92823
C	1.72915	6.52502	5.82989
C	2.37953	7.61747	3.62225
C	2.47756	8.76119	2.80194
C	3.02719	8.67140	1.39548
C	2.05574	9.99792	3.30319
H	2.13183	10.87590	2.66536
C	1.54033	10.13517	4.59551
C	1.12062	11.48593	5.12652
H	1.26578	6.79756	6.78149
H	2.70502	6.07682	6.04807
H	1.12066	5.73981	5.36861
H	2.41572	8.01909	0.76242
H	4.04079	8.25585	1.38405
H	3.06161	9.65839	0.92724
H	0.32580	11.39307	5.87250
H	0.76186	12.13741	4.32413
H	1.96078	12.00000	5.61007
H	-9.21926	-4.82415	-2.53740
H	-4.89697	-2.16687	4.71199
H	-4.35035	-3.54166	5.65811
H	-5.51451	-2.40076	6.35122
H	-9.35750	-5.90182	3.66258
H	5.82336	2.66117	0.18494
H	-6.63346	7.12750	-2.33529
H	-6.74048	5.72984	-1.27340
H	-8.21411	6.57194	-1.77470
H	9.09301	0.04167	0.00250
H	-7.78821	3.02314	-6.41009
H	-0.95796	5.95107	-2.33139
H	9.89499	2.66010	2.84486
H	9.86650	1.11183	1.99728
H	8.35616	1.86346	2.49682

Optimized geometry of [2-diMes]$^{2+}$, B3LYP/6-31g(d,p)

Energy: -6727.823369 a. u.

C	-9.34038	-2.75599	0.67698
C	-4.97224	0.72000	-0.16262
C	-8.36371	-2.42952	-0.29054
C	-4.97299	-0.71213	0.15953
C	-2.91888	-2.26691	0.82384
C	-3.32111	-3.44461	1.57447
C	-10.11957	-4.59701	-0.73636
C	-2.79490	0.00285	-0.00033
C	-1.48011	-2.35196	0.63357
C	1.38054	-0.00013	0.00026
C	-8.26825	-3.18390	-1.48499
C	-4.51908	-3.63508	2.22972
H	-5.24891	-2.83803	2.13656
C	0.70538	-1.24862	-0.14213
C	-3.58819	1.11916	-0.36710
C	-3.58966	-1.11251	0.36577
C	-10.19386	-3.83810	0.43401
H	-10.93588	-4.09403	1.18632
C	-1.37878	0.00186	0.00021
C	-6.06515	-1.56511	0.08536
H	-5.83656	-2.62406	0.14760
C	-4.94780	-4.71145	3.06291
C	-4.51253	3.64422	-2.23054
H	-5.24369	2.84827	-2.13835
C	-7.43560	-1.27081	-0.08414
C	-1.47666	2.35596	-0.63253
C	2.79667	-0.00124	-0.00017
C	-2.10610	-4.27705	1.71399
C	-2.91544	2.27291	-0.82406
C	-6.06433	1.57335	-0.08974
H	-5.83543	2.63226	-0.15139
C	-0.70360	1.25021	-0.14394
C	-0.70538	-1.24744	0.14485
C	-9.15305	-4.24650	-1.68488
C	-1.99046	-5.55049	2.31663
H	-1.00471	-6.00286	2.22628
C	-2.51373	-7.65508	3.50143
C	1.48180	2.35449	0.63049
C	-3.31537	3.45159	-1.57443
C	-6.45874	-3.73799	4.83263
C	0.70714	1.24937	0.14309
C	3.59001	-1.11802	-0.36555
C	-1.03379	-3.58977	1.15652
H	-0.01255	-3.93838	1.17809
C	-7.43502	1.27916	0.07808
C	2.91730	-2.27245	-0.82104
C	-9.48879	-1.99084	1.97474
H	-8.52534	-1.66964	2.37871
H	-9.97392	-2.61572	2.72741
H	-10.10479	-1.09229	1.85031
C	-8.01473	0.00430	-0.00382
H	-9.09996	0.00470	-0.00473
C	-2.92971	-6.29535	3.00659
C	-4.93895	4.72171	-3.06345
C	3.59133	1.11453	0.36489
C	1.47846	-2.35498	-0.62923
C	-6.30313	-4.57695	3.70610
C	1.03533	3.59292	1.15180
H	0.01409	3.94159	1.17257
C	-11.03680	-5.77471	-0.96169
H	-10.57252	-6.70580	-0.61372
H	-11.26563	-5.90500	-2.02335
H	-11.97865	-5.65787	-0.41854
C	4.97404	-0.71851	-0.16129

C	-4.24573	-5.87259	3.35091	C	-8.27036	3.19053	1.48081
H	-4.78405	-6.58108	3.97609	C	6.30297	4.58170	3.70408
C	-2.03735	-7.81265	4.81964	C	6.45026	-3.75959	-4.83171
C	-8.36460	2.43655	0.28549	C	-8.63016	-5.22079	3.88371
C	4.52000	3.63890	2.22701	C	-9.33834	2.76490	-0.68367
H	5.24990	2.84178	2.13491	C	7.39917	5.32653	3.22233
C	2.92048	2.26952	0.82144	C	-8.80674	-4.40292	5.00259
C	3.31688	-3.45238	-1.56988	C	4.93881	-4.72549	-3.05813
C	-1.74902	-10.20968	4.42619	C	-7.27502	-6.22891	2.01952
C	-7.39904	-5.32211	3.22423	H	-6.74776	-5.73965	1.19489
C	1.02963	-3.59293	-1.14934	H	-8.26256	-6.52611	1.65900
H	0.00779	-3.93984	-1.16939	H	-6.71925	-7.14422	2.25445
C	4.51381	-3.64671	-2.22609	C	-2.22914	-10.02897	3.12544
H	5.24569	-2.85128	-2.13504	H	-2.31230	-10.88934	2.46531
C	3.32243	3.44793	1.57113	C	-8.79722	4.42157	-5.00559
C	-6.29415	4.58991	-3.70742	C	6.06679	1.56695	0.08508
C	6.06619	-1.57167	-0.08776	C	-9.15623	4.25157	1.68099
H	5.83739	-2.63062	-0.14893	H	-9.09002	4.81969	2.60585
C	2.10741	4.28060	1.70920	C	-7.26369	6.24314	-2.02071
C	4.97467	0.71393	0.15950	C	-1.66359	-9.08837	5.25662
C	-7.38889	5.33684	-3.22569	H	-1.29731	-9.20706	6.27374
C	7.43688	-1.27726	0.07990	C	6.29307	-4.59712	-3.70483
C	-7.26742	-2.84672	-2.57028	C	-4.23487	5.88187	-3.35052
H	-7.26922	-1.77852	-2.80931	H	-4.77164	6.59144	-3.97579
H	-7.49914	-3.39646	-3.48550	C	-2.61223	-8.77200	2.64529
H	-6.24241	-3.10840	-2.28452	C	7.43732	1.27265	-0.08363
C	-2.09910	4.28242	-1.71249	C	-10.12641	4.59860	0.73425
C	-1.02823	3.59342	-1.15441	C	5.29396	-2.95954	-5.39086
H	-0.00649	3.94063	-1.17497	H	4.44445	-3.60338	-5.64358
C	-7.70709	-3.66833	5.45853	H	5.59501	-2.42840	-6.29703
H	-7.81855	-3.03216	6.33358	H	4.92286	-2.21456	-4.67808
C	-5.29847	-2.94701	5.39643	C	-8.61983	5.23825	-3.88600
C	4.94809	4.71576	3.05986	H	-9.46076	5.81807	-3.51294
C	-6.45065	3.75190	-4.83457	C	8.01649	-0.00237	-0.00248
C	2.10019	-4.28299	-1.70658	C	-2.91844	6.30244	-3.00512

C	1.99142	5.55443	2.31087
H	1.00568	6.00666	2.21965
C	-2.02034	7.81825	-4.81657
C	7.69842	-3.69499	-5.45931
H	7.81314	-3.05480	-6.33097
C	-7.69870	3.68511	-5.46133
C	4.24580	5.87705	3.34682
H	4.78353	6.58577	3.97223
C	1.98090	-5.55713	-2.30687
H	0.99410	-6.00686	-2.21477
C	6.45787	3.74355	4.83129
C	8.62989	5.22566	3.88259
H	9.47187	5.80396	3.50956
C	8.36665	-2.43446	0.28703
C	-2.49982	7.66157	-3.49937
C	-10.19333	3.84634	-0.44038
H	-10.92935	4.10745	-1.19667
C	-1.90423	6.63913	-5.75675
H	-1.27308	5.84808	-5.33737
H	-1.47137	6.94544	-6.71205
H	-2.88087	6.18661	-5.96324
C	8.80580	4.40852	5.00212
C	-1.98098	5.55590	-2.31456
C	2.93014	6.29971	3.00115
C	-1.92178	-6.63390	5.76035
H	-1.28981	-5.84300	5.34195
H	-1.49015	-6.94074	6.71604
H	-2.89844	-6.18102	5.96593
C	-7.26544	2.85747	2.56357
H	-6.24196	3.12235	2.27535
H	-7.26329	1.78931	2.80287
H	-7.49687	3.40658	3.47923
C	-2.59893	8.77880	-2.64374
C	4.23390	-5.88569	-3.34321
H	4.76997	-6.59622	-3.96800

C	-9.47923	2.00618	-1.98600
H	-10.09330	1.10538	-1.86844
H	-8.51342	1.68954	-2.38794
H	-9.96329	2.63351	-2.73731
C	7.70587	3.67430	5.45796
H	7.81681	3.03875	6.33353
C	2.91752	-6.30523	-2.99681
C	8.36539	2.43133	-0.29038
C	9.34152	2.75860	0.67743
C	10.12926	-4.59603	0.73501
C	2.49792	-7.66425	-3.49040
C	7.27567	6.23227	2.01677
H	6.75228	5.74073	1.19100
H	8.26323	6.53235	1.65874
H	6.71616	7.14587	2.24944
C	-1.31276	-11.57203	4.91109
H	-1.42008	-11.66342	5.99570
H	-0.25823	-11.75733	4.67175
H	-1.89576	-12.36928	4.44076
C	-5.29149	2.95908	-5.39806
H	-4.91401	2.21604	-4.68661
H	-4.44634	3.60764	-5.65331
H	-5.59254	2.42640	-6.30333
C	-1.64403	9.09340	-5.25300
H	-1.27539	9.21142	-6.26934
C	10.14768	4.30056	5.68726
H	10.70534	3.42578	5.33008
H	10.03517	4.18955	6.76998
H	10.76593	5.18200	5.49608
C	-10.14900	-4.29455	5.68691
H	-10.03712	-4.18342	6.76968
H	-10.76735	-5.17587	5.49548
H	-10.70623	-3.41967	5.32928
C	9.34158	-2.76108	-0.68153
C	-11.08558	5.73581	0.99066

H	-11.90898	5.41511	1.64050
H	-11.52698	6.10437	0.06084
H	-10.58863	6.57249	1.49100
C	7.38807	-5.34339	-3.22152
C	-3.13100	-8.63570	1.23076
H	-3.17798	-9.61048	0.73938
H	-2.49123	-7.98500	0.62438
H	-4.13556	-8.19880	1.20821
C	8.27161	-3.18996	1.48140
C	-10.13918	4.31629	-5.69100
H	-10.69722	3.44065	-5.33650
H	-10.02674	4.20850	-6.77406
H	-10.75699	5.19745	-5.49717
C	10.19689	-3.84240	-0.43865
H	10.93381	-4.10218	-1.19454
C	5.29722	2.95301	5.39491
H	4.45333	3.60321	5.65016
H	5.59732	2.41984	6.30020
H	4.91829	2.21064	4.68353
C	2.19486	-10.03498	-3.10903
H	2.26497	-10.89333	-2.44476
C	2.01670	-7.82039	-4.80698
C	11.08877	-5.73301	0.99113
H	11.53183	-6.09987	0.06143
H	10.59161	-6.57078	1.48944
H	11.91096	-5.41278	1.64273
C	-3.12109	8.64343	-1.23037
H	-4.12654	8.20844	-1.21010
H	-3.16733	9.61826	-0.73904
H	-2.48390	7.99146	-0.62264
C	2.58853	-8.78013	-2.63200
C	8.79428	-4.43729	-5.00765
C	9.48936	1.99436	1.97579
C	10.19499	3.84064	0.43416
H	10.93658	4.09719	1.18667

C	8.61846	-5.24729	-3.88242
H	9.46098	-5.82164	-3.50448
C	10.12122	4.59874	-0.73678
C	1.71662	-10.21550	-4.41058
C	9.15791	-4.25065	1.68123
H	9.09120	-4.81985	2.60539
C	8.27051	3.18483	-1.48542
C	3.10260	-8.64367	-1.21575
H	4.10837	-8.20973	-1.19063
H	3.14526	-9.61806	-0.72322
H	2.46290	-7.99043	-0.61200
C	7.26392	-6.24093	-2.00990
H	6.70170	-7.15441	-2.23648
H	6.74268	-5.74313	-1.18651
H	8.25098	-6.54139	-1.65107
C	10.11865	-4.38500	-5.73218
H	10.95205	-4.61045	-5.06072
H	10.29295	-3.40097	-6.17718
H	10.15011	-5.11875	-6.54710
C	-2.21318	10.03519	-3.12330
H	-2.29676	10.89581	-2.46355
C	-1.72988	10.21501	-4.42299
C	9.48401	-2.00068	-1.98270
H	10.10646	-1.10561	-1.86499
H	8.51965	-1.67440	-2.38025
H	9.95954	-2.63013	-2.73760
C	1.63234	-9.09406	-5.24089
H	1.25556	-9.21031	-6.25442
C	7.26555	-2.85871	2.56370
H	7.49725	-3.40785	3.47927
H	6.24261	-3.12496	2.27484
H	7.26174	-1.79066	2.80342
C	1.32712	-11.58609	-4.91185
H	2.19521	-12.11763	-5.32097
H	0.58017	-11.52326	-5.70835

H	0.91924	-12.20521	-4.10738
C	9.15527	4.24742	-1.68557
H	9.09242	4.81147	-2.61309
C	1.89914	-6.64081	-5.74643
H	2.87532	-6.18701	-5.95219
H	1.26688	-5.85066	-5.32696
H	1.46712	-6.94715	-6.70210
C	11.03839	5.77643	-0.96237
H	10.57433	6.70748	-0.61398
H	11.26670	5.90689	-2.02412
H	11.98050	5.65945	-0.41971
C	7.27041	2.84666	-2.57107
H	6.24514	3.10821	-2.28612
H	7.27269	1.77830	-2.80940
H	7.50252	3.39586	-3.48652
C	-1.29073	11.57667	-4.90723
H	-0.23597	11.75984	-4.66728
H	-1.87245	12.37490	-4.43700
H	-1.39725	11.66859	-5.99188
H	1.27509	9.20753	6.26003
C	1.65167	9.09076	5.24648
C	2.03298	7.81633	4.81212
C	1.91299	6.63684	5.75135
C	2.51387	7.65950	3.49552
C	2.60724	8.77551	2.63755
C	3.12099	8.63836	1.22126
H	10.10557	1.09582	1.85228
H	8.52576	1.67324	2.37947
C	2.21662	10.03113	3.11505
H	2.28885	10.88956	2.45110
C	1.73875	10.21232	4.41664
C	1.35255	11.58367	4.91841
H	1.47836	6.94323	6.70582
H	2.88880	6.18348	5.95991
H	1.28217	5.84641	5.33032
H	2.47998	7.98654	0.61737
H	4.12590	8.20242	1.19601
H	3.16558	9.61279	0.72896
H	0.60577	11.52233	5.71519
H	0.94576	12.20391	4.11426
H	2.22201	12.11313	5.32731
H	-9.08975	-4.81124	-2.61194
H	-4.91911	-2.20506	4.68484
H	-4.45471	-3.59701	5.65263
H	-5.59915	-2.41328	6.30120
H	-9.47190	-5.79937	3.51056
H	5.83804	2.62591	0.14641
H	-6.71111	7.16007	-2.25696
H	-6.73249	5.75488	-1.19804
H	-8.25074	6.53741	-1.65675
H	9.10172	-0.00273	-0.00337
H	-7.81087	3.04975	-6.33688
H	-0.99460	6.00671	-2.22320
H	9.97401	2.61983	2.72829

References

1 Y. Huang, M. K. Brown, *Angew. Chem. Int. Ed.* **2019**, *58*, 6048.

2 M. J. Frisch, G. W. Trucks, H. B. Schlegel, G. E. Scuseria, M. A. Robb, J. R. Cheeseman, G. Scalmani, V. Barone, G. A. Petersson, H. Nakatsuji, X. Li, M. Caricato, A. V Marenich, J. Bloino, B. G. Janesko, R. Gomperts, B. Mennucci, H. P. Hratchian, J. V Ortiz, A. F. Izmaylov, J. L. Sonnenberg, D. Williams-Young, F. Ding, F. Lipparini, F. Egidi, J. Goings, B. Peng, A. Petrone, T. Henderson, D. Ranasinghe, V. G. Zakrzewski, J. Gao, N. Rega, G. Zheng, W. Liang, M. Hada, M. Ehara, K. Toyota, R. Fukuda, J. Hasegawa, M. Ishida, T. Nakajima, Y. Honda, O. Kitao, H. Nakai, T. Vreven, K. Throssell, J. A. Montgomery Jr., J. E. Peralta, F. Ogliaro, M. J. Bearpark, J. J. Heyd, E. N. Brothers, K. N. Kudin, V. N. Staroverov, T. A. Keith, R. Kobayashi, J. Normand, K. Raghavachari, A. P. Rendell, J. C. Burant, S. S. Iyengar, J. Tomasi, M. Cossi, J. M. Millam, M. Klene, C. Adamo, R. Cammi, J. W. Ochterski, R. L. Martin, K. Morokuma, O. Farkas, J. B. Foresman, D. J. Fox, *Gaussian16 Revision C.01*, **2016**.

3 A. D. Becke, *J. Chem. Phys.* **1993**, *98*, 5648.

4 P. J. Stephens, F. J. Devlin, C. F. Chabalowski, M. J. Frisch, *J. Phys. Chem.* **1994**, *98*, 11623.

5 T. Lu, F. Chen, *J. Comput. Chem.* **2012**, *33*, 580.

6 Z. Wang, *Chemistry* **2024**, *6*, 1692.

7 *https://www.iqmol.org*.

8 K. Momma, F. Izumi, *J. Appl. Crystallogr.* **2011**, *44*, 1272.

9 L. J. Bourhis, O. V. Dolomanov, R. J. Gildea, J. A. K. Howard, H. Puschmann, *Acta Crystallogr. A* **2015**, *71*, 59.

10 G. M. Sheldrick, *Acta Crystallogr. A* **2008**, *64*, 112.

11 G. M. Sheldrick, *Acta Crystallogr. C* **2015**, *71*, 3.

12 http://supramolecular.org.

13 P. Thordarson, *Chem. Soc. Rev.* **2011**, *40*, 1305.

14 D. Brynn Hibbert, P. Thordarson, *Chem. Commun.* **2016**, *52*, 12792.
